# Supplementary figures and images for: Glutamatergic projections from the substantia nigra pars reticulata to the dorsal raphe nucleus regulate male social hierarchies
Source: PLoS Biol. 2026 Mar 3;24(3):e3003687. doi: 10.1371/journal.pbio.3003687 (PMC12974815; doi:10.1371/journal.pbio.3003687)

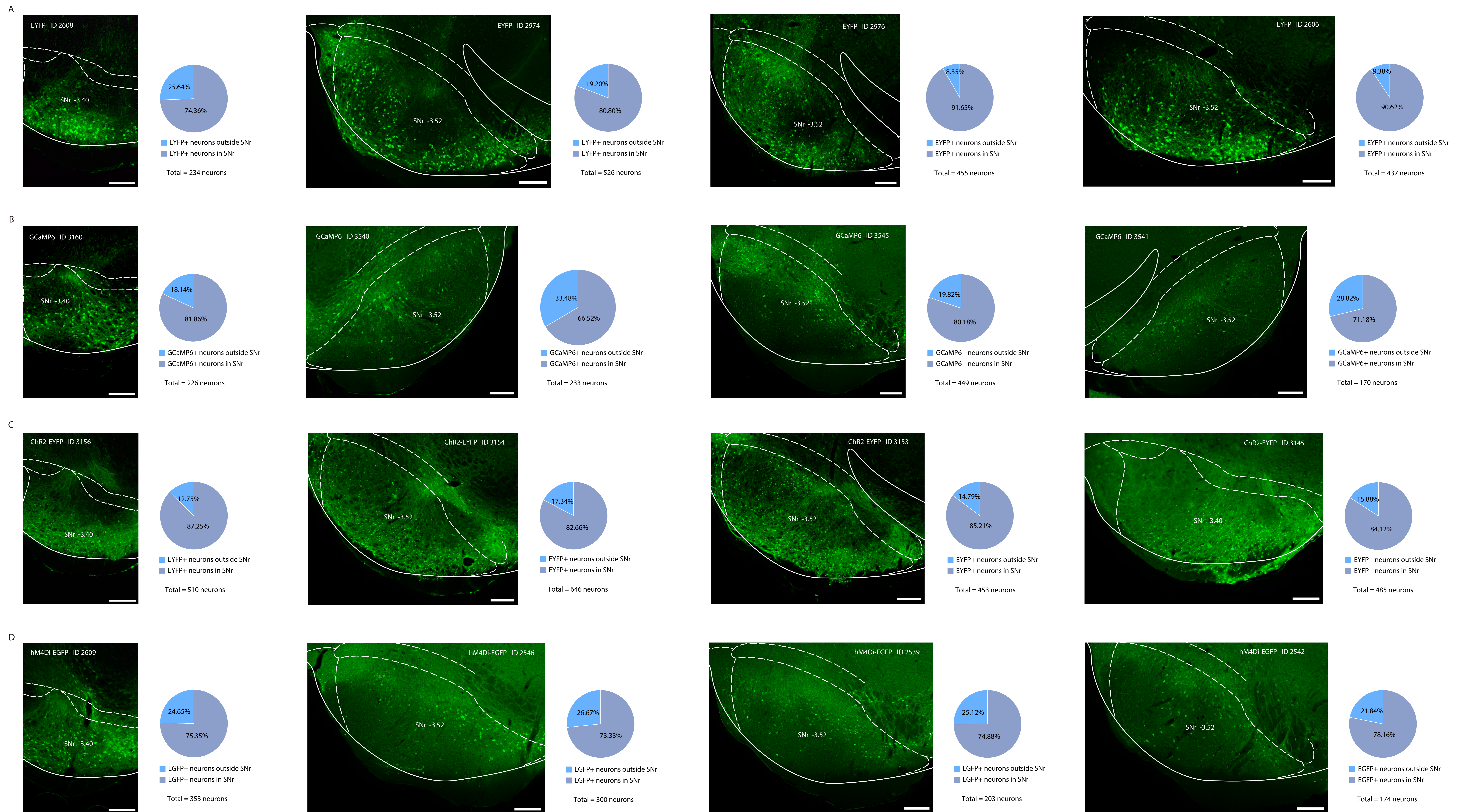

Supplement: S1 Fig — (A) Histological data from four individual animals, with each column representing a separate subject. The left side of each column displays a representative coronal section of the SNr illustrating the expression of EYFP; the right pie presents the percentage of SNr neurons expressing the virus relative to the total number of virus-expressing neurons in the entire field of view. (B–D) Similar analyses for GCaMP6, ChR2-EYFP, and hM4Di-EGFP expression, respectively. Scale bar, 200 μm. (PDF) [file pbio.3003687.s001.pdf]

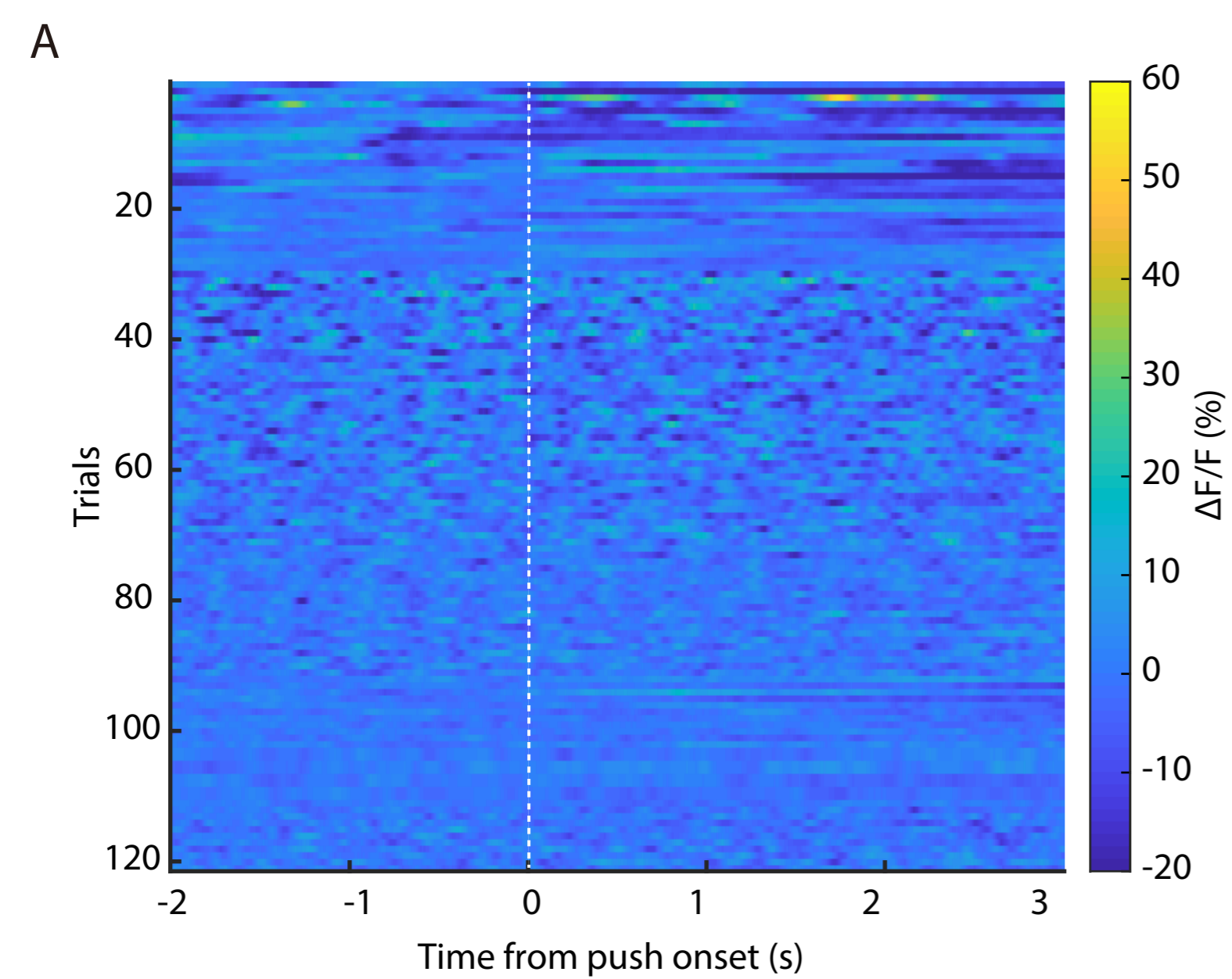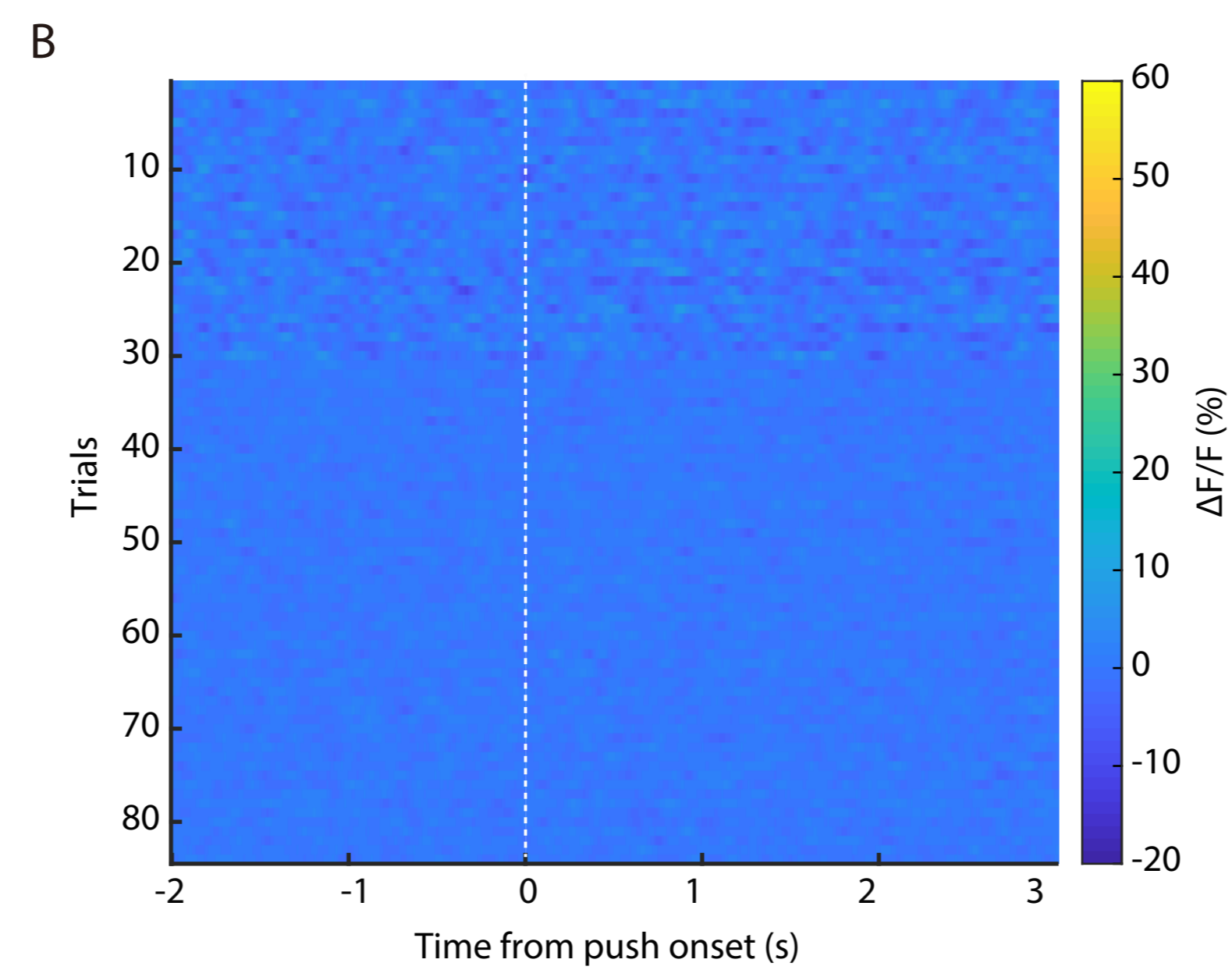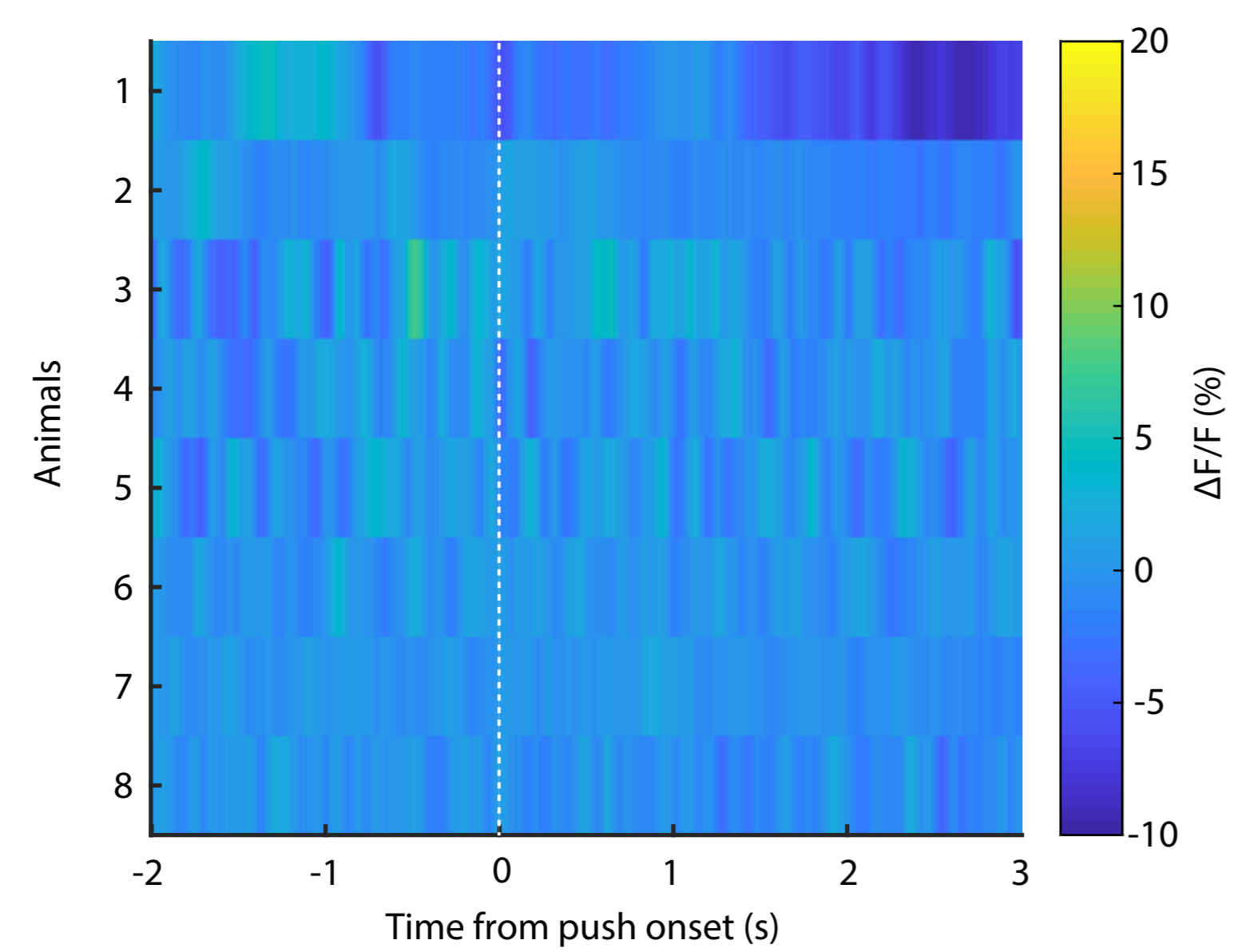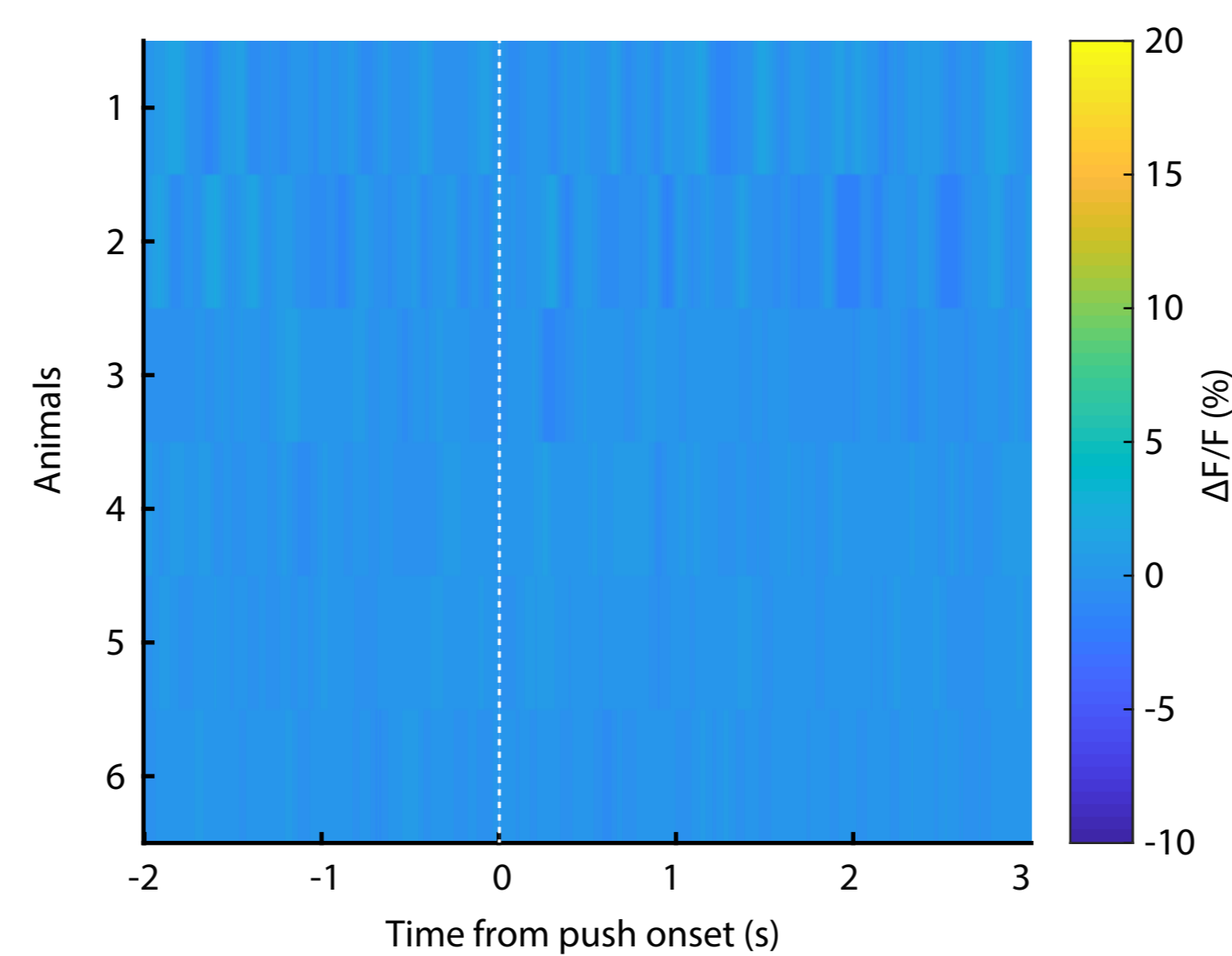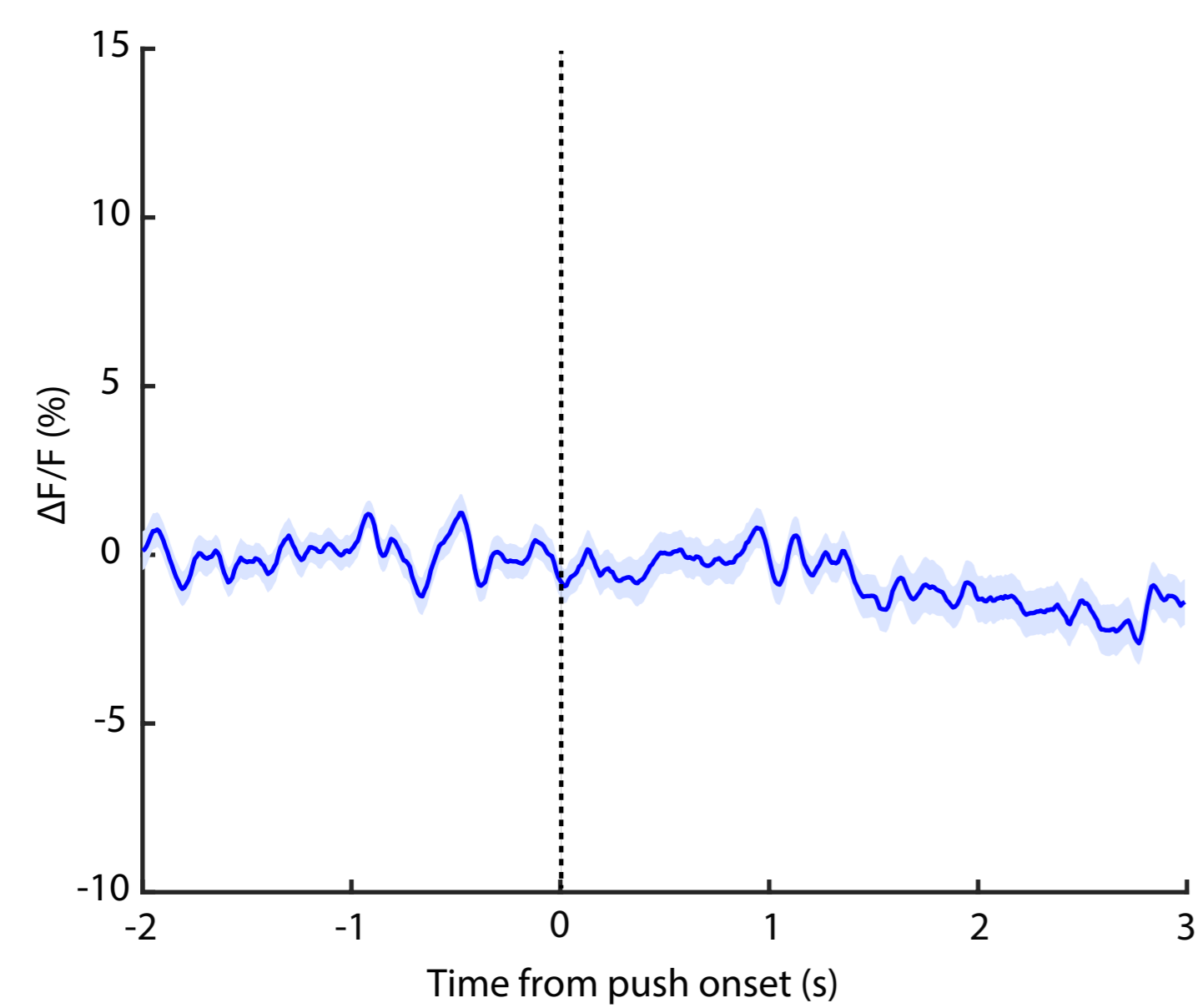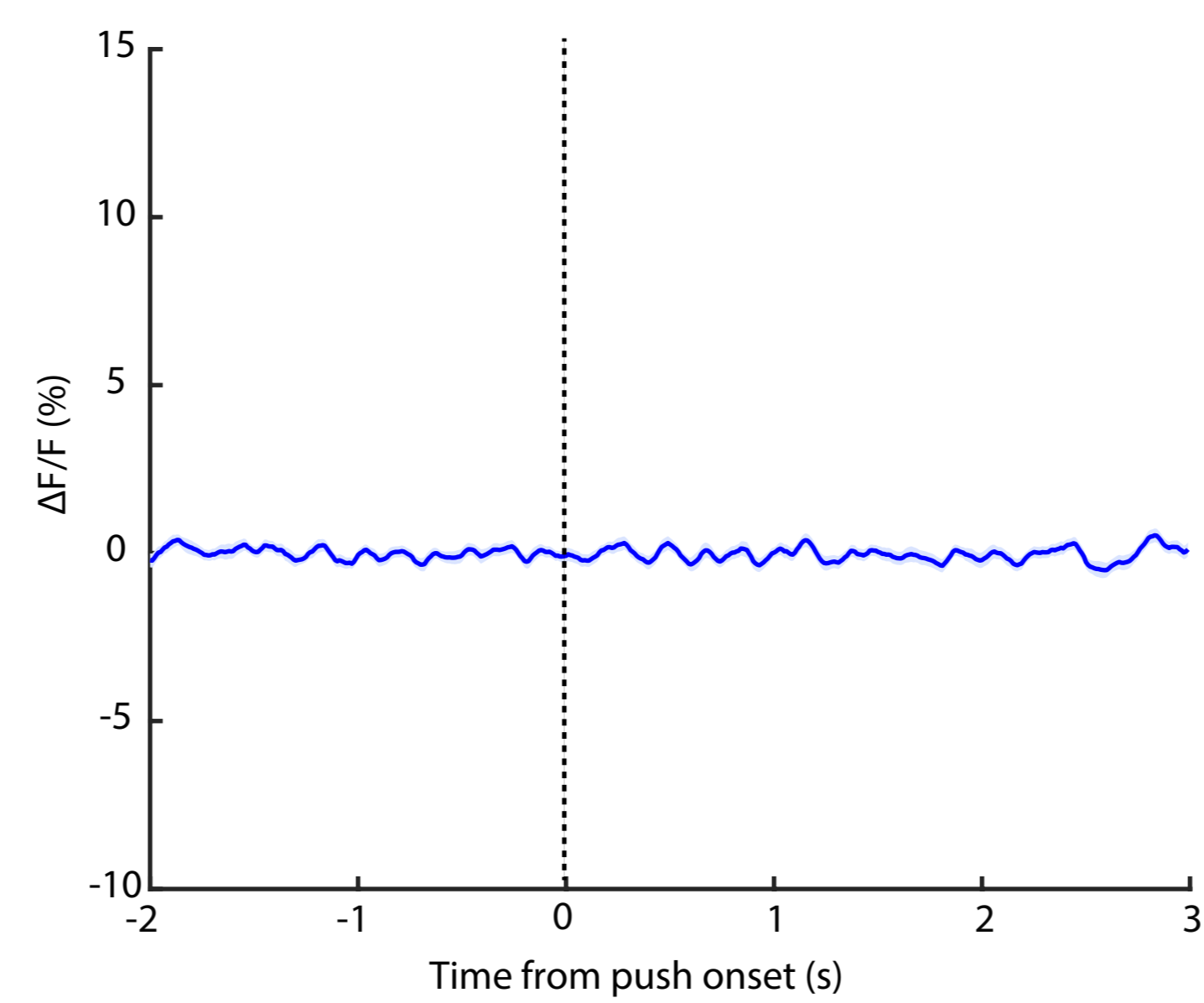

Supplement: S4 Fig — (A) Ca2+ signals were aligned with the onset of pushes when mice expressing GCaMP6f pushed the diamond-shaped rubber bouncy ball within the tube. The upper panel illustrates a heatmap of Ca2+ signals aligned to the push-initiation, with each row representing an individual trial (n = 121 trials from 8 mice). The middle panel depicts a heatmap displaying the average Ca2+ signals for each animal across all trials (n = 8). The lower panel presents the mean Ca2+ transients associated with pushes for the entire test group (n = 8), where solid lines indicate the mean and shaded areas represent SEM. No significant changes in calcium signals associated with pushes were observed in GCaMP6f-expressing mice (p > 0.05; permutation test). (B) Ca2+ signals were aligned with the initiation of pushes when mice expressing EYFP encountered the ball within the tube. The upper panel presents data from all trials (n = 84 trials from 6 mice), the middle panel shows data from all animals (n = 6), and the lower panel displays the mean ± SEM of the average Ca2+ signals. No significant changes in calcium signals associated with pushes were observed in EYFP-expressing mice (p > 0.05; permutation test). The data underlying this Figure can be found in files numbered 19–20 on Dryad (https://doi.org/10.5061/dryad.m0cfxppg3). (PDF) [file pbio.3003687.s004.pdf]

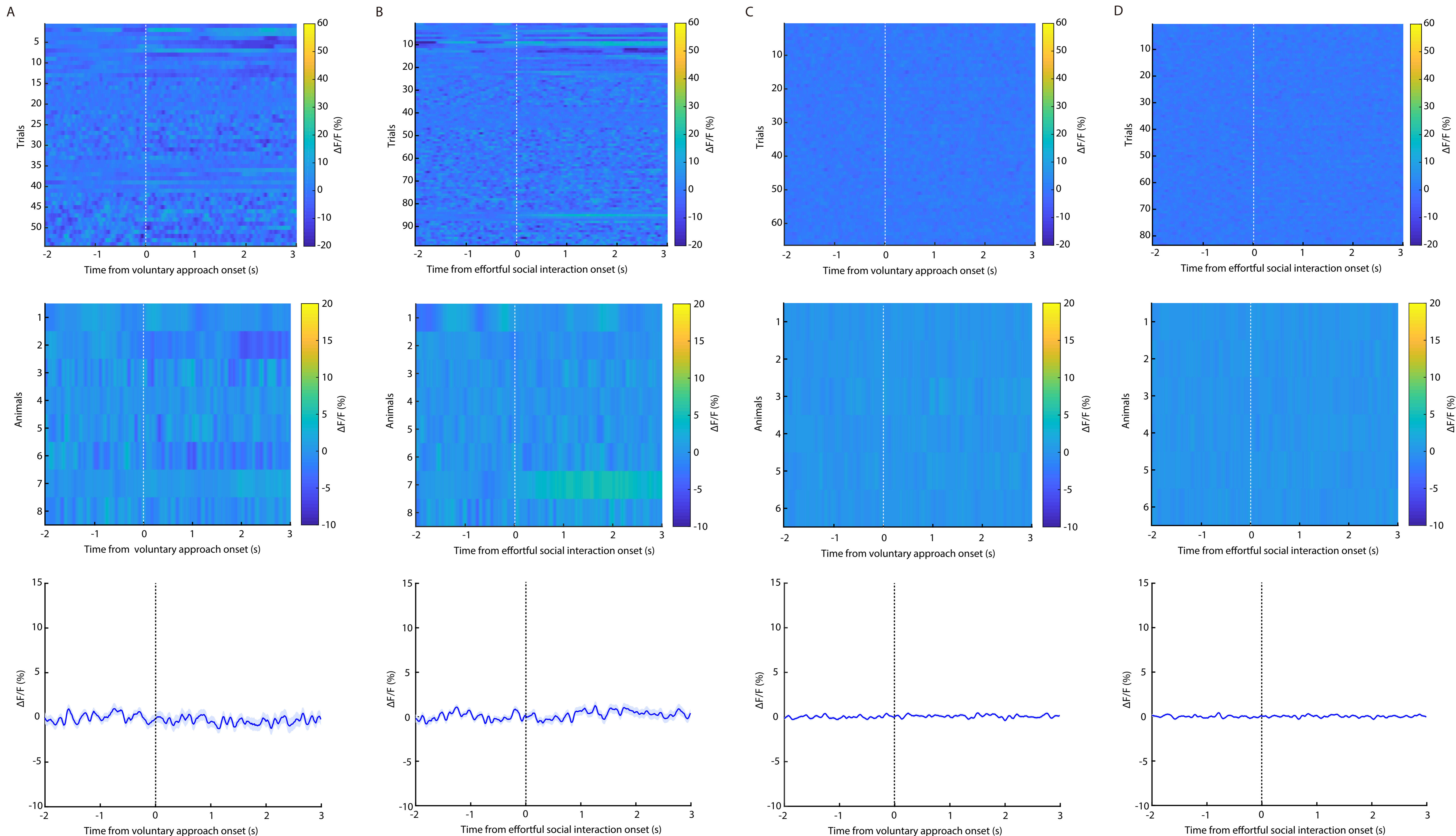

Supplement: S5 Fig — (A) Ca2+ signals were aligned with the initiation of voluntary approach when mice expressing GCaMP6f encountered a novel mouse introduced into the restraint cage. The upper panel presents a heatmap of Ca2+ signals aligned to the onset of voluntary approach, with each row representing an individual trial (n = 54 trials from 8 mice). The middle panel displays a heatmap depicting the average Ca2+ signals for each animal across all trials (n = 8). The lower panel illustrates the mean Ca2+ transients associated with voluntary approach for the entire test group (n = 8), where solid lines indicate the mean and shaded areas denote SEM. No significant changes in calcium signals associated with voluntary approach were observed in GCaMP6f-expressing mice (p > 0.05; permutation test). (B) Ca2+ signals were aligned with the onset of effortful social interaction when mice expressing GCaMP6f encountered the novel mouse in the restraint cage. The upper panel presents data from all trials (n = 98 trials from 8 mice), the middle panel summarizes data from all animals (n = 8), and the lower panel shows the mean ± SEM of the average Ca2+ signals. No significant changes in calcium signals associated with effortful social interaction were observed (p > 0.05; permutation test). Panels (C) and (D) replicate the findings presented in (A) and (B), respectively, with the only distinction being that (C) and (D) pertain to EYFP-expressing mice, involving 66 trials from 6 animals for (C) and 83 trials from 6 animals for (D). No significant changes in calcium signals were detected for EYFP-expressing mice (p > 0.05; permutation test). The data underlying this Figure can be found in files numbered 21–24 on Dryad (https://doi.org/10.5061/dryad.m0cfxppg3). (PDF) [file pbio.3003687.s005.pdf]

A

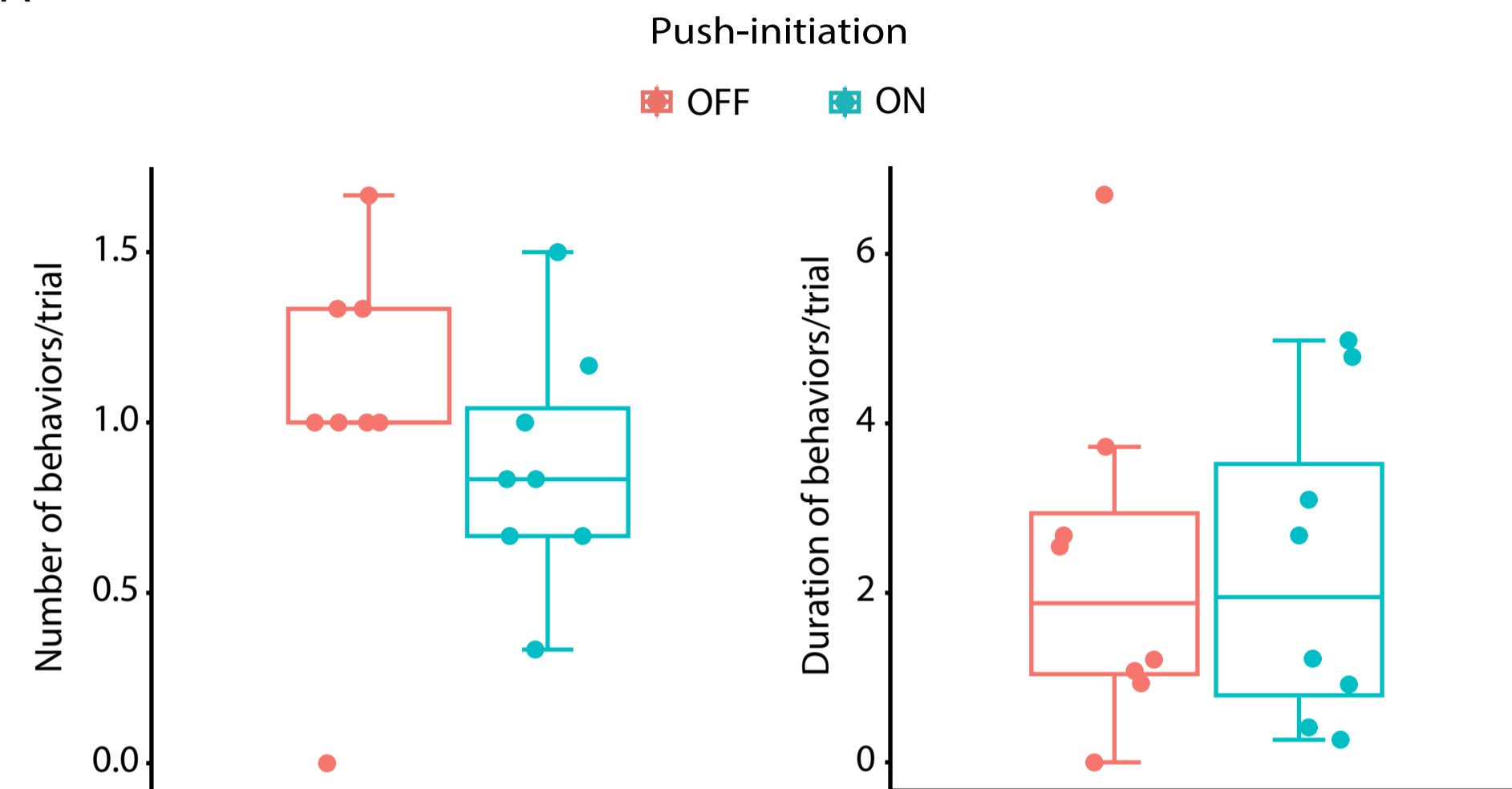

B

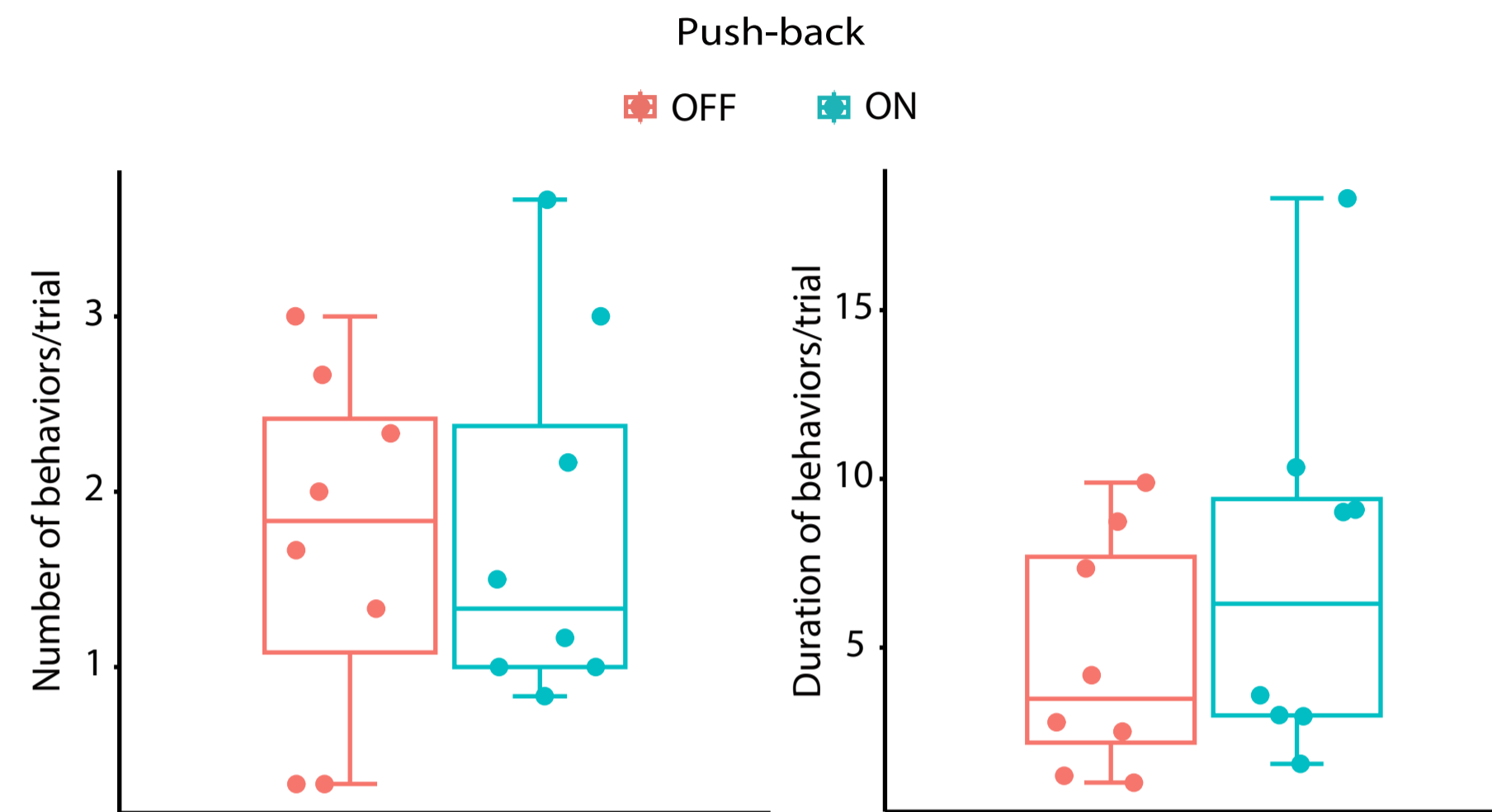

C

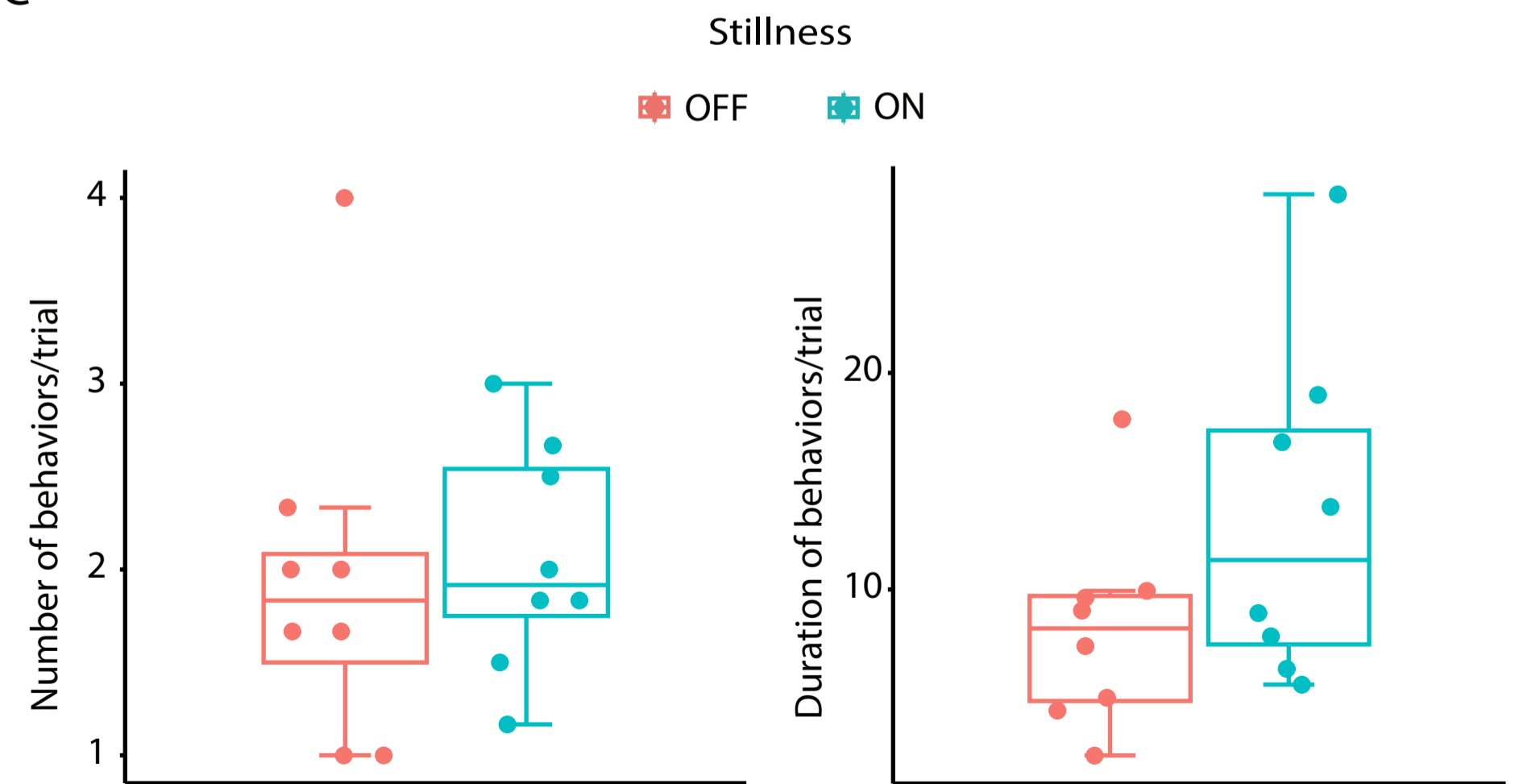

D

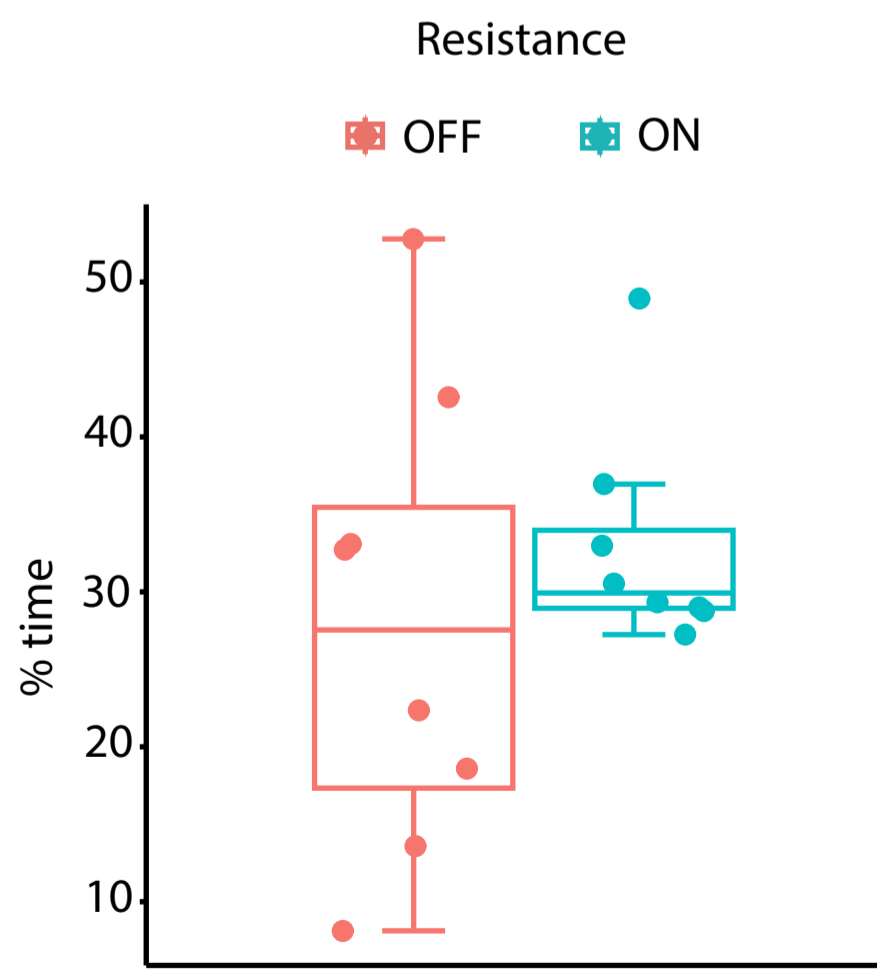

E

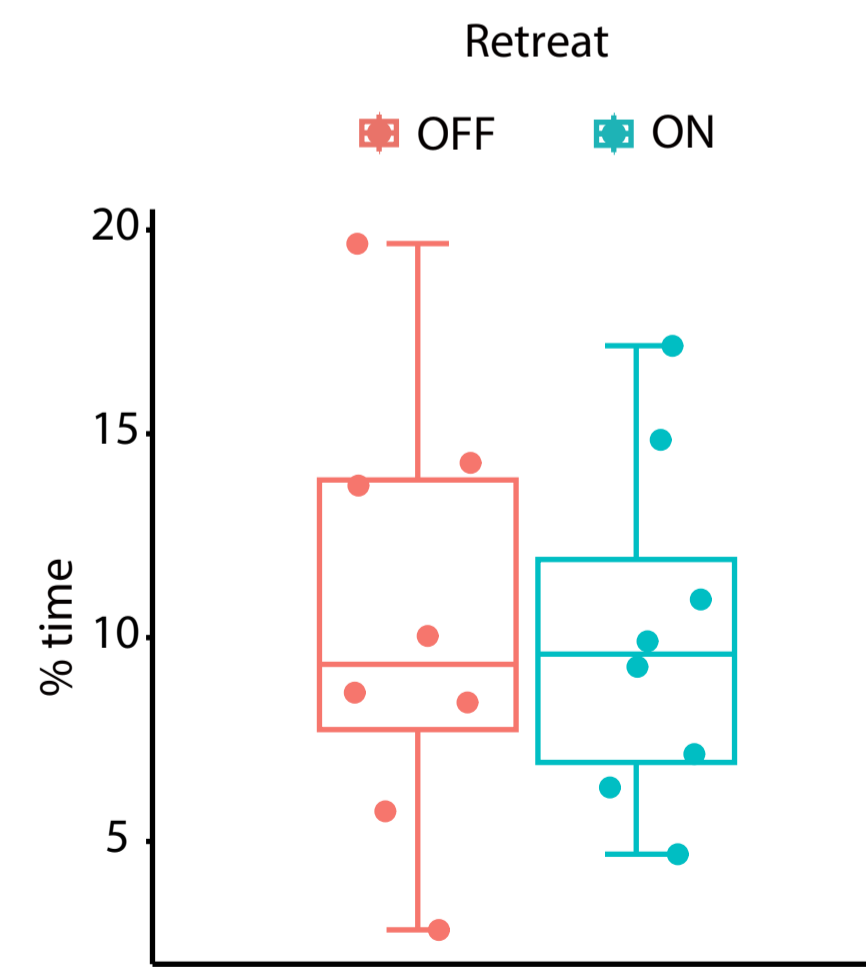

F

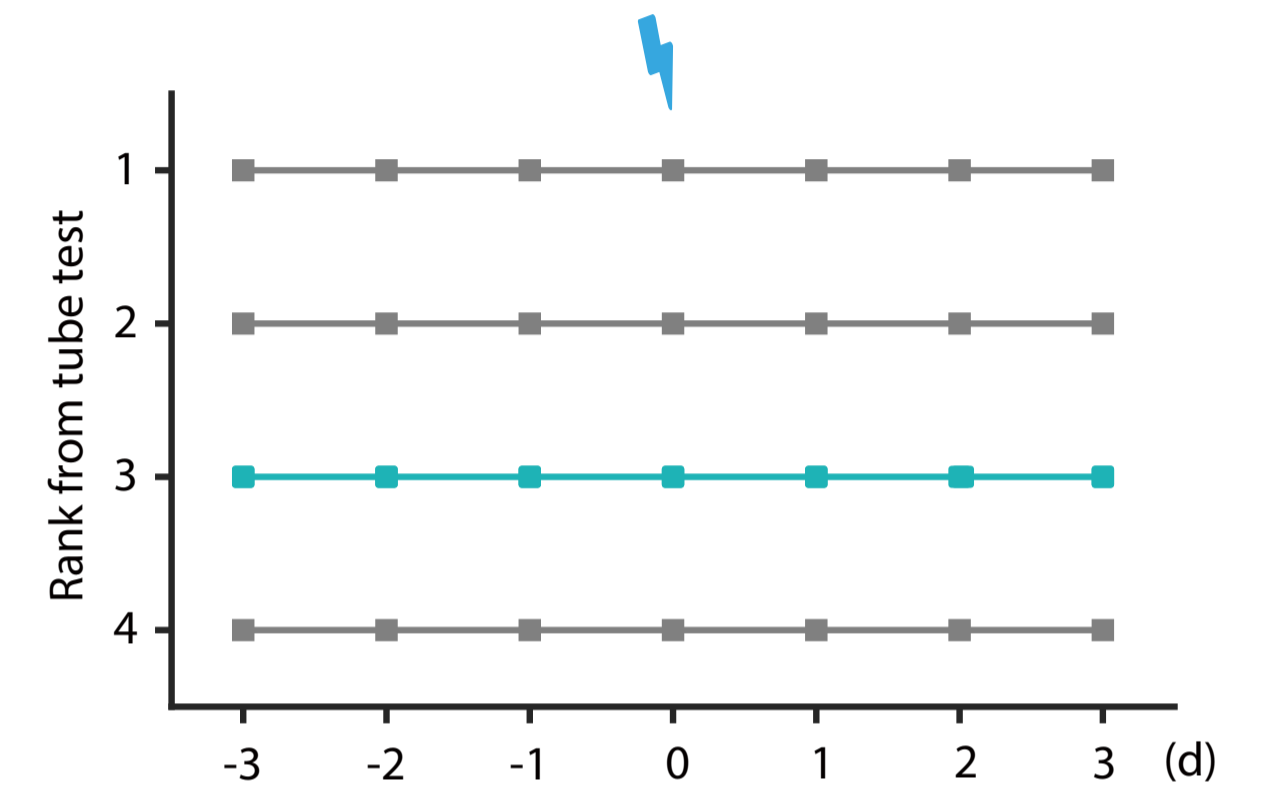

G

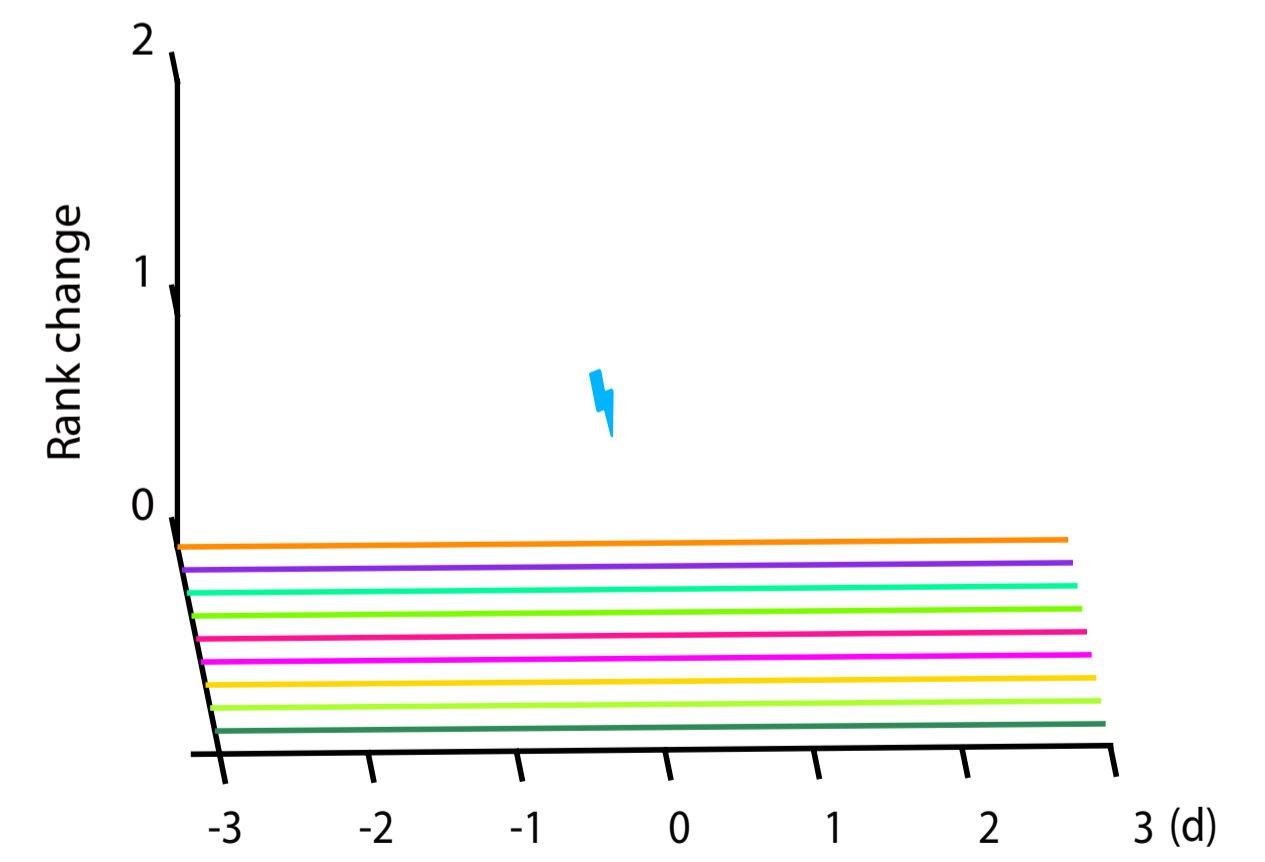

Supplement: S6 Fig — (A) Number and duration of push-initiation for each animal in the tube test (n = 8, Wilcoxon signed rank test; number: Z = −0.845, p = 0.398; duration: Z = 0.000, p = 1.000). (B) Number and duration of push-back for each animal in the tube test (n = 8, Wilcoxon signed rank test; number: Z = −0.141, p = 0.888; duration: Z = −1.400, p = 0.161). (C) Number and duration of stillness for each animal in the tube test (n = 8, Wilcoxon signed rank test; number: Z = −0.281, p = 0.779; duration: Z = −1.680, p = 0.093). (D) Percentage of time spent resisting (n = 8, Wilcoxon signed rank test; Z = −0.700, p = 0.484). (E) Percentage of time spent retreating (n = 8, Wilcoxon signed rank test; Z = −0.980, p = 0.327). (F) Example of rank positions for one cage of mice tested daily over 7 days, showing that the third-ranked mouse did not change its rank following photostimulation of SNrGlu neurons. (G) Summary of rank changes in EYFP mice before and after photostimulation on SNrGlu neurons (n = 9). Each line represents one animal. The data underlying this Figure can be found in file number 25 on Dryad (https://doi.org/10.5061/dryad.m0cfxppg3). (PDF) [file pbio.3003687.s006.pdf]

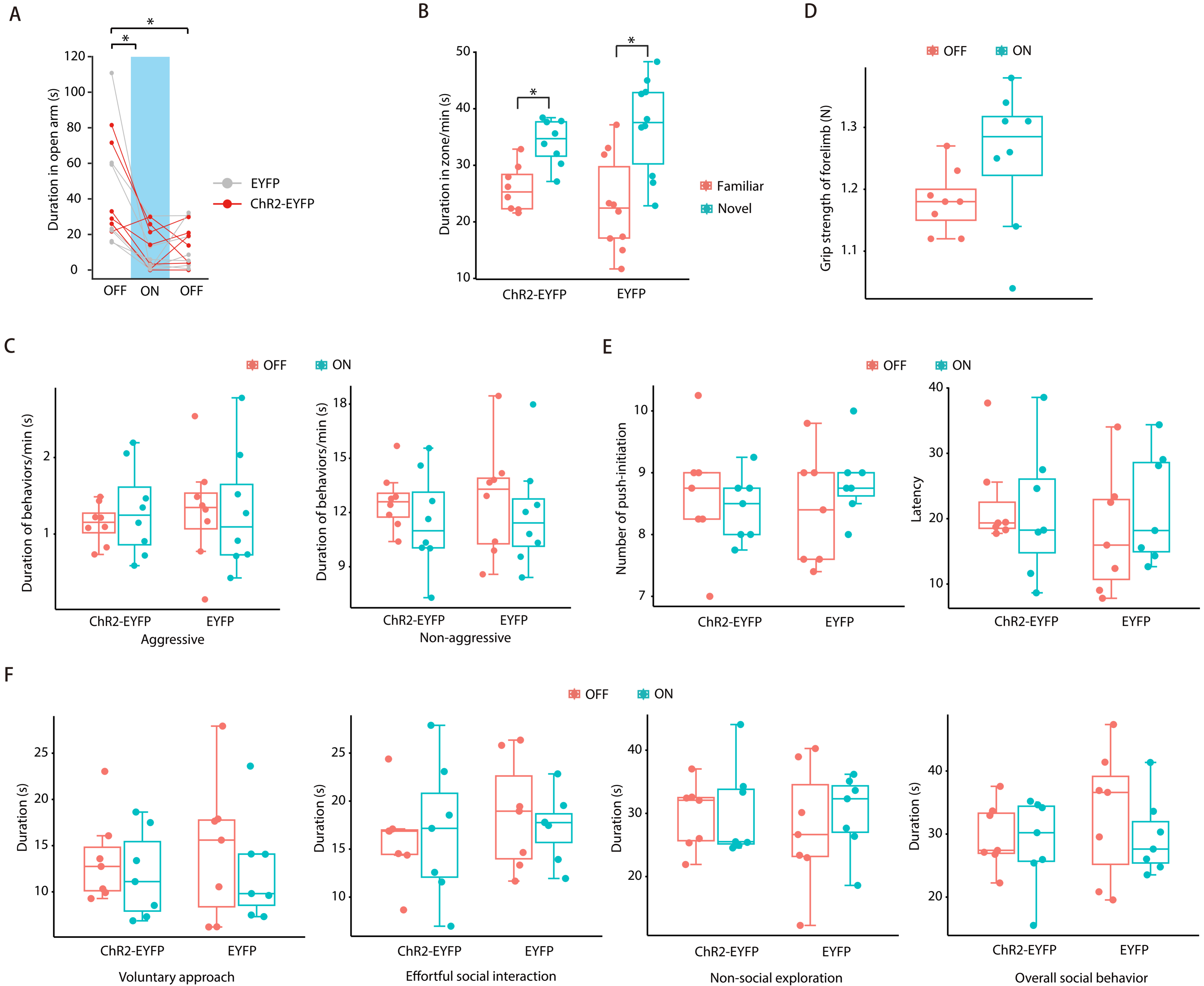

Supplement: S7 Fig — (A) During the elevated plus-maze (EPM) test, time spent in the open arm decreased over time (n = 6 for ChR2-EYFP mice and n = 7 for EYFP mice, two-way repeated measures ANOVA with Bonferroni correction; F2,22 = 15.814, p < 0.001), though no significant differences were observed between groups (F1,11 = 0.621, p = 0.447). (B) In the social memory test, both ChR2-EYFP and EYFP mice showed a significant preference for novel mice during light-on period (n = 8 and 10 for ChR2-EYFP and EYFP mice, respectively; two-way repeated measures ANOVA with Bonferroni correction; F1,16 = 11.293, p = 0.004). However, no significant differences were found between groups (F1,16 = 4.127, p = 0.059). (C) The resident-intruder test indicated that both ChR2-EYFP and EYFP mice displayed normal levels of aggressive and nonaggressive behaviors towards novel mice (n = 8 for each group, two-way repeated measures ANOVA with Bonferroni correction; F1,14 = 0.146, p = 0.708), with no significant differences between groups (F1,14 = 0.137, p = 0.716). (D) Optogenetic activation of SNrGlu neurons did not affect forelimb grip strength during light-on and light-off periods (n = 8, paired two-sided t test; t7 = 1.735, p = 0.126). (E) In the push ball test, the analysis of latency revealed no significant main effects for “light condition“ (n = 7 for each group; two-way repeated measures ANOVA with Bonferroni correction; F₁,₁₂ = 0.131, p = 0.723) or “group” (F₁,₁₂ = 0.336, p = 0.573). Similarly, the assessment of the number of active pushes showed no significant main effects for “light condition” (F₁,₁₂ = 0.220, p = 0.647) or “group” (F₁,₁₂ = 0.079, p = 0.783). (F) In the social interaction test, the duration analysis indicated no significant main effects for “light condition” (n = 7 for each group; two-way repeated measures ANOVA with Bonferroni correction): voluntary approach (F₁,₁₂ = 1.700, p = 0.217), effortful social interaction (F₁,₁₂ = 0.017, p = 0.897), overall social behavior (F₁,₁₂ = 0.696, p = 0 [file pbio.3003687.s007.pdf]

A

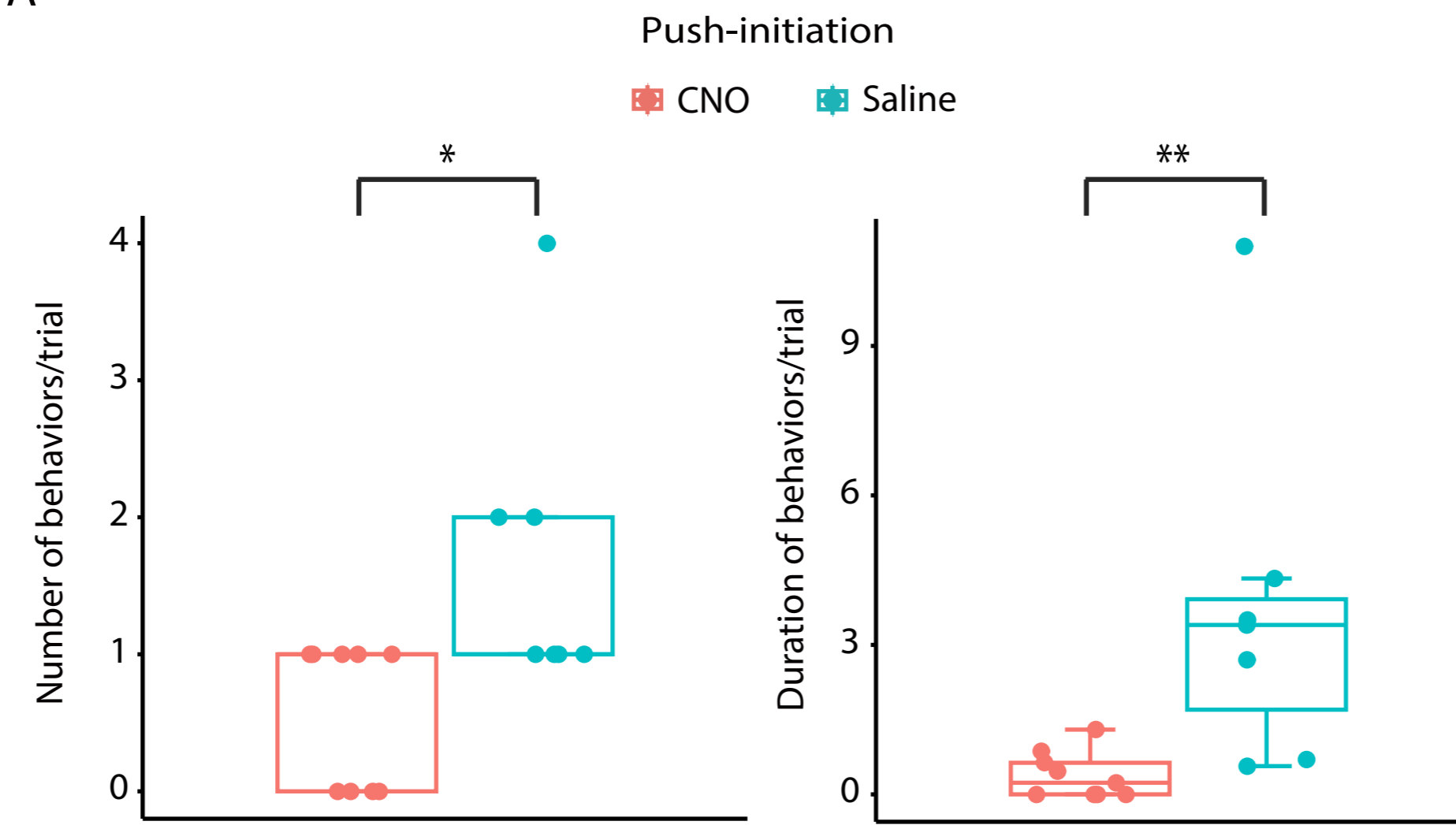

B

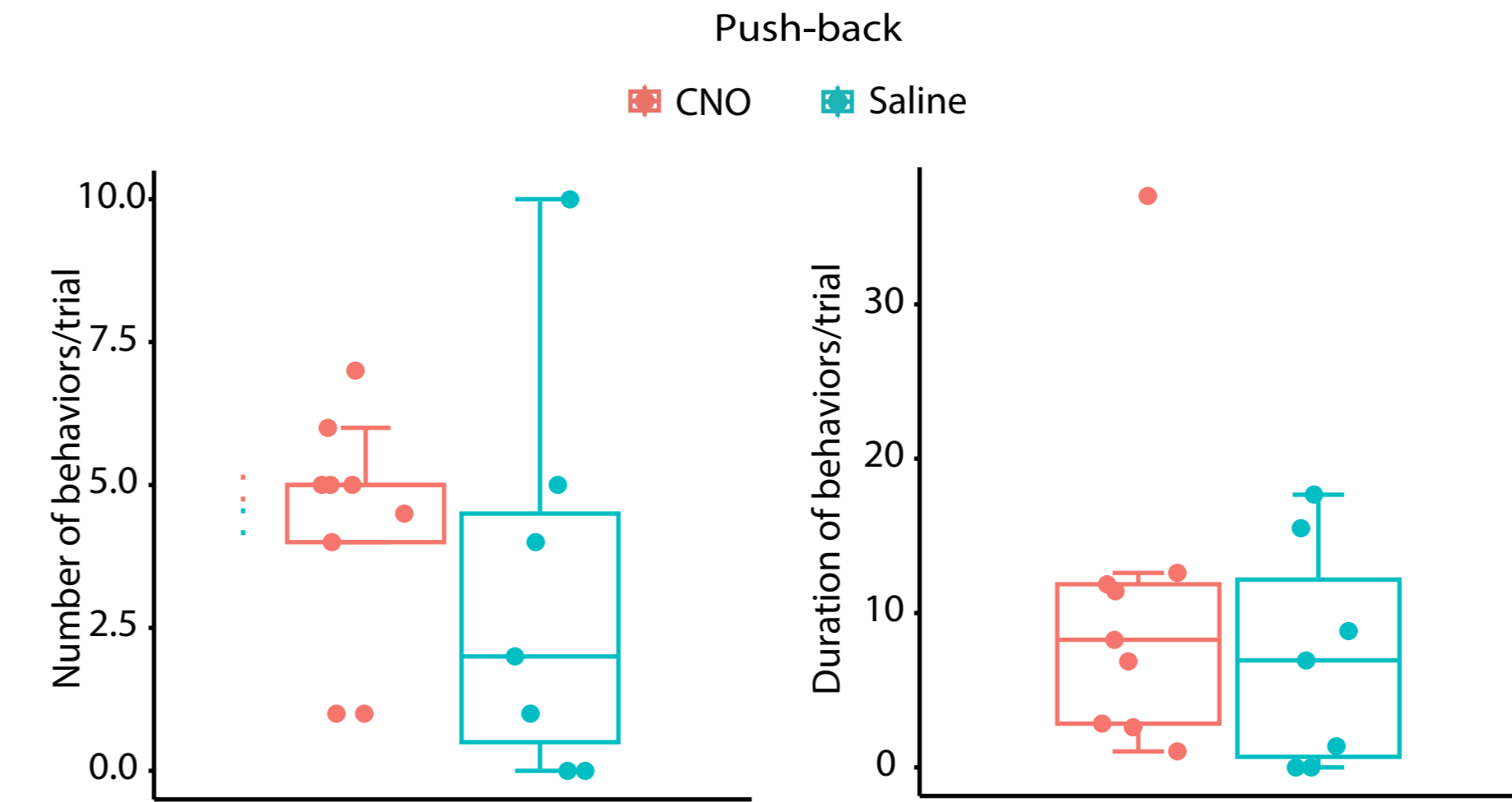

C

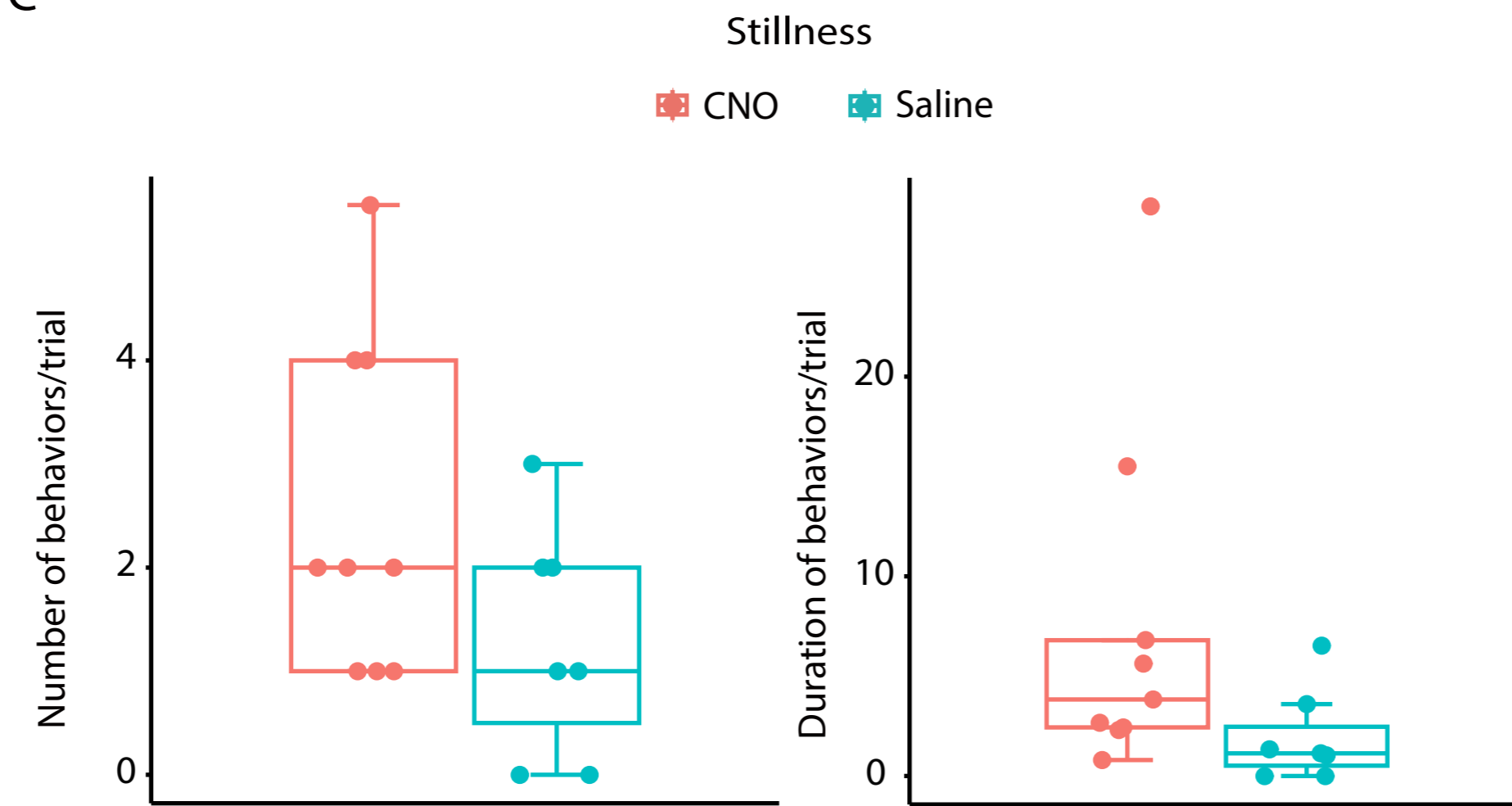

D

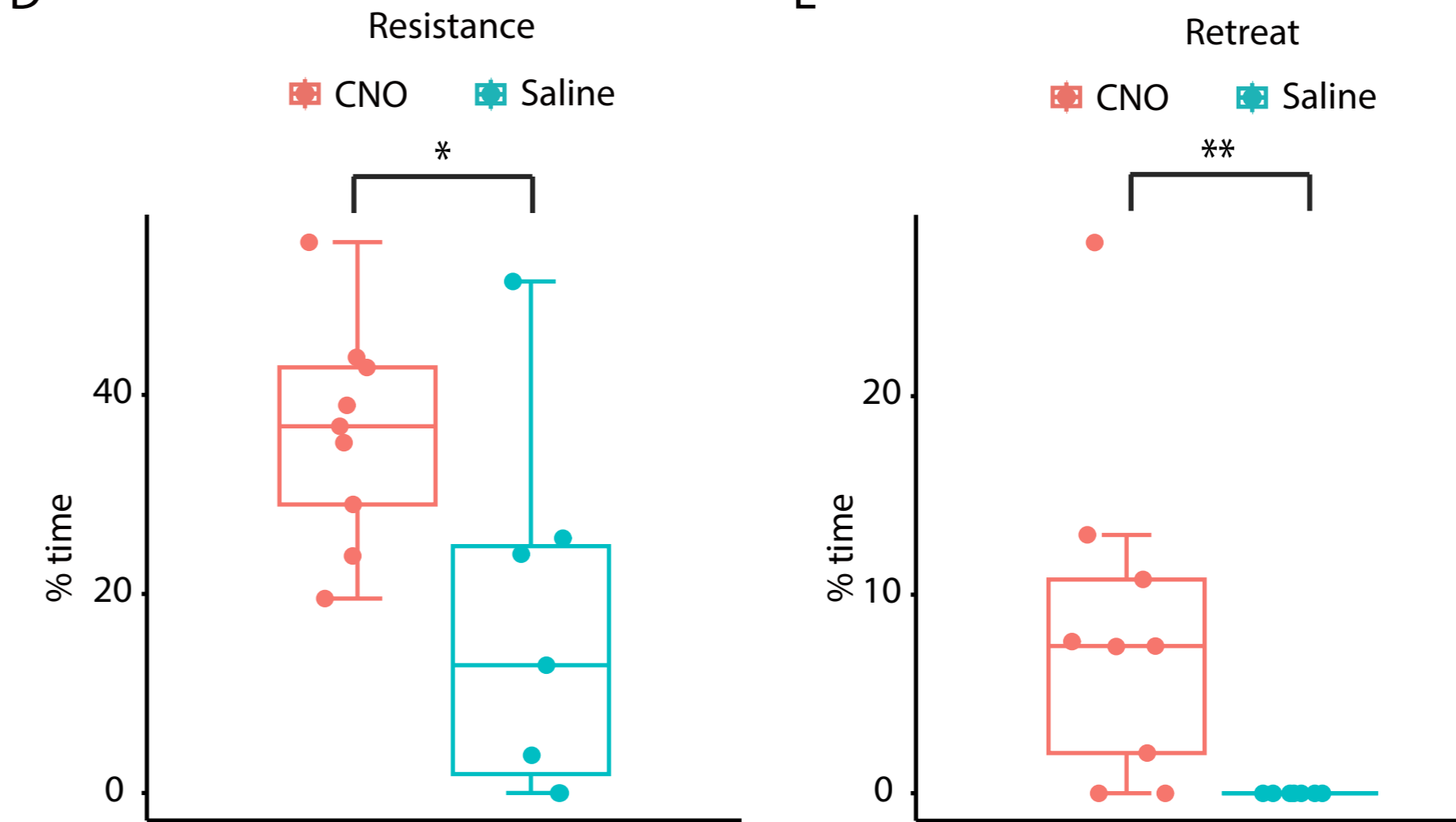

Supplement: S8 Fig — (A) Number and duration of push-initiation for each animal in the tube test (n = 7 mice for saline injection and n = 9 mice for CNO injection, Mann–Whitney U test; number: U = 10, p = 0.011; duration: U = 5, p = 0.005). (B) Number and duration of push-back for each animal in the tube test (n = 7 versus 9, Mann–Whitney U test; number: U = 20, p = 0.218; duration: U = 26, p = 0.560). (C) Number and duration of stillness for each animal in the tube test (n = 7 versus 9, Mann–Whitney U test; number: U = 18, p = 0.140; duration: U = 13, p = 0.050). (D) Percentage of time spent resisting (n = 7 versus 9, Mann–Whitney U test; U = 12, p = 0.039). (E) Percentage of time spent retreating (n = 7 versus 9, Mann–Whitney U test; U = 7, p = 0.004). Note that the same 3 animals were used in both groups; specifically, 3 out of 9 mice received i.p. injection of saline 1 week prior to the CNO injection. * p < 0.05; ** p < 0.01. The data underlying this Figure can be found in file number 36 on Dryad (https://doi.org/10.5061/dryad.m0cfxppg3). (PDF) [file pbio.3003687.s008.pdf]

A

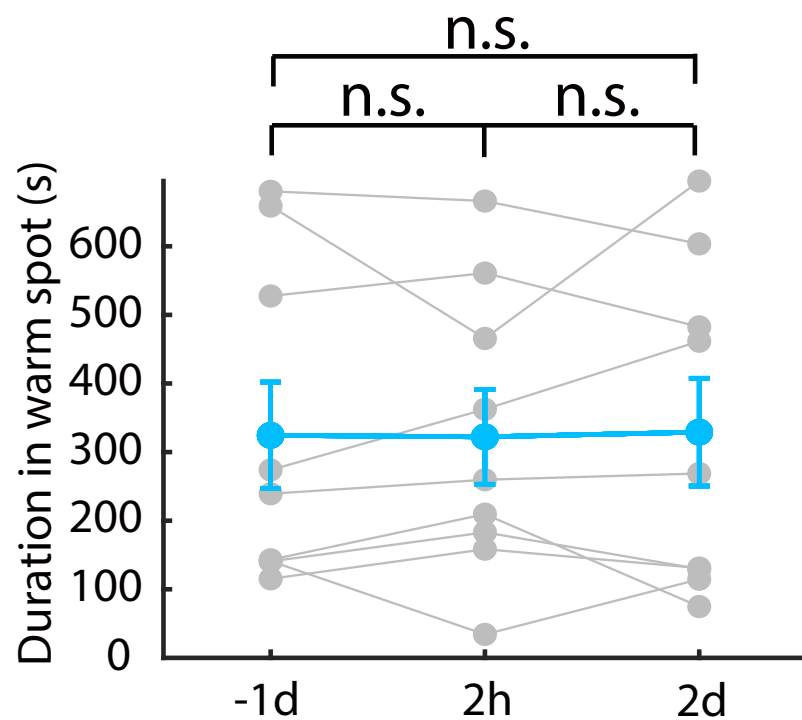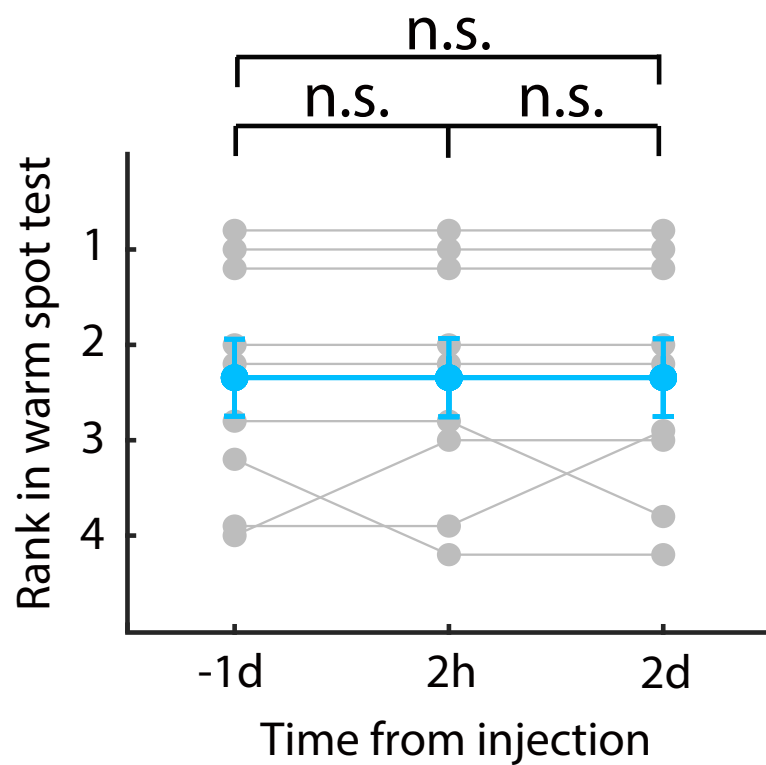

B

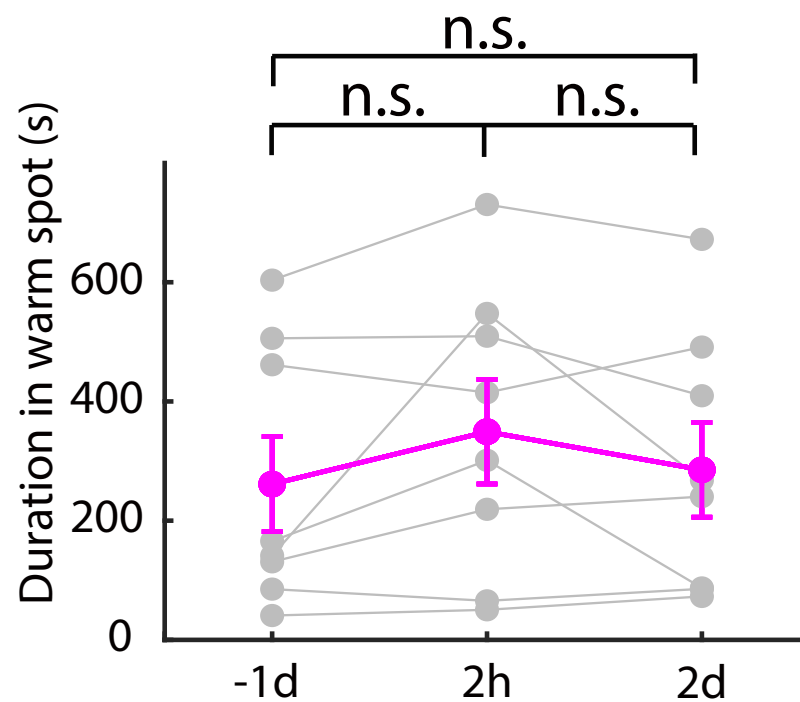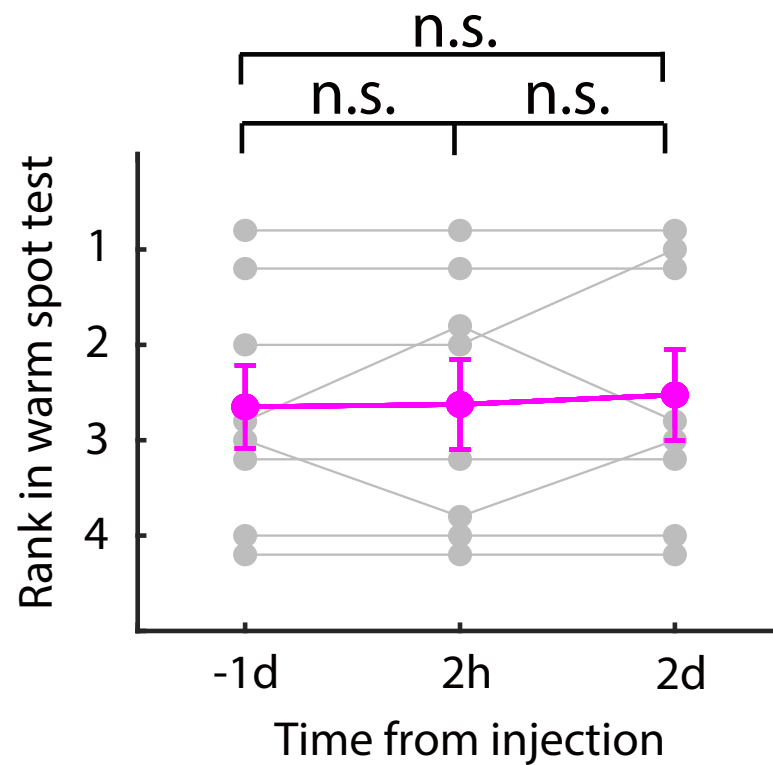

Supplement: S9 Fig — (A) The duration (top) and rank (bottom) in the warm spot test were assessed for mice expressing EYFP in the SNr at 1 day before, 2 hours post, and 2 days post i.p. administration of saline (duration in the warm spot: n = 9, one-way repeated measures ANOVA with Bonferroni correction, F2,16 = 0.007; p = 0.993; rank: Friedman test, t2 = 0.000; p = 1.000). (B) Similarly, the duration (top) and rank (bottom) in the warm spot test for mice expressing EYFP in the SNr at 1 day before, 2 hours after, and 2 days following i.p. injection of CNO (duration: n = 8, one-way repeated measures ANOVA with Bonferroni correction, F2,14 = 2.273; p = 0.140; rank: Friedman test, t2 = 0.667; p = 0.717). Notably, all mice received an i.p. injection of saline 1 week prior to the CNO injection. Each line on the graph represents an individual animal. The data underlying this Figure can be found in files numbered 37–38 on Dryad (https://doi.org/10.5061/dryad.m0cfxppg3). (PDF) [file pbio.3003687.s009.pdf]

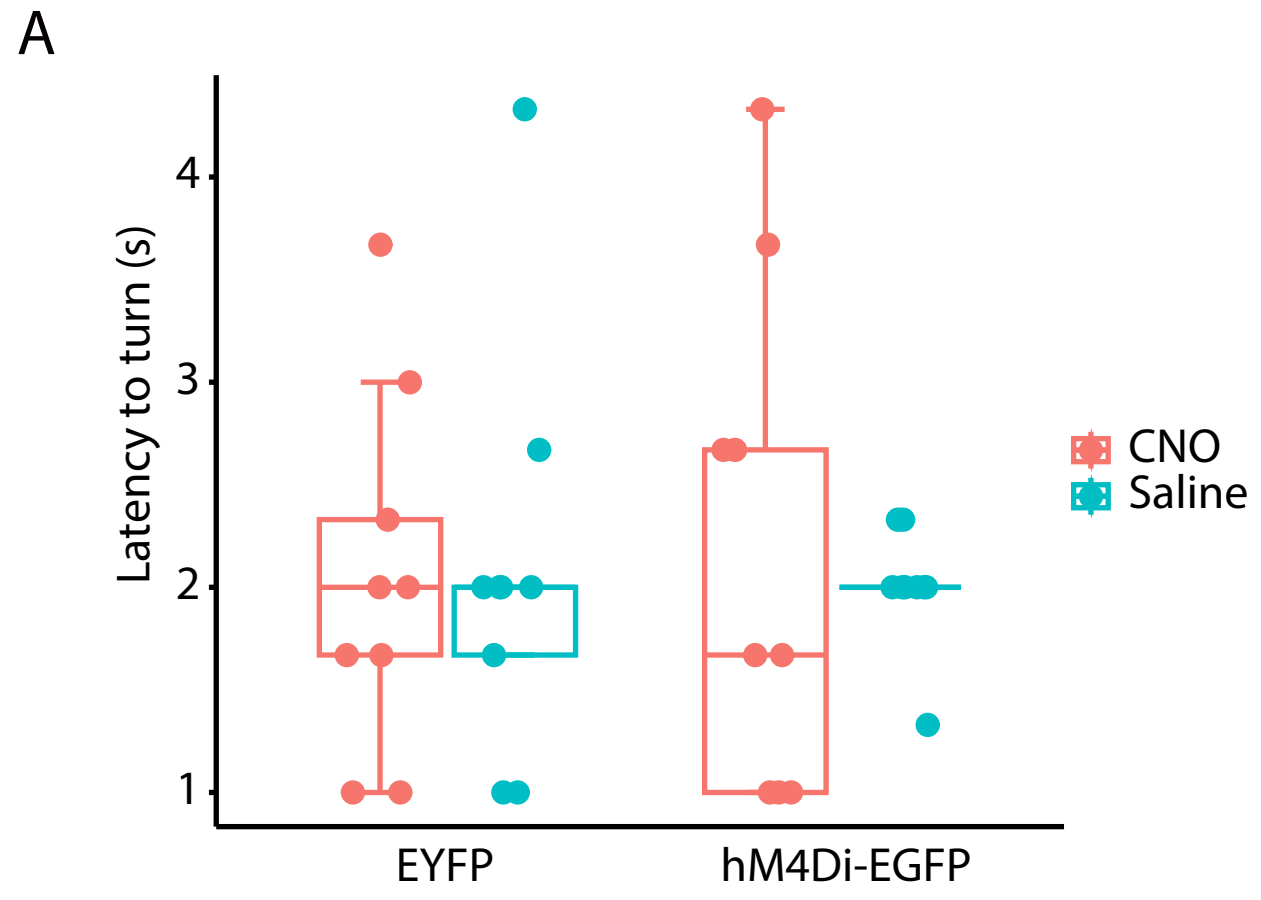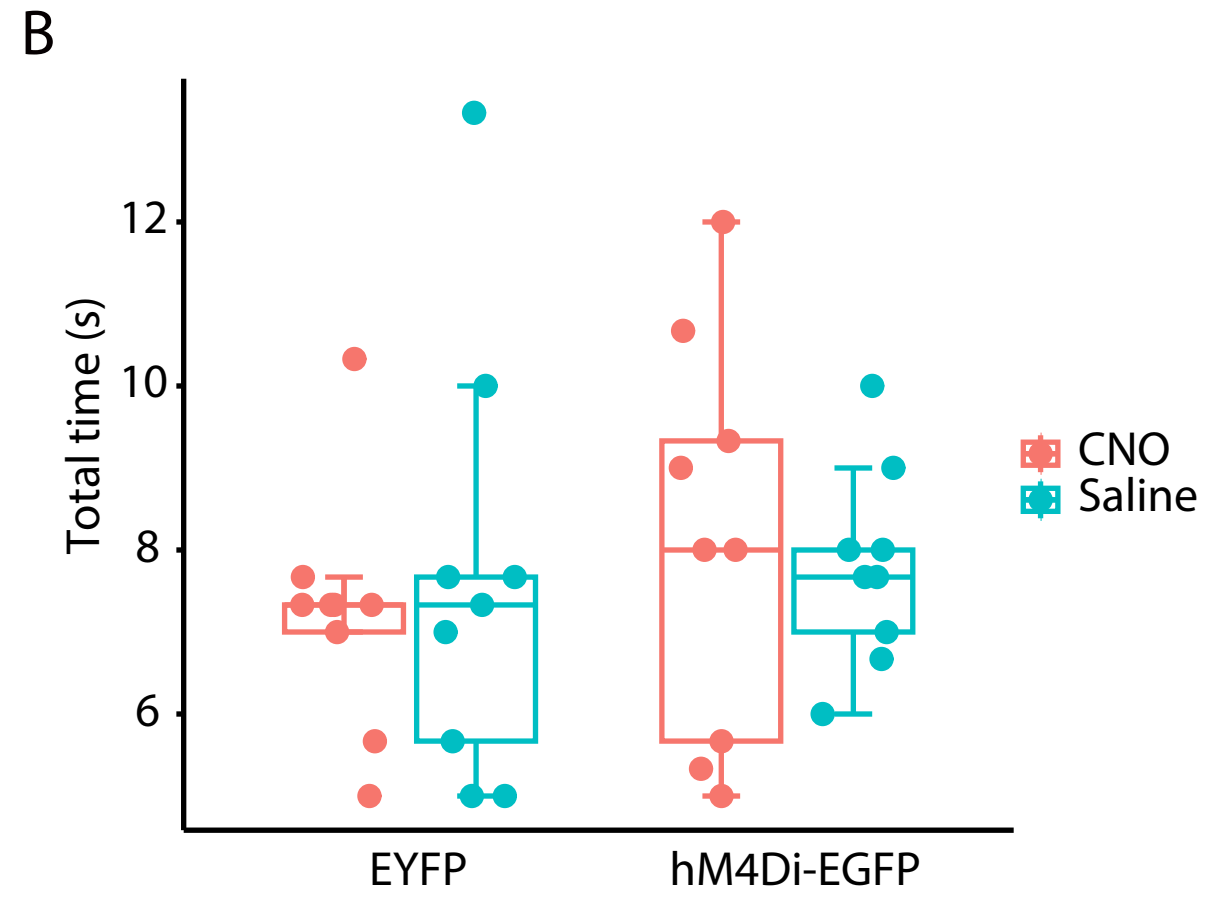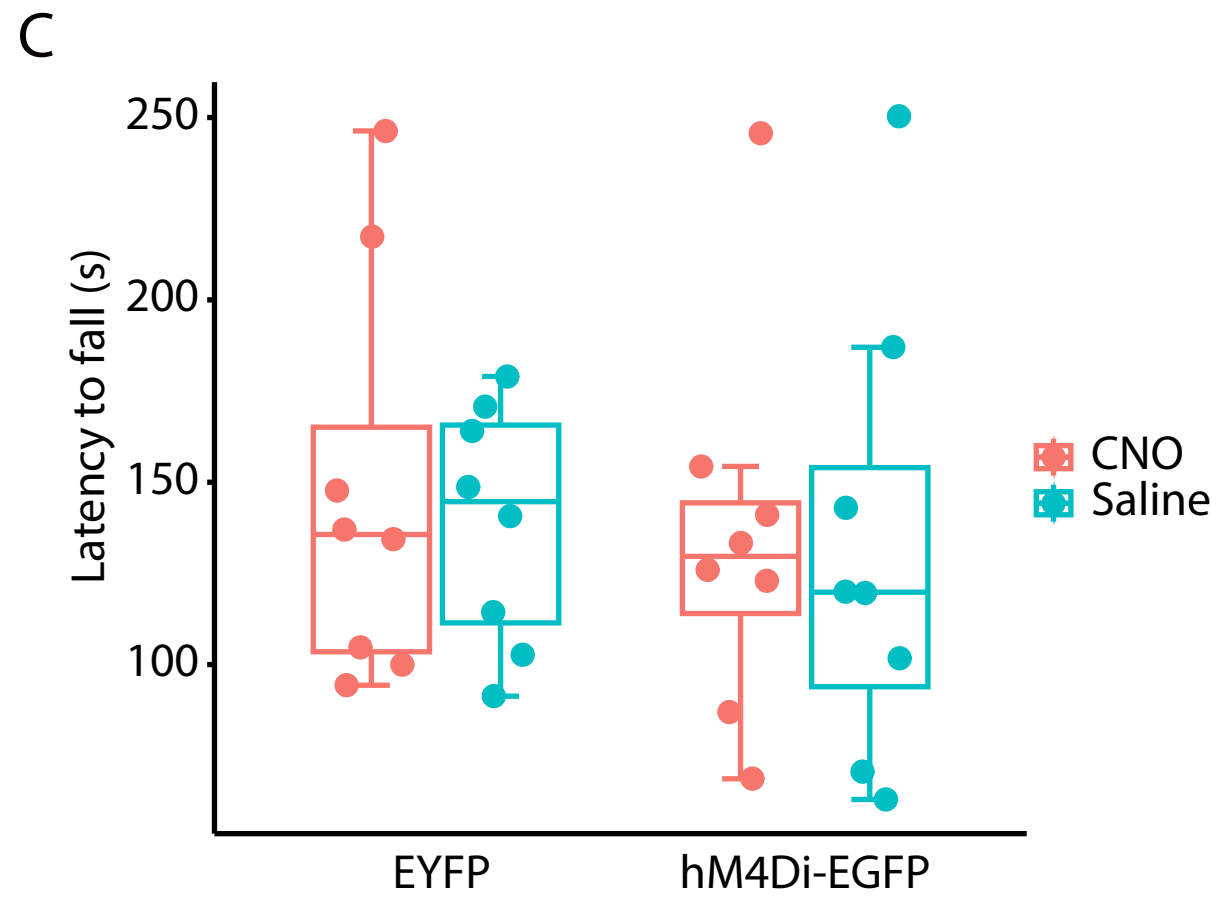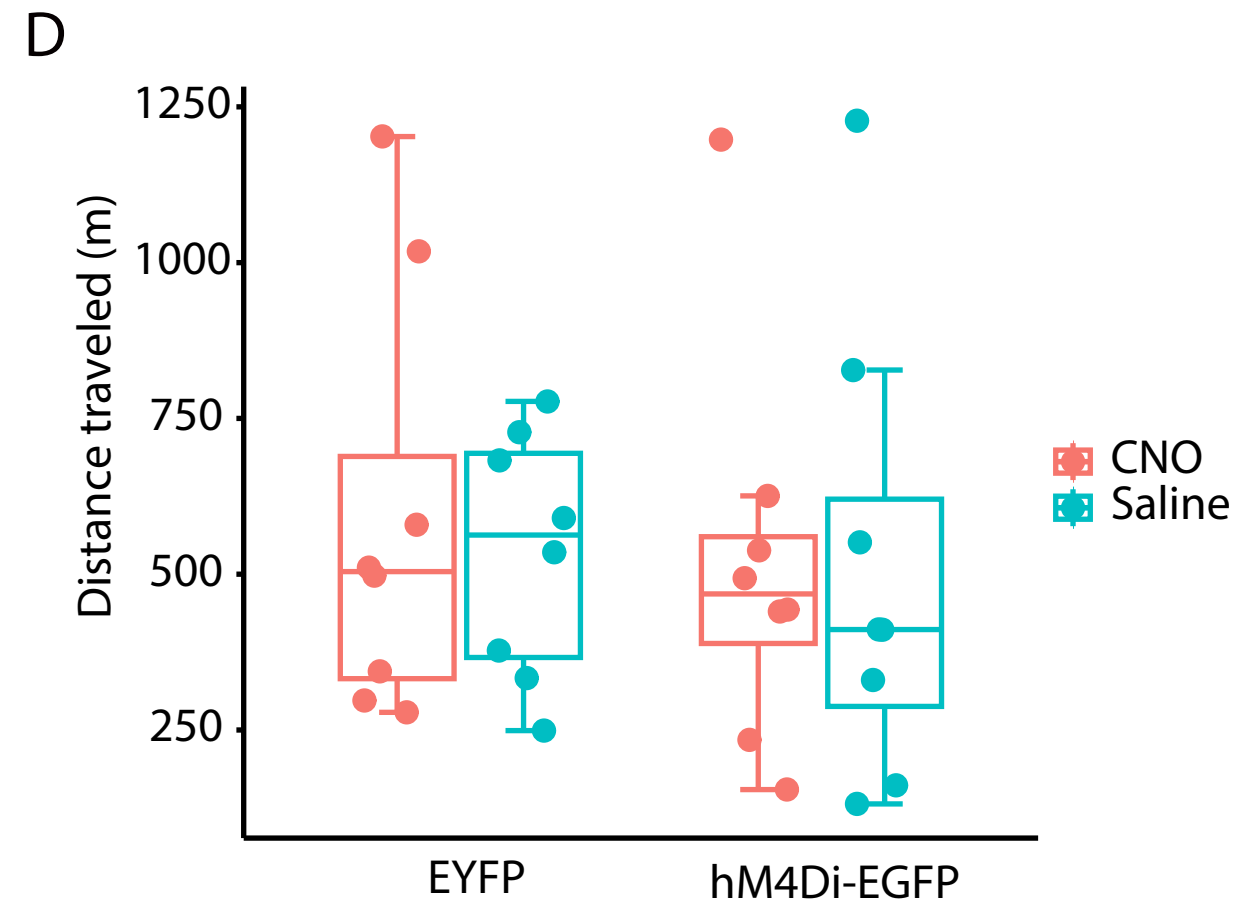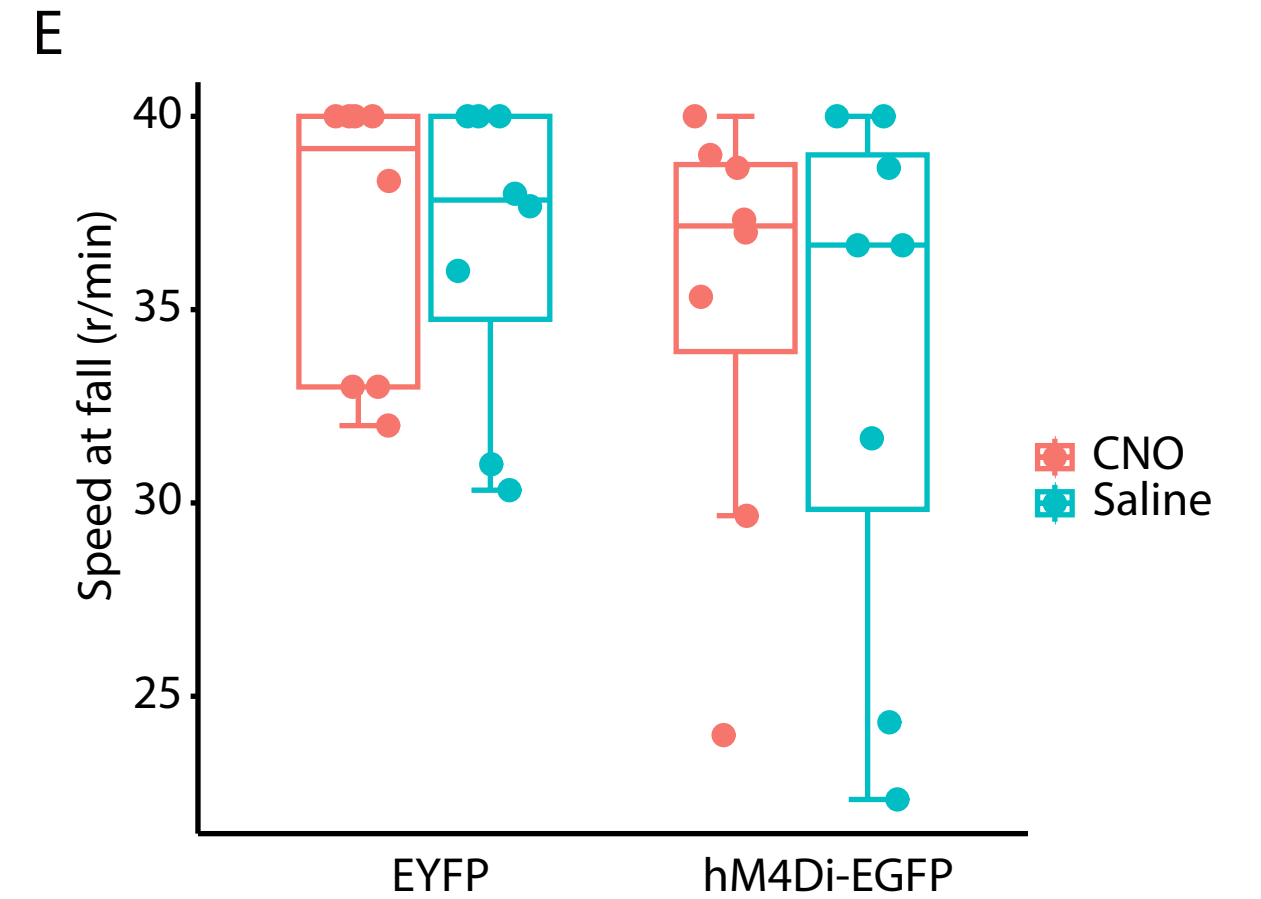

Supplement: S10 Fig — (A) There was no significant difference in the duration required for mice to transition from the head-up to head-down position between CNO and saline injections (n = 9 for both hM4Di-EGFP and EYFP groups; two-way repeated measures ANOVA with Bonferroni correction; F1,16 = 0.067, p = 0.799) or between hM4Di-EGFP and EYFP mice (F1,16 = 0.013, p = 0.910). (B) The total duration from head-up to descent at the bottom of the pole did not significantly differ between CNO and saline injections (F1,16 = 0.007, p = 0.936) or between hM4Di-EGFP and EYFP mice (F1,16 = 0.377, p = 0.548). (C) No significant difference in latency to fall was observed between CNO and saline injections (n = 8 for both hM4Di-EGFP and EYFP groups; two-way repeated measures ANOVA with Bonferroni correction; F1,14 = 0.163, p = 0.692) or between hM4Di-EGFP and EYFP mice (F1,14 = 0.211, p = 0.653). (D) Distance traveled did not differ significantly between CNO and saline injections (F1,14 = 0.137, p = 0.717) or between hM4Di-EGFP and EYFP mice (F1,14 = 0.163, p = 0.693). (E) There was also no significant difference in speed at the moment of fall between CNO and saline injections (F1,14 = 0.705, p = 0.415) or between hM4Di-EGFP and EYFP mice (F1,14 = 0.990, p = 0.337). The data underlying this Figure can be found in files numbered 39–43 on Dryad (https://doi.org/10.5061/dryad.m0cfxppg3). (PDF) [file pbio.3003687.s010.pdf]

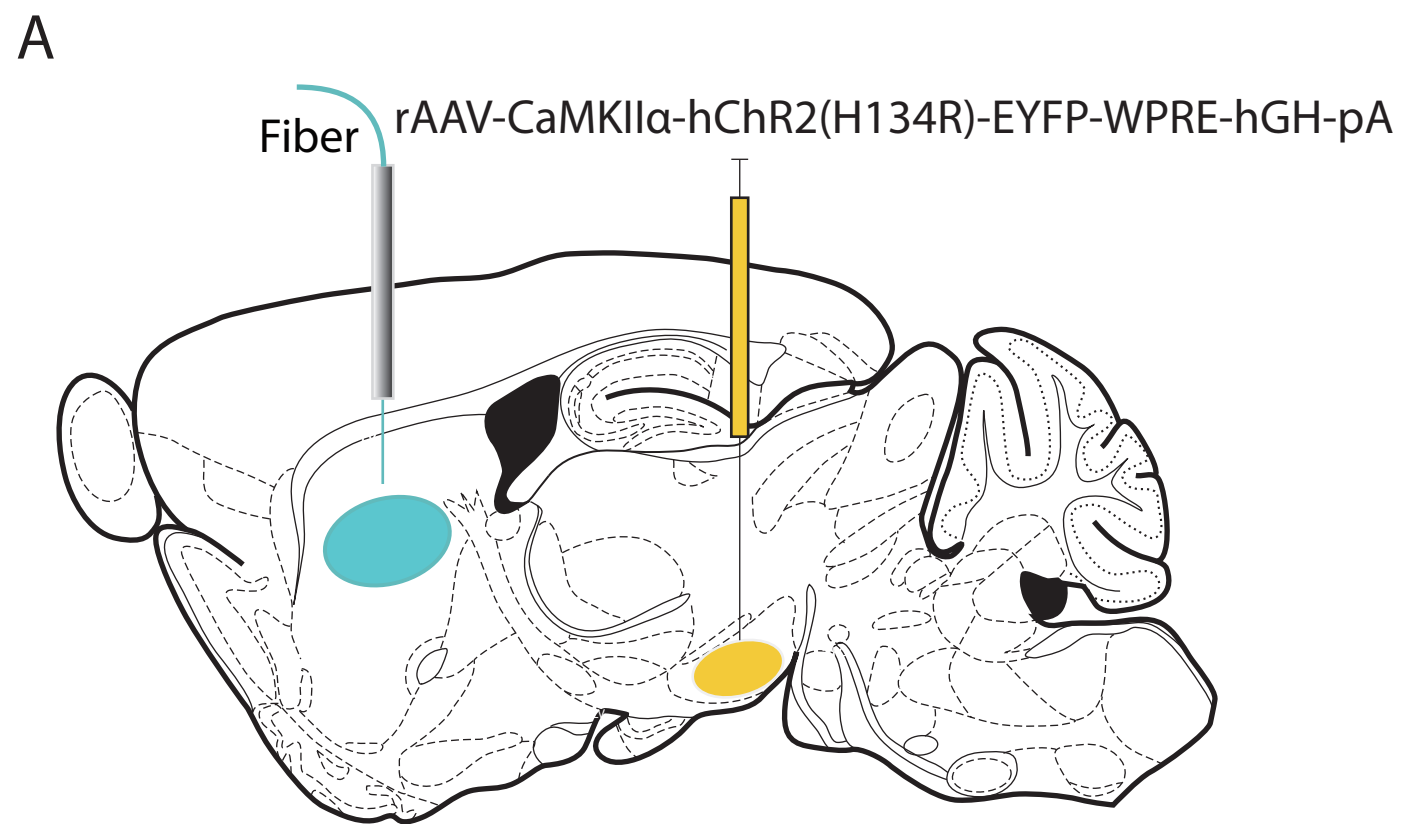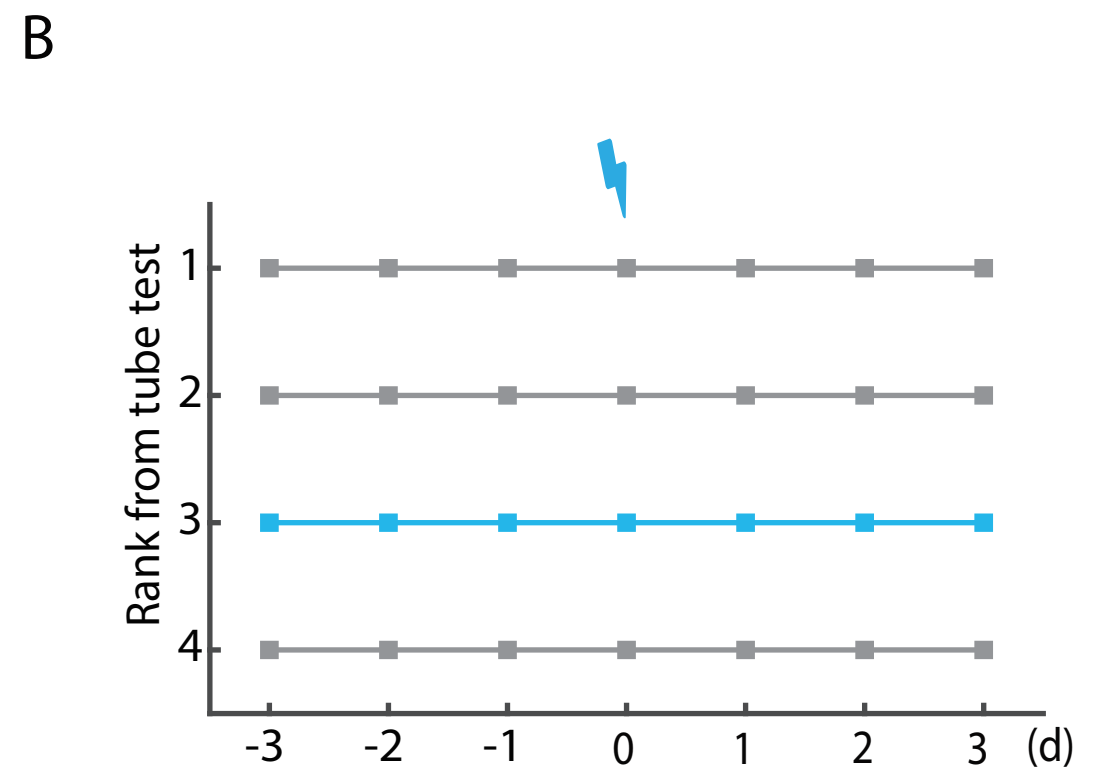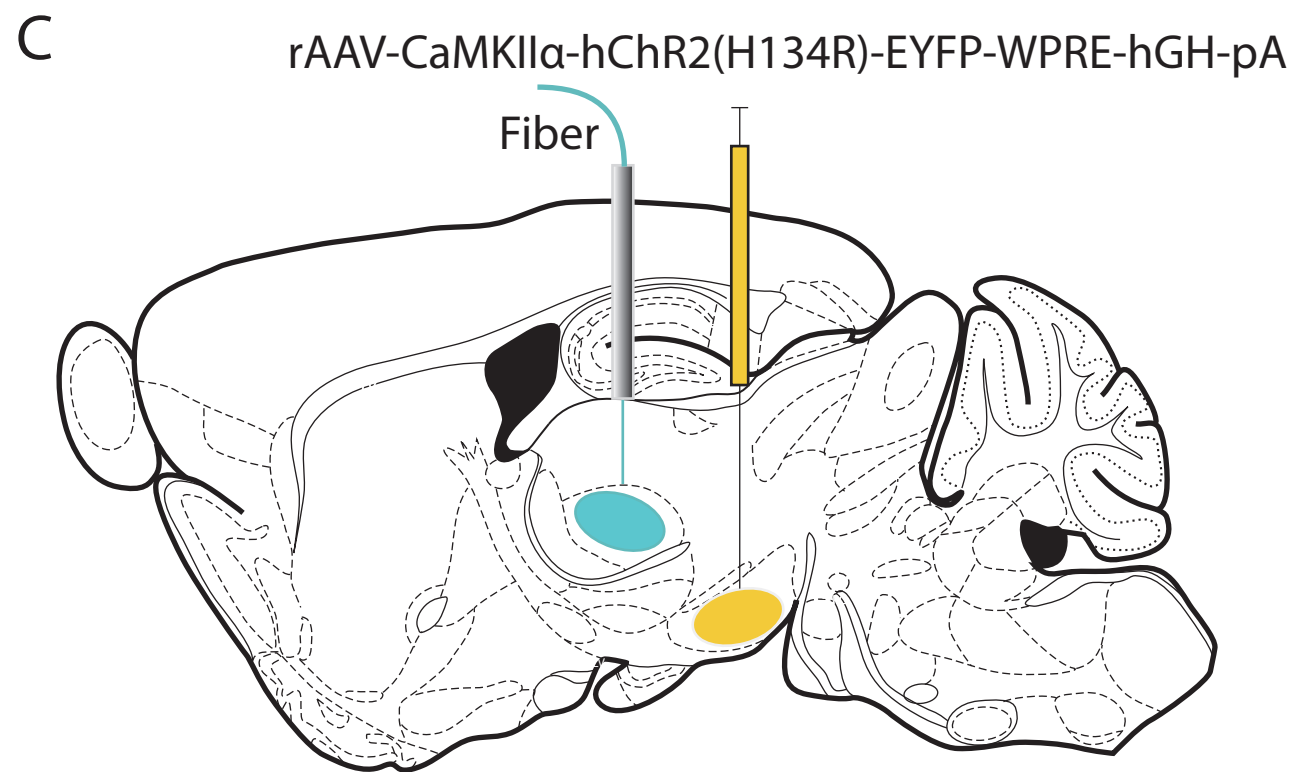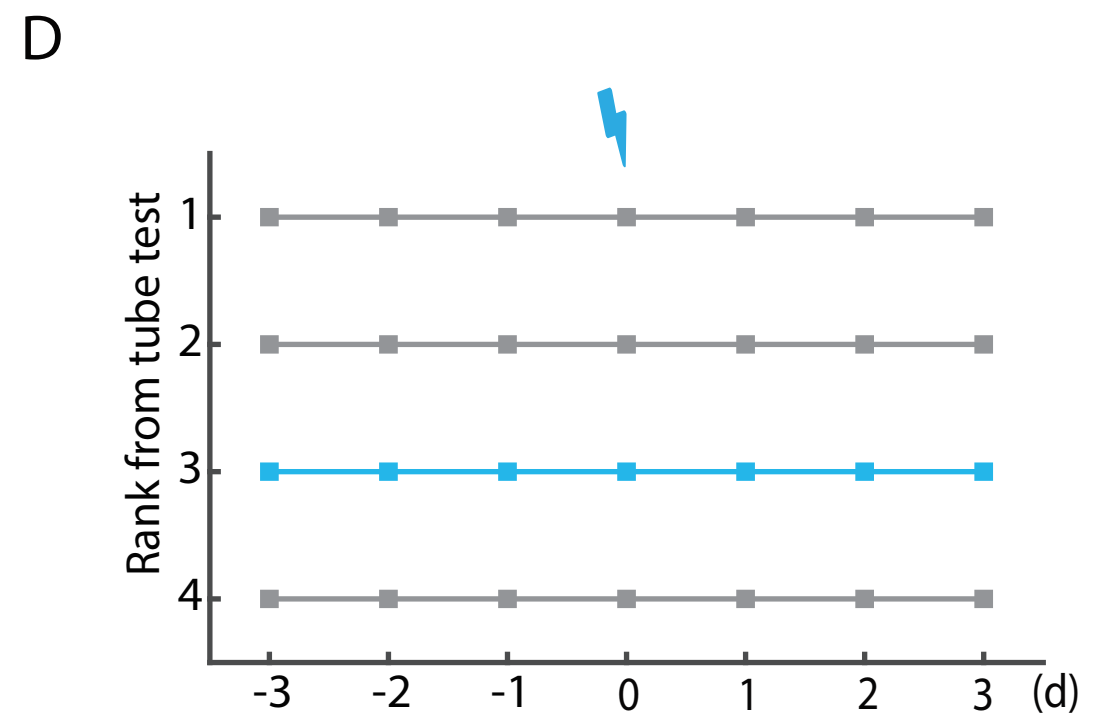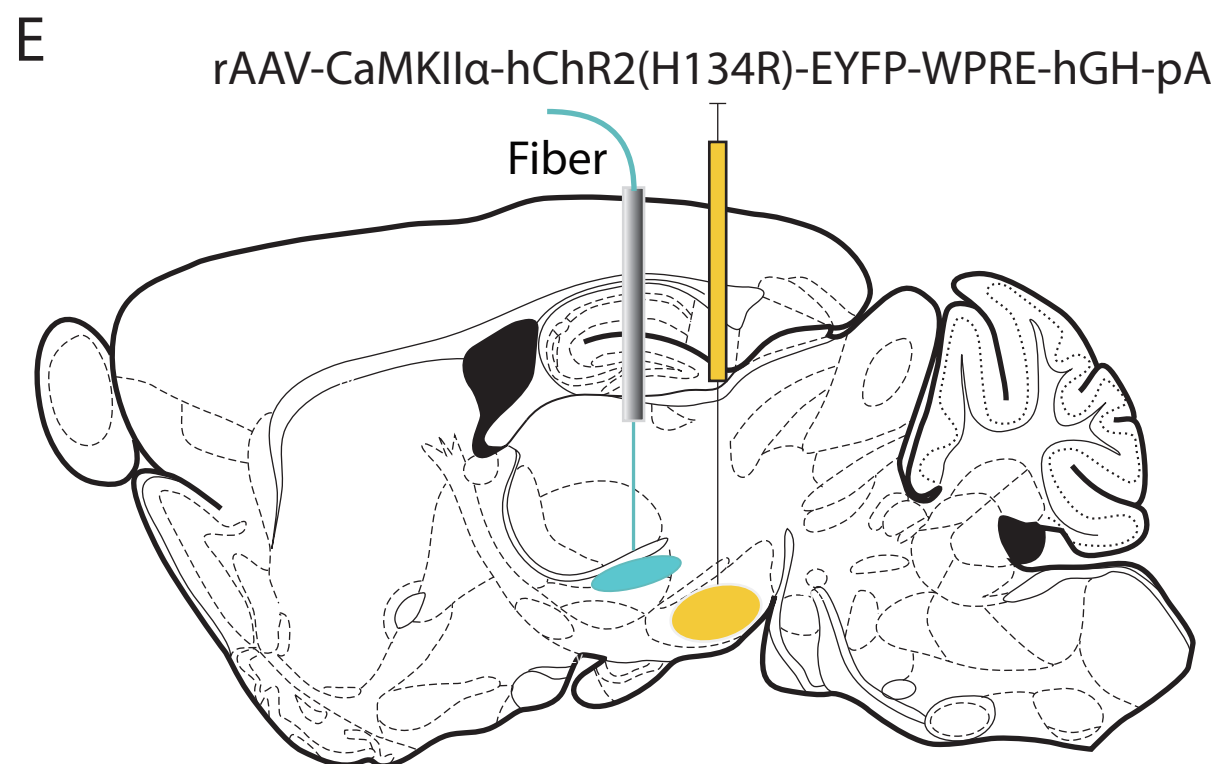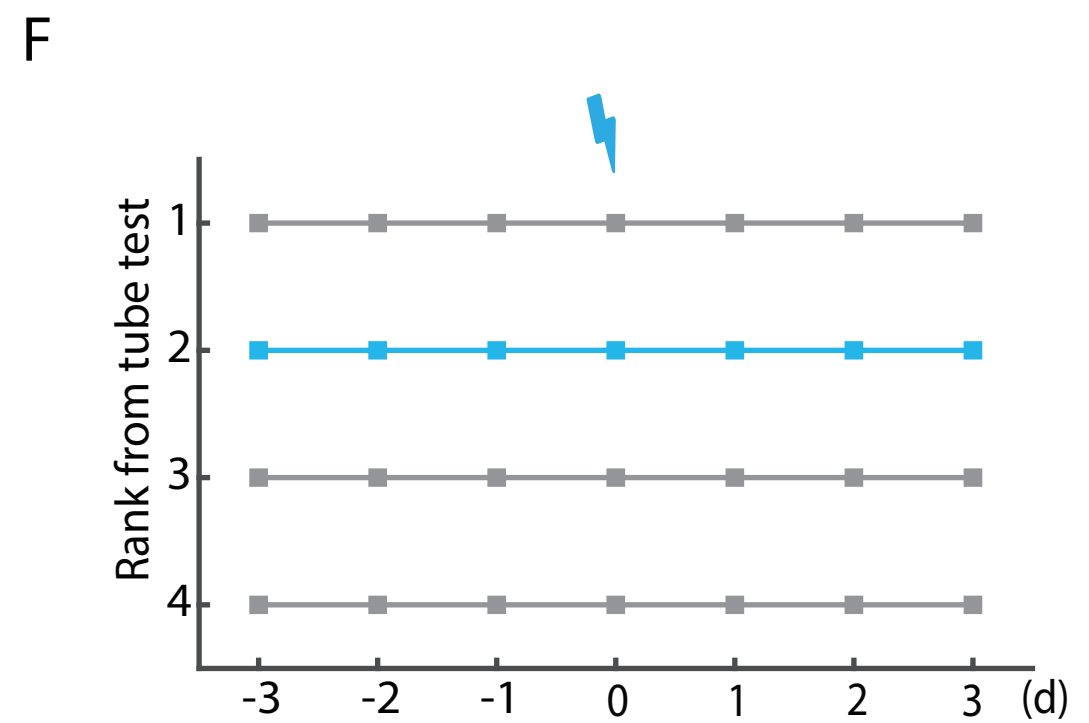

Supplement: S11 Fig — Panels (A), (C), and (E) illustrate diagrams of the unilateral viral infection area using rAAV-CaMKIIα-hChR2(H134R)-EYFP-WPRE-hGH-pA in the SNr, along with the placement of optic fibers above the CPu (A), VPM (C), and ZID (E), respectively. Panels (B), (D), and (F) present examples of rank positions for a group of mice assessed daily over 7 days, showing that the rank of the third-position mouse remained unchanged following photostimulation of SNrGlu neuronal terminals in the CPu (B), VPM (D), and ZID (F), respectively. Each line represents an individual animal. Abbreviations: CPu, caudate putamen (striatum); VPM, ventral posteromedial thalamic nucleus; ZID, zona incerta, dorsal part. The stereotaxic coordinates (in mm) are: CPu (AP = 0.5, ML = ± 2.0, DV = −3.0), VPM (AP = −1.0, ML = ± 0.8, DV = −3.5), and ZID (AP = −1.8, ML = ± 1.0, DV = −4.0). (PDF) [file pbio.3003687.s011.pdf]

A

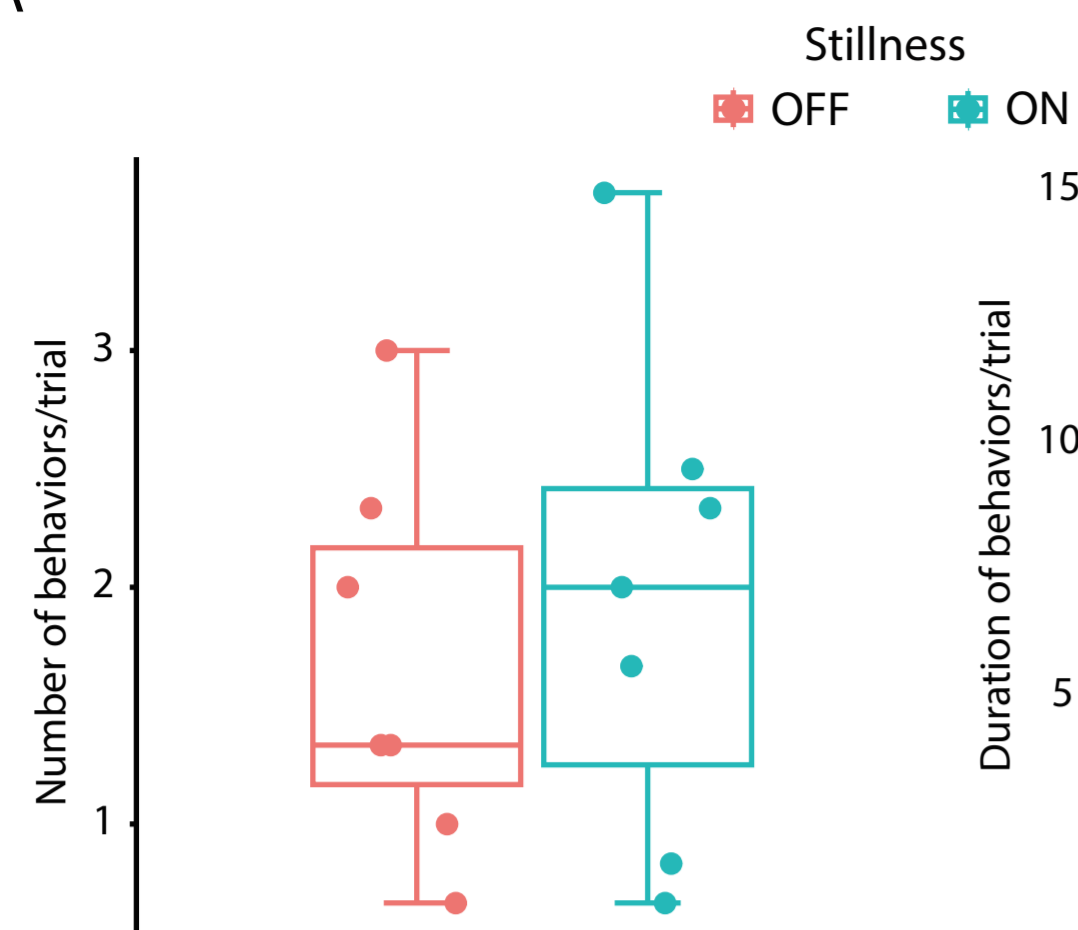

B

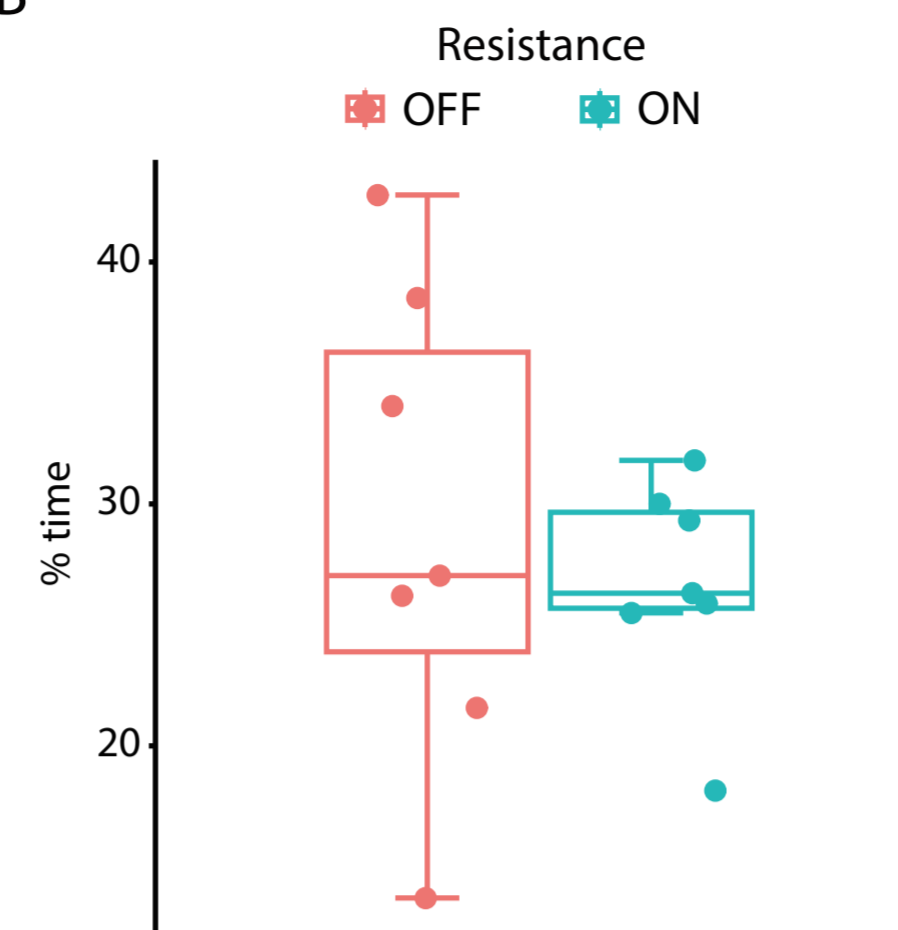

C

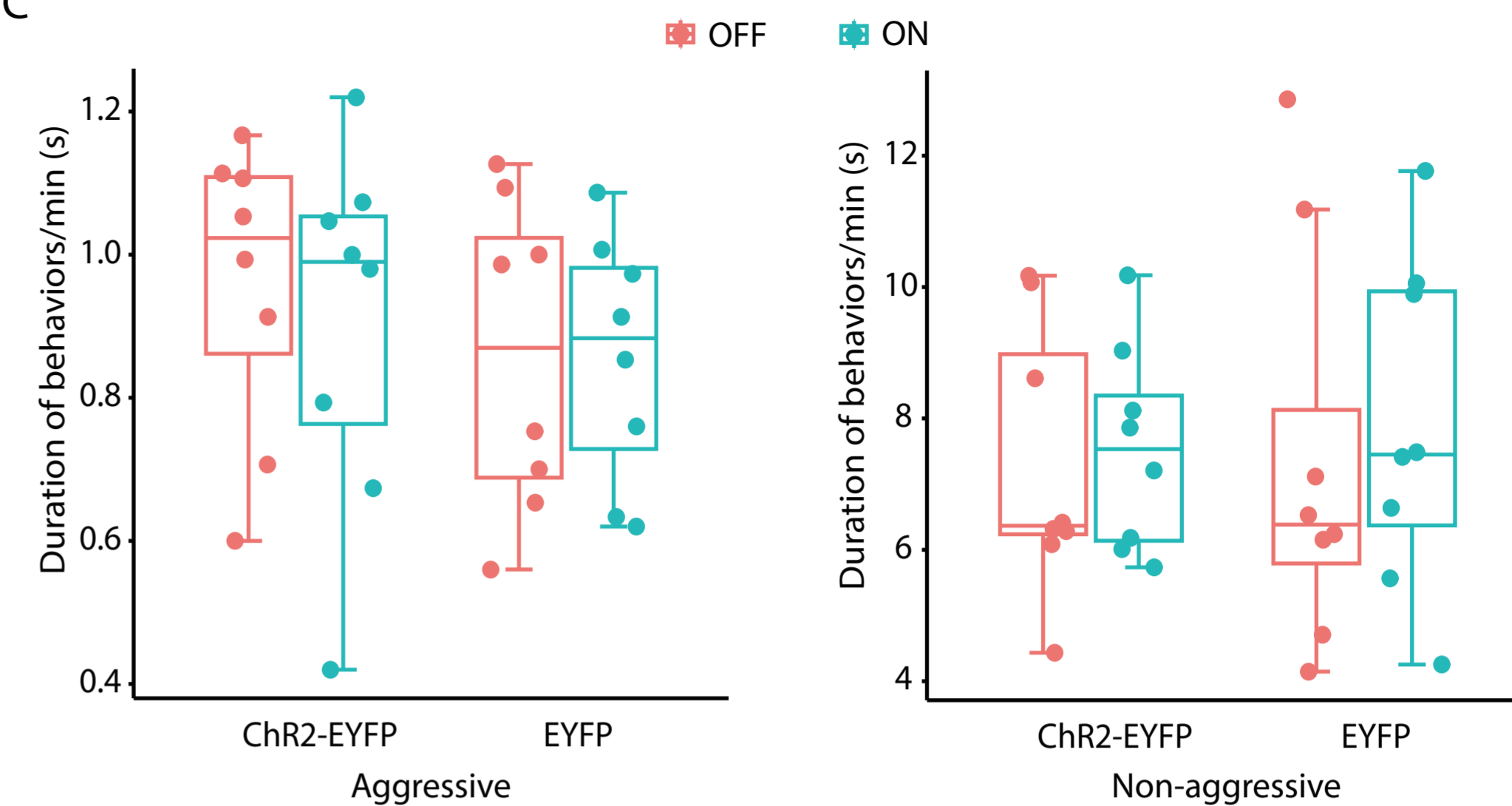

D

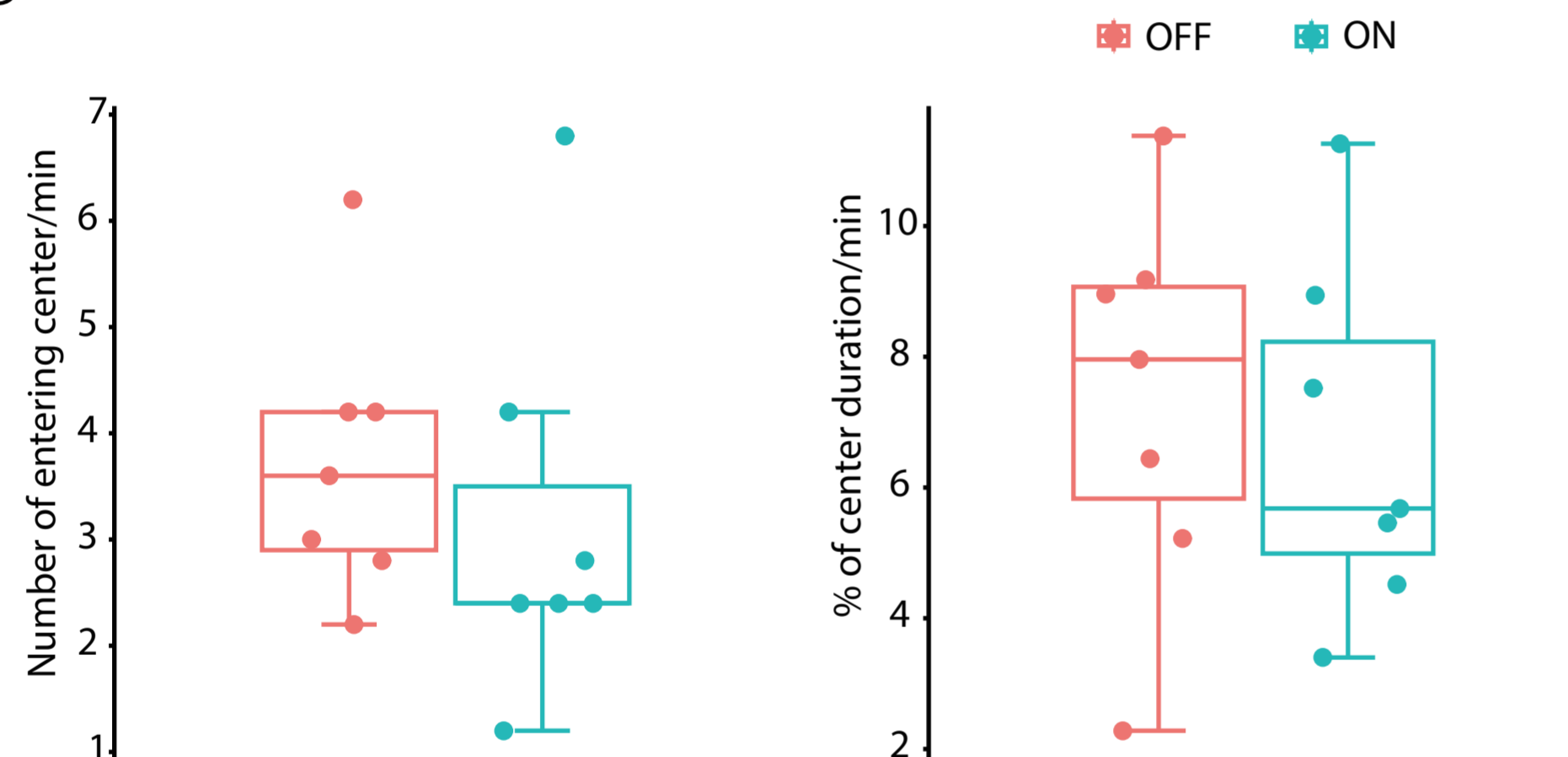

F

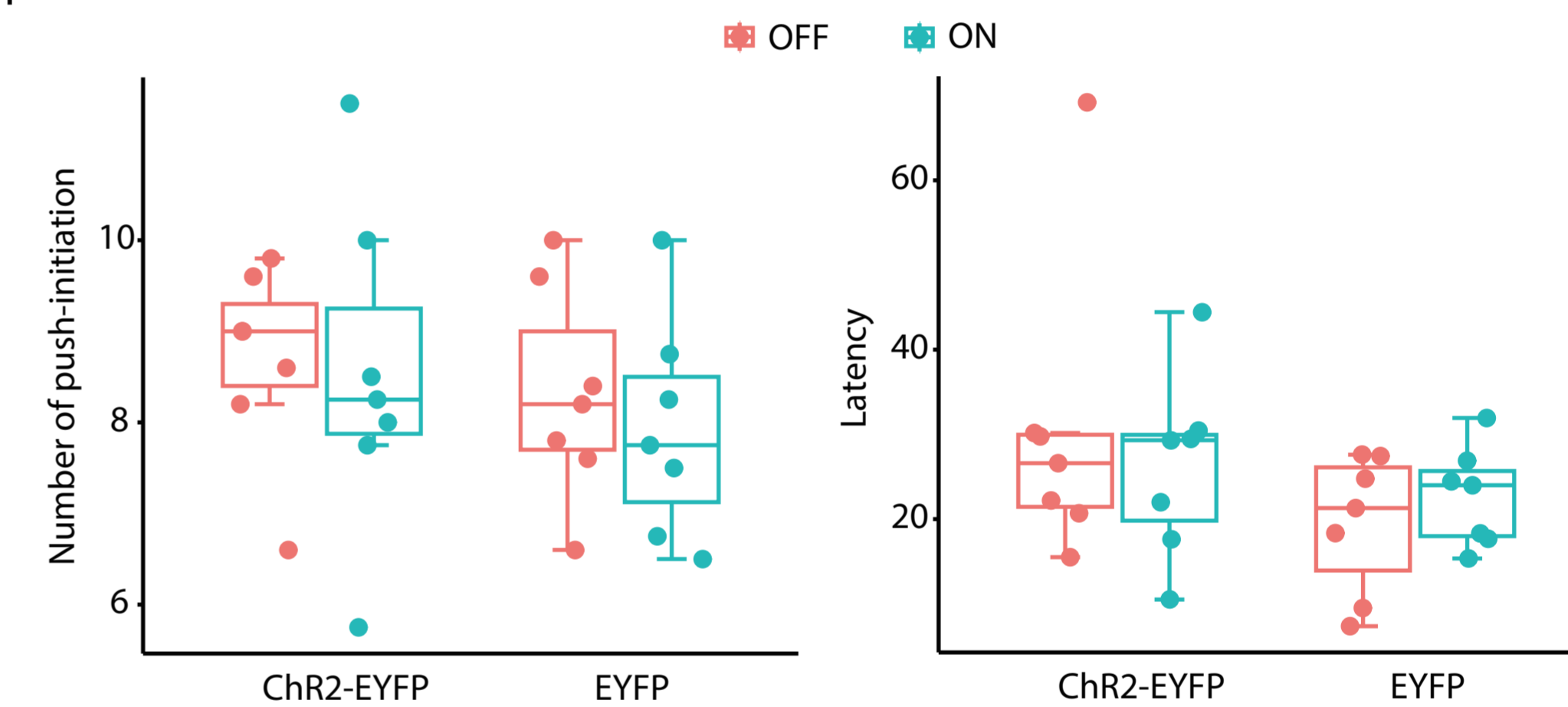

E

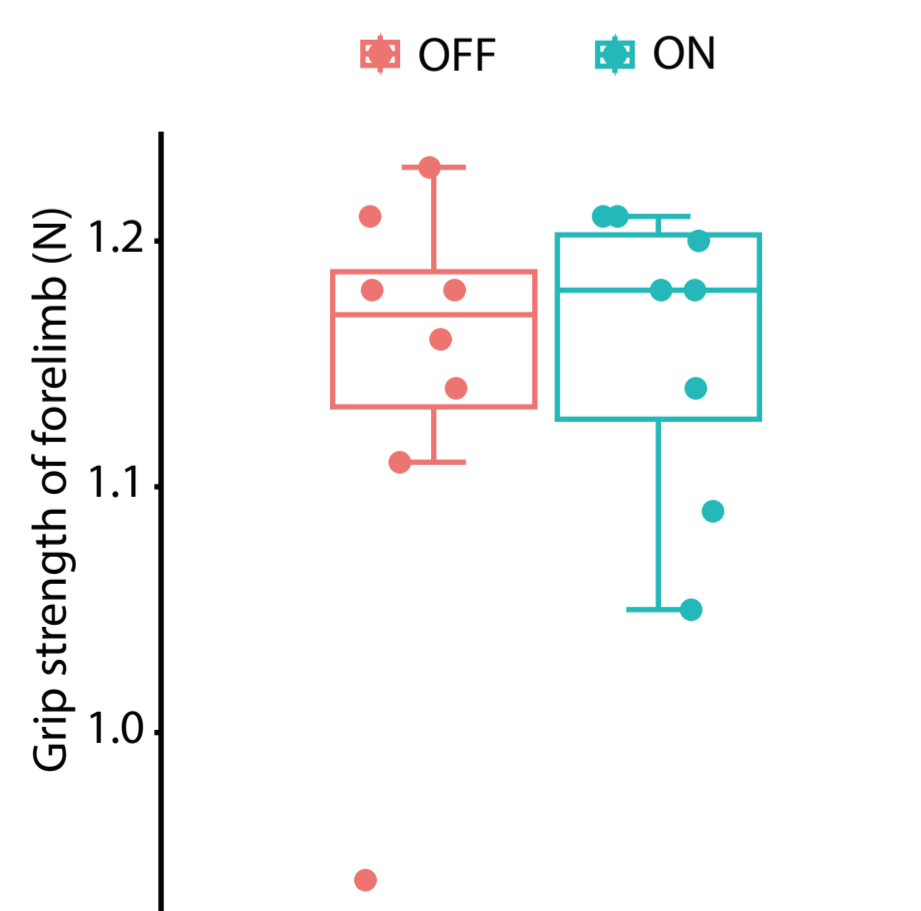

G

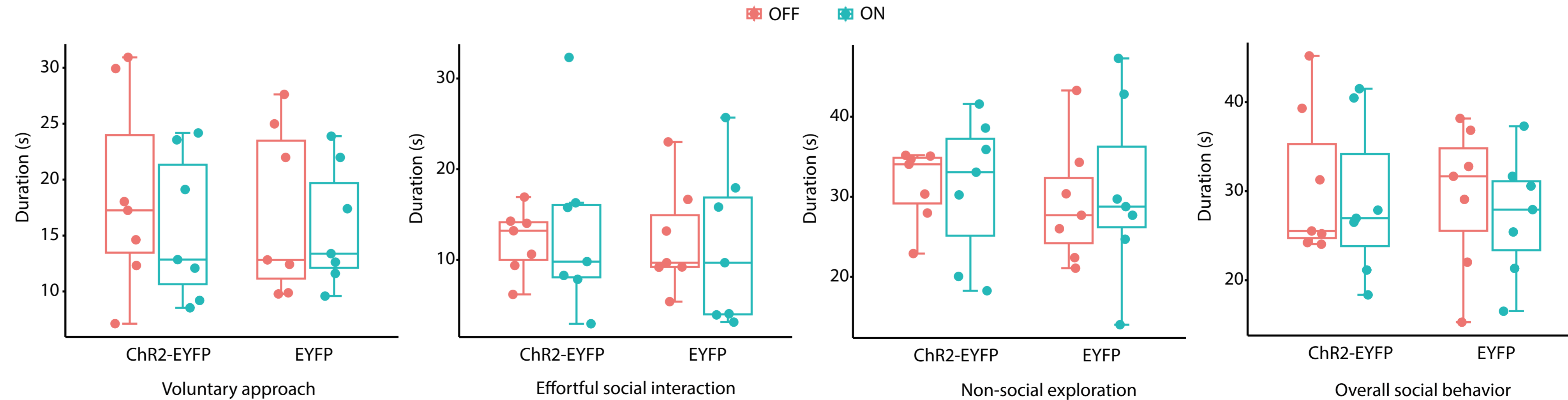

Supplement: S13 Fig — (A) Number and duration of stillness for each animal in the tube test for ChR2-EYFP mice (n = 7, Wilcoxon signed rank test; number: Z = −0.677, p = 0.498; duration: Z = −1.183, p = 0.237). (B) Percentage of time spent resisting (n = 7, Wilcoxon signed rank test; Z = −0.676, p = 0.499). (C) Both ChR2-EYFP and EYFP mice demonstrated typical levels of aggressive (n = 8 for each group, two-way repeated measures ANOVA with Bonferroni correction; F1,14 = 0.173, p = 0.684) and nonaggressive behaviors (F1,14 = 0.207, p = 0.656) in the resident-intruder test, with no significant differences observed between the groups (F1,14 = 0.782, p = 0.391 for aggressive behaviors; F1,14 = 0.06, p = 0.811 for nonaggressive behaviors). (D) Optogenetic activation of the SNrGlu-DRN pathway did not alter the number of entries into the central area (n = 7, paired two-sided t test, t6 = 1.307, p = 0.239), the time spent in the center (n = 7, paired two-sided t test, t6 = 0.941, p = 0.383), or overall locomotion (n = 7, Wilcoxon signed rank test, Z6 = −1.521, p = 0.128) during the open field test. (E) Optogenetic activation of the SNrGlu-DRN pathway did not alter forelimb muscle strength during both light-on and light-off periods (n = 8, paired two-sided t test; t7 = 0.643, p = 0.541). (F) In the push ball test, no significant main effects were observed for the number of active pushes with respect to “light condition” (n = 7 for each group; two-way repeated measures ANOVA with Bonferroni correction; F₁,₁₂ = 0.357, p = 0.561) or “group” (F₁,₁₂ = 0.755, p = 0.402). Similarly, analysis of latency revealed no significant main effects for “light condition” (F₁,₁₂ = 0.018, p = 0.895) or “group” (F₁,₁₂ = 2.703, p = 0.126). (G) In the social interaction test, duration analyses indicated no significant main effects for “light condition” (n = 7 for each group; two-way repeated measures ANOVA with Bonferroni correction): voluntary approach (F₁,₁₂ = 0.636, p = 0.441), effortful social interaction (F₁,₁₂ = [file pbio.3003687.s013.pdf]
