# Supplementary material for: Glutamatergic projections from the substantia nigra pars reticulata to the dorsal raphe nucleus regulate male social hierarchies
Source: PLoS Biol. 2026 Mar 3;24(3):e3003687. doi: 10.1371/journal.pbio.3003687 (PMC12974815; doi:10.1371/journal.pbio.3003687)
Supplement: S2 Fig — (A) Immunohistological data from two individual subjects, with each row representing a distinct animal. The left three subplots display the expression of EYFP+, CaMKIIα+, and the colocalization of EYFP+ and CaMKIIα+ within the SNr, respectively. The right pie illustrates the percentage of CaMKIIα+ neurons co-labeled with EYFP in the SNr. (B–D) Corresponding analyses for GCaMP6, ChR2-EYFP, and hM4Di-EGFP expression, respectively. Scale bar, 100 μm. (PDF) [file pbio.3003687.s002.pdf]

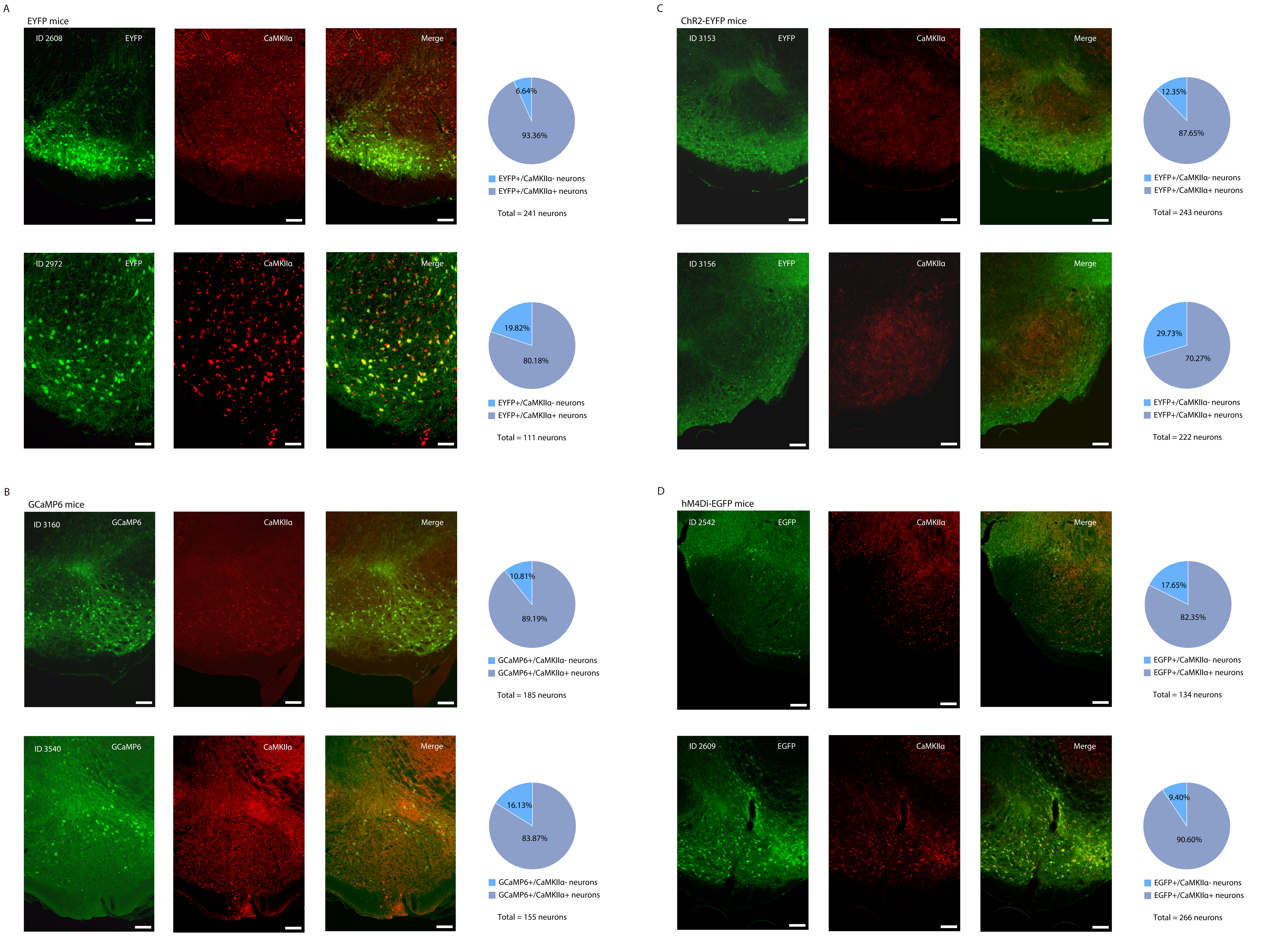
A
EYFP mice
ID 2608
EYFP
CaMKIIα
Merge
6.64%
93.36%
EYFP+/CaMKIIα- neurons
EYFP+/CaMKIIα+ neurons
Total = 241 neurons
ID 2972
EYFP
CaMKIIα
Merge
19.82%
80.18%
EYFP+/CaMKIIα- neurons
EYFP+/CaMKIIα+ neurons
Total = 111 neurons
B
GCaMP6 mice
ID 3160
GCaMP6
CaMKIIα
Merge
10.81%
89.19%
GCaMP6+/CaMKIIα- neurons
GCaMP6+/CaMKIIα+ neurons
Total = 185 neurons
ID 3540
GCaMP6
CaMKIIα
Merge
16.13%
83.87%
GCaMP6+/CaMKIIα- neurons
GCaMP6+/CaMKIIα+ neurons
Total = 155 neurons
C
ChR2-EYFP mice
ID 3153
EYFP
CaMKIIα
Merge
12.35%
87.65%
EYFP+/CaMKIIα- neurons
EYFP+/CaMKIIα+ neurons
Total = 243 neurons
ID 3156
EYFP
CaMKIIα
Merge
29.73%
70.27%
EYFP+/CaMKIIα- neurons
EYFP+/CaMKIIα+ neurons
Total = 222 neurons
D
hM4Di-EGFP mice
ID 2542
EGFP
CaMKIIα
Merge
17.65%
82.35%
EGFP+/CaMKIIα- neurons
EGFP+/CaMKIIα+ neurons
Total = 134 neurons
ID 2609
EGFP
CaMKIIα
Merge
9.40%
90.60%
EGFP+/CaMKIIα- neurons
EGFP+/CaMKIIα+ neurons
Total = 266 neurons
