# Supplementary material for: Glutamatergic projections from the substantia nigra pars reticulata to the dorsal raphe nucleus regulate male social hierarchies
Source: PLoS Biol. 2026 Mar 3;24(3):e3003687. doi: 10.1371/journal.pbio.3003687 (PMC12974815; doi:10.1371/journal.pbio.3003687)
Supplement: S3 Fig — (A) The left three subplots display the expression of EYFP+, VGLUT2+, and the colocalization of EYFP+ and VGLUT2+ within the SNr, respectively. The right pie illustrates the percentage of VGLUT2+ neurons co-labeled with EYFP in the SNr. (B–D) Similar analyses for GCaMP6, ChR2-EYFP, and hM4Di-EGFP expression, respectively. Scale bar, 60 μm. (PDF) [file pbio.3003687.s003.pdf]

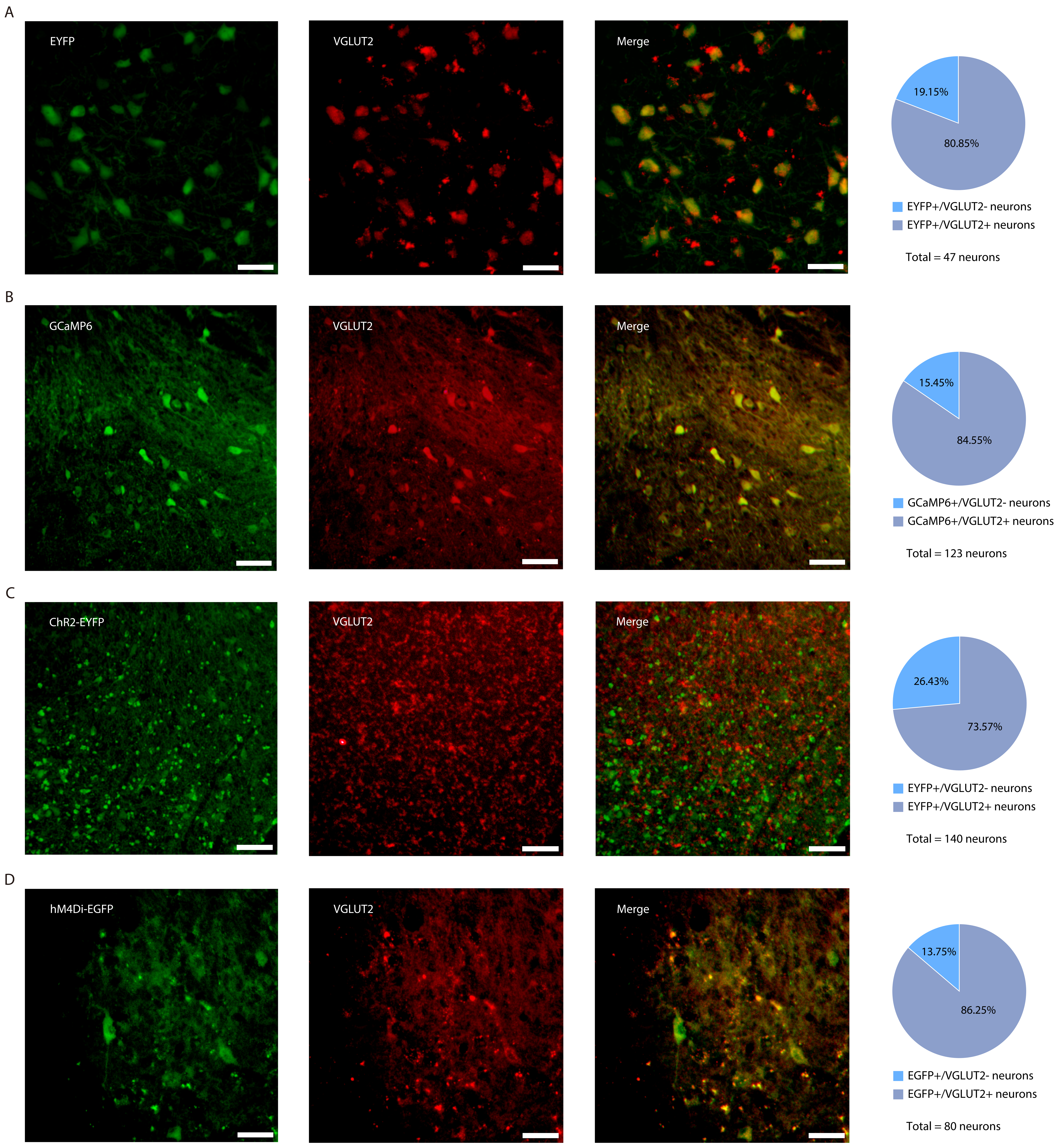
A
EYFP
VGLUT2
Merge
19.15%
80.85%
EYFP+/VGLUT2- neurons
EYFP+/VGLUT2+ neurons
Total = 47 neurons
B
GCaMP6
VGLUT2
Merge
15.45%
84.55%
GCaMP6+/VGLUT2- neurons
GCaMP6+/VGLUT2+ neurons
Total = 123 neurons
C
ChR2-EYFP
VGLUT2
Merge
26.43%
73.57%
EYFP+/VGLUT2- neurons
EYFP+/VGLUT2+ neurons
Total = 140 neurons
D
hM4Di-EGFP
VGLUT2
Merge
13.75%
86.25%
EGFP+/VGLUT2- neurons
EGFP+/VGLUT2+ neurons
Total = 80 neurons
