# Supplementary material for: Glutamatergic projections from the substantia nigra pars reticulata to the dorsal raphe nucleus regulate male social hierarchies
Source: PLoS Biol. 2026 Mar 3;24(3):e3003687. doi: 10.1371/journal.pbio.3003687 (PMC12974815; doi:10.1371/journal.pbio.3003687)
Supplement: S12 Fig — (A) Number and duration of push-initiation for each animal in the tube test (n = 7, Wilcoxon signed rank test; number: Z = −0.594, p = 0.553; duration: Z = −0.000, p = 1.000). (B) Number and duration of push-back for each animal in the tube test (n = 7, Wilcoxon signed rank test; number: Z = −0.338, p = 0.735; duration: Z = −0.676, p = 0.499). (C) Number and duration of stillness for each animal in the tube test (n = 7, Wilcoxon signed rank test; number: Z = −0.508, p = 0.611; duration: Z = −1.014, p = 0.310). (D) Percentage of time spent resisting (n = 7, Wilcoxon signed rank test; Z = −0.845, p = 0.398). (E) Percentage of time spent retreating (n = 7, Wilcoxon signed rank test; Z = −0.338, p = 0.735). (F) Daily rank positions of a sample cage of mice over 7 days, illustrating that the third-ranked mouse maintained its rank following photostimulation of SNrGlu neuron terminals in the DRN. (G) No changes in rank were observed in EYFP mice before and after the optogenetic activation of the SNrGlu-DRN pathway. Each line represents an individual animal. The data underlying this Figure can be found in file number 44 on Dryad (https://doi.org/10.5061/dryad.m0cfxppg3). (PDF) [file pbio.3003687.s012.pdf]

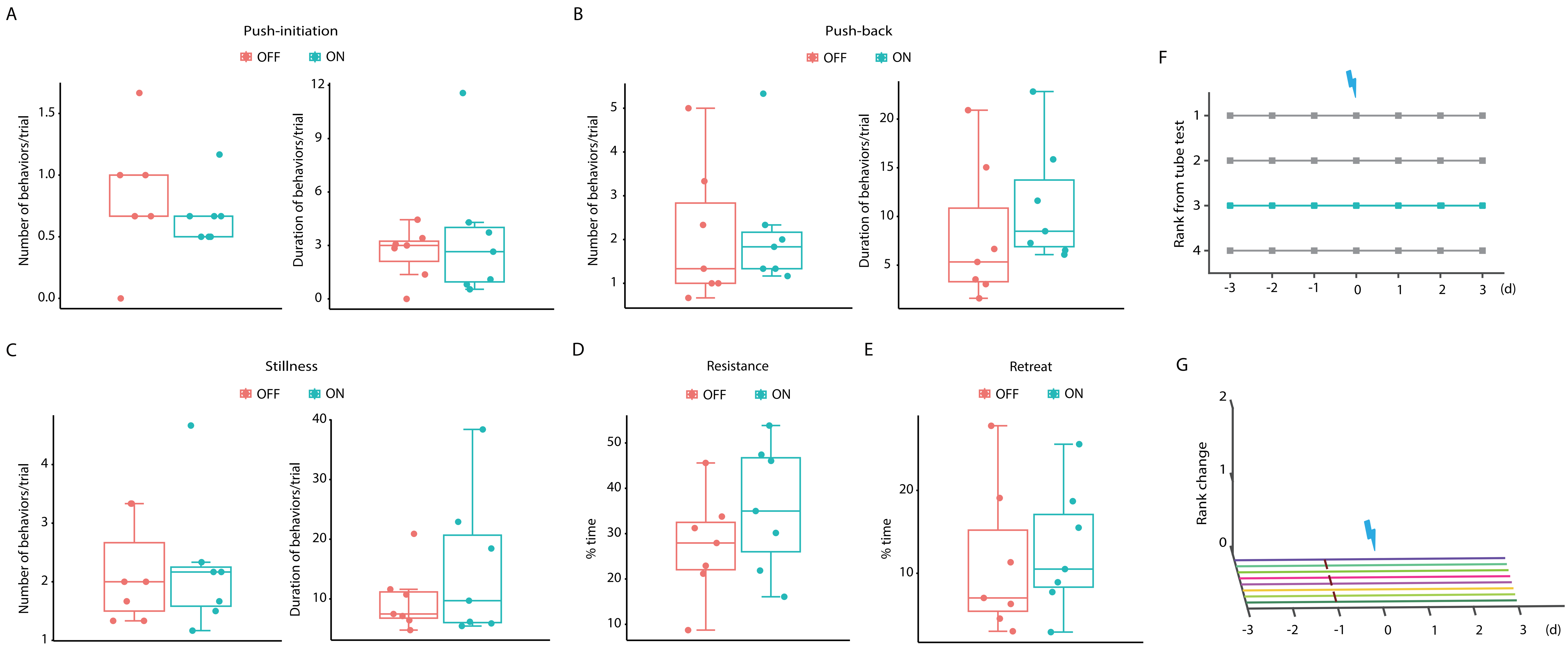

A
Push-initiation
OFF
ON
Number of behaviors/trial
Duration of behaviors/trial
B
Push-back
OFF
ON
Number of behaviors/trial
Duration of behaviors/trial
F
Rank from tube test
(d)
C
Stillness
OFF
ON
Number of behaviors/trial
Duration of behaviors/trial
D
Resistance
OFF
ON
% time
E
Retreat
OFF
ON
% time
G
Rank change
(d)
