# Supplementary material for: Glutamatergic projections from the substantia nigra pars reticulata to the dorsal raphe nucleus regulate male social hierarchies
Source: PLoS Biol. 2026 Mar 3;24(3):e3003687. doi: 10.1371/journal.pbio.3003687 (PMC12974815; doi:10.1371/journal.pbio.3003687)
Supplement: Attachment — Submitted filename: Response to Reviewers.docx [file pbio.3003687.s016.docx]

Dear Editor and Reviewers,

Thank you for providing us with the opportunity to revise and improve our manuscript. We have considered the comments seriously and have modified the manuscript accordingly as described below. Changes in the text are highlighted in blue.

Sincerely yours,

Yanzhu Fan

Guangzhan Fang

**Editor**

**Question 1.** The reviewers say that the results here are novel and relevant. However, they expressed skepticism on the level of support for the claims that the SNr-DRN pathway has a specific role in social hierarchy, and other areas requiring further controls or analyses. The reviewers have suggested many possible and potentially insightful experiments. However, considering the scope of this Short Report article, we think that it would not be appropriate to require you to perform every suggested experiment. Based on our discussion with the Academic Editor, we think that your revision should focus on providing stronger support for the main findings through new behavioral controls, stronger neuroanatomical characterizations, and addressing the statistical concerns. In particular, you should perform new experiments to address Reviewer 1's point about dissociating exertional effects from social ones, and you should respond thoroughly to all of the points raised by the reviewers.

**Answer:**

We sincerely appreciate the constructive comments and suggestions provided by you and the reviewers. In the revised manuscript, we have implemented new behavioral controls, updated the histological data, and addressed the statistical concerns raised. Specifically, our behavioral controls assess whether the behavioral parameters are affected by light stimulation during both an active physical effort task devoid of social components (pushing against a diamond-shaped rubber bouncy ball within a tube) and a social, non-competitive effort task (interacting with a novel mouse introduced into a small elevated restraint cage). Additionally, we recorded SNr^Glu^ activity during these tasks. The results indicate that neither of the two tasks significantly influenced the behavioral parameters or SNr^Glu^ activity. For further details, please refer to our response to Question 1 from Reviewer #1. Furthermore, we have updated the histological data and thoroughly addressed the statistical concerns raised by the reviewers. For detailed responses, please refer to our answers to Question 9 from Reviewer #1, Question 5 from Reviewer #2 and Question 3 from Reviewer #3.

Overall, we have strengthened the current research findings through the incorporation of new data. In the revised manuscript, we have systematically addressed all reviewers' concerns and made modifications in accordance with the technical requirements of the journal.

**Reviewer #1:**

Fan et al. employ the tube test assay, a well-established paradigm, to assess the role of glutamatergic substantia nigra pars reticulata (SNr) neurons and the SNr-to-dorsal raphe nucleus (DRN) pathway in social hierarchy in rodents. This test determines a "winner" in paired trials, where the dominant individual displaces the presumably subordinate counterpart from the confined tube space. The authors use fiber photometry to characterize the functional relevance of SNr-Glu neurons and apply optogenetic activation and chemogenetic inhibition to dissect the role of the SNr-Glu to DRN pathway. Their findings demonstrate that this pathway bidirectionally modulates social rank, with activation or inhibition respectively promoting or suppressing dominance behavior. This study is significant as social hierarchy is a fundamental organizing principle across species, yet its neural substrates remain poorly understood. And while for instance the medial prefrontal cortex (mPFC) has been implicated in encoding social rank, the contributions of the SNr and its projections to the DRN have remained largely unexplored. Thus, results are timely and of great relevance to the field. However, the manuscript requires major revisions, in particular with respect to critical control experiments which are missing and prevent an accurate interpretation of the authors' findings:

**Answer:**

We sincerely appreciate your insightful comments and constructive suggestions. In response, we have thoroughly revised the entire manuscript in accordance with your suggestions, as well as those of other reviewers and the editor. We noted that some of your comments were duplicated and contained similar content, likely due to the simultaneous submission of your comments alongside the manuscript annotated with your remarks. Consequently, we have consolidated these overlapping comments into a single entry and assigned them the same question number, commencing with 1.

Major:

**Question 1.** The authors conclude that SNr-Glu calcium activity levels are associated with social competition, specifically, rather than general physical effort. However, this claim lacks sufficient experimental support. While the authors demonstrate increased activity during tube test contests, they fail to include critical controls that would dissociate both efforts in a social context and, more specifically, social rank-related efforts from non-social physical exertion. A proper experimental design would require testing SNr-Glu activity during both an active physical effort task without social components (e.g., pushing against an inanimate barrier) and a social but non-competitive effort task. This oversight is particularly significant given the SNr's established role in integrating diverse motor and sensory signals. This fundamental limitation also applies to their optogenetic manipulation experiments of the SNr-Glu to DRN pathway.

(**Question 1.** The authors claim that the SNr-Glu "population activity is correlated with active social contests." - however, a critical control - testing if an increase in activity is solely due to the physical effort, or the increase is more specifically tied to physical efforts in social context pertaining to the expression of social rank - are missing. As a test of grip strength is rather an assessment of passive physical effort, the authors need to include a control with an active physical effort only, and, ideally an experimental control where the effort is shown in a social context but not relevant to social rank. This is especially critical, as the SNr acts as locus for the integration of a variety of sensory signals, including those relevant for the execution of motor tasks. Without these controls, the authors conclude that "These findings indicate the relationship between SNr-Glu neuronal activity and social competition and status." does not hold. This criticism also applies to optogenetic manipulations of the SNr-Glu to DRN pathway.)

**Answer:**

(1) **Supplementary Control Experiments**: In the revised version, we recorded SNr^Glu^ activity during both an active physical effort task that lacked social components (termed the push ball test, which involved pushing a diamond-shaped rubber bouncy ball within a tube) and a social, non-competitive effort task (designated as the social interaction test, involving interaction with a novel mouse introduced into a small elevated restraint cage).

The experimental procedure for the push ball test closely resembled that of the tube test, with the following modifications: a 15 cm-long acrylic tube with an inner diameter of 3 cm was provided, along with a 2.5 cm-diameter diamond-shaped rubber bouncy ball, both placed in the cage one week in advance to allow for animal acclimatization. Furthermore, the push ball test utilized a 45 cm-long tube, with the ball positioned 15 cm from one end. The mouse entered from this end and pushed the ball forward through the tube (a distance of 30 cm) to complete one trial. A total of four trials were conducted (two entries from each end). Ca²⁺ signals were recorded throughout the process, with analyses centered on the moment that the mouse initiated ball pushing, designated as event time 0. For optogenetic manipulation, light stimulation was applied as the mouse prepared to enter the tube, following a similar protocol. The number of active pushes and latency required to move the ball out of the tube were analyzed under both light-stimulated and non-stimulated conditions, with each subject undergoing four trials under each light condition. Statistical analyses were based on the averages of behavioral parameters.

The social interaction test was conducted using a procedure analogous to that of the social memory test. An 18 cm-high and 10 cm-diameter restraint cage was positioned on a platform (4 cm-high and 10 cm in diameter) at the midpoint of the long side of the test chamber (40 cm × 20 cm, devoid of partitions). Each test mouse was allowed to acclimate in the chamber for 10 minutes prior to the introduction of a novel mouse into the restraint cage. During the subsequent 5-minute session, the test mouse was free to approach and explore the novel mouse within the cage. Ca²⁺ signals were recorded throughout this interaction. The analysis of calcium signals was based on two distinct event-0 time points: the initiation of the test mouse's movement toward the novel mouse and the onset of lifting the head to sniff or forelimb elevation in preparation to contact the lower edge of the restraint cage. For optogenetic manipulation, light stimulation and no stimulation were alternated in 1-minute cycles throughout the 5-minute interaction period. Statistical analyses focused on the duration of specific behaviors observed during exploration, including voluntary approach (from the moment the test mouse initiated movement toward the novel mouse until it approached the restraint cage), effortful social interaction (lifting the head to sniff, or raising the forelimb and touching the lower edge of the restraint cage to sniff), non-social exploration (exploring areas beyond the restraint cage), and overall social behavior (comprising both voluntary approach and effortful social interaction).

(2) **The results for SNr neurons**

No significant changes in calcium signals associated with active pushes were observed in GCaMP6f-expressing or EYFP-expressing mice subjected to the push ball test (*p* > 0.05; permutation test; see the S4 Fig please). Similarly, no significant alterations in calcium signals related to social interactions were detected in either group of mice during the social interaction test (*p* > 0.05; permutation test; see the S5 Fig please). These findings suggest that calcium activity levels in SNr^Glu^ neurons are not associated with physical effort in an active task devoid of social components, nor with physical effort in a social, non-competitive context.

For supplementary optogenetic experiments, the analysis of latency in the push ball test revealed no significant main effects for "light condition" (n=7 for both the ChR2-EYFP and EYFP groups; two-way repeated measures ANOVA with Bonferroni correction; F₁,₁₂=0.131, *p*=0.723) or "group" (F₁,₁₂=0.336, *p*=0.573). Similarly, the assessment of the number of active pushes showed no significant main effects for "light condition" (F₁,₁₂=0.220, *p*=0.647) or "group" (F₁,₁₂=0.079, *p*=0.783). In the social interaction test, the duration analysis also indicated no significant main effects for "light condition" (n=7 for both the ChR2-EYFP and EYFP groups; two-way repeated measures ANOVA with Bonferroni correction): voluntary approach (F₁,₁₂=1.700, *p*=0.217), effortful social interaction (F₁,₁₂=0.017, *p*=0.897), overall social behavior (F₁,₁₂=0.696, *p*=0.421), and non-social exploration (F₁,₁₂=0.314, *p*=0.586). Furthermore, no significant main effects were observed for "group" in the same parameters: voluntary approach (F₁,₁₂=0.064, *p*=0.804), effortful social interaction (F₁,₁₂=0.549, *p*=0.473), overall social behavior (F₁,₁₂=0.551, *p*=0.472), and non-social exploration (F₁,₁₂=0.128, *p*=0.727).

Collectively, the results indicate that calcium activity levels in SNr^Glu^ neurons are not correlated with physical effort in the active task devoid of social components, nor with physical effort in a social but non-competitive task. Furthermore, optogenetic activation of SNr^Glu^ neurons does not exert any influence on behavioral parameters in these two tasks lacking social competition.

(3) **The results for SNr^Glu^-DRN pathway**

Analysis of latency in the push ball test revealed no significant main effects for "light condition" (n=7 for both the ChR2-EYFP and EYFP groups; two-way repeated measures ANOVA with Bonferroni correction; F₁,₁₂=0.018, *p*=0.895) or "group" (F₁,₁₂=2.703, *p*=0.126). Similarly, no significant main effects were observed for the number of active pushes with respect to "light condition" (F₁,₁₂=0.357, *p*=0.561) or "group" (F₁,₁₂=0.755, *p*=0.402). In the social interaction test, duration analyses also indicated no significant main effects for "light condition" (n=7 for both the ChR2-EYFP and EYFP groups; two-way repeated measures ANOVA with Bonferroni correction): voluntary approach (F₁,₁₂=0.636, *p*=0.441), effortful social interaction (F₁,₁₂=0.009, *p*=0.927), overall social behavior (F₁,₁₂=0.348, *p*=0.566), and non-social exploration (F₁,₁₂=0.023, *p*=0.882). Additionally, no significant main effects were found for "group" across the same parameters: voluntary approach (F₁,₁₂=0.063, *p*=0.807), effortful social interaction (F₁,₁₂=0.057, *p*=0.815), overall social behavior (F₁,₁₂=0.279, *p*=0.607), and non-social exploration (F₁,₁₂=0.188, *p*=0.672).

Collectively, optogenetic activation of the SNr^Glu^-DRN pathway does not influence behavioral parameters in either of these tasks that are devoid of social competition.

The original sentence in Line 129,

“These findings indicate the relationship between SNr^Glu^ neuronal activity and social competition and status.”

has been changed to

“To investigate whether the population activity of SNr^Glu^ neurons is specifically linked to physical efforts in a social context related to the representation of social rank, we conducted both an active physical effort task devoid of social components (the push ball test) and a social, non-competitive effort task (the social interaction test). No significant changes in calcium signals associated with pushes were observed in GCaMP6f-expressing or EYFP-expressing mice during the push ball test (*p* > 0.05; permutation test; S4 Fig). Likewise, no significant alterations in calcium signals related to effortful social interactions were detected in either group of mice throughout the social interaction test (*p* > 0.05; permutation test; S5 Fig). These results suggest that calcium activity levels in SNr^Glu^ neurons are not correlated with physical effort in the absence of social competition. Collectively, these findings underscore the relationship between SNr^Glu^ neuronal activity and social competition and status.”

The following sentences have been added to Line 190,

“Importantly, the push ball test demonstrated that optogenetic activation of SNr^Glu^ neurons did not significantly affect either the latency required to move the ball out of the tube or the number of active pushes exhibited by the mice (S7E Fig). Similarly, the social interaction test indicated that the same activation did not influence the mice's effort-based social behavior or their non-social exploratory activities (S7F Fig). Collectively, these findings suggest that optogenetic activation of SNr^Glu^ neurons does not influence physical effort in the active task devoid of social components, nor does it affect physical effort in the social yet non-competitive context.”

The following sentences have been added to Line 325,

“Importantly, the push ball test demonstrated that optogenetic activation of SNr^Glu^-DRN pathway did not significantly alter either the latency required to move the ball out of the tube or the number of active pushes exhibited by the mice (S13F Fig). Similarly, the social interaction test revealed that the same activation did not affect the mice's effort-based social behaviors or their non-social exploratory activities (S13G Fig). Consequently, these findings suggest that optogenetic activation of SNr^Glu^-DRN pathway does not impact physical effort in the task that lack social components, nor does it influence physical effort in social contexts that are non-competitive.”

The following sentences have been added to Line 372,

“It is noteworthy that calcium activity levels in SNr^Glu^ neurons are not correlated with physical effort in the active task devoid of social components, nor with physical effort in the social yet non-competitive context. Furthermore, optogenetic activation of SNr^Glu^ neurons did not influence effort-related behaviors that are independent of social competition. These findings further corroborate the role of SNr^Glu^ neurons in regulating social hierarchy among mice.”

The following paragraph has been added to Line 501,

“**Push ball test.** To differentiate social rank-related efforts from non-social physical exertion, we conducted the push ball test. The experimental procedure for this test closely resembled that of the tube test, with the following modifications: a 15 cm-long acrylic tube with an inner diameter of 3 cm was provided, along with a 2.5 cm-diameter diamond-shaped rubber bouncy ball, both placed in the cage one week in advance to allow for animal acclimatization. Furthermore, the push ball test utilized a 45 cm-long tube, with the ball positioned 15 cm from one end. The mouse entered from this end and pushed the ball forward through the tube (the ball travels a distance of 30 cm) to complete one trial. A total of four trials were conducted (two entries from each end). The moment the mouse initiated ball pushing was designated as event time 0 for Ca²⁺ signal analysis. Additionally, we analyzed the number of active pushes and the latency required to displace the ball from the tube under both light-stimulated and non-stimulated conditions for optogenetic manipulation.”

The following paragraph has been added to Line 554,

“**Social interaction test**. To distinguish social rank-related efforts from social, non-competitive physical exertion, we performed the social interaction test. The test was conducted using a procedure analogous to that of the social memory test. An 18 cm-high and 10 cm-diameter restraint cage was positioned on a platform (4 cm-high and 10 cm in diameter) at the midpoint of the long side of the test chamber (40 cm × 20 cm, devoid of partitions). Each test mouse was allowed to acclimate in the chamber for 10 minutes prior to the introduction of a novel mouse into the restraint cage. During the subsequent 5-minute session, the test mouse was free to approach and explore the novel mouse within the cage. For fiber photometry recording, the analysis of calcium signals was anchored to two distinct event time points: the initiation of the test mouse's movement toward the novel mouse and the onset of lifting the head to sniff or forelimb elevation in preparation for contacting the lower edge of the restraint cage. In the context of optogenetic manipulation, analyses concentrated on the duration of specific behaviors, which included voluntary approach (defined as the period from the moment the test mouse began to move toward the novel mouse until it reached the restraint cage), effortful social interaction (encompassing head lifting to sniff or raising the forelimb to touch the lower edge of the restraint cage for sniffing), non-social exploration (involving exploration of areas beyond the restraint cage), and overall social behavior (comprising both voluntary approach and effortful social interaction).”

The following paragraph has been added to Line 649,

“For the push ball and social interaction tests, two-way repeated measures ANOVA with Bonferroni correction was employed, considering the variables of “light condition” and “group”. Similarly, for the pole and rotarod tests, two-way repeated measures ANOVA with Bonferroni correction was utilized, focusing on the variables of “injection (CNO vs. saline)” and “group”.”

Additionally, we have revised and included the pertinent figures along with their accompanying legends. For further details, please refer to the main text and Supporting information.

**Question 2.** The study fails to establish how SNr-Glu calcium activity relates to social rank. The fiber photometry data in Fig. 1 only shows event-locked activity during pushing behaviors but provides no information about calcium activity differences between mice of different ranks. This could be furthermore supported by the quantification of the actual physical effort exerted during tube test contests based on more in-depth behavioral analysis, making it possible to determine how fiber photometry calcium activity patterns scale with effort intensity vs rank.

(**Question 2.** Related to point 1, it is not clear how the fiber photometry signal relates to the ranks of the tested individuals. This information is also missing from Figure 1, although it would be important to understand how the mean activity of SNr-Glu neurons differs between mice of different ranks. This analysis might give better insights into concerns raised in point 1, by contrasting the signal evoked related to rank and those evoked by behavioral read-outs of physical efforts, the latter at the time missing from Fig. 1 and from any other analysis in the manuscript. Thus, related to that the authors should quantify measures of "effortful behaviors".)

**Answer:**

We sincerely appreciate your insightful suggestion. Analyzing the interplay among the activity of SNr^Glu^ neurons, the social hierarchies of mice, and their physical efforts may provide valuable insights into the mechanisms underlying the formation and maintenance of social hierarchies, as well as their dynamic characteristics. Intuitively, one could infer that individuals exhibiting higher levels of physical effort demonstrate increased SNr^Glu^ neuronal activity and achieve elevated social ranks. Concurrently, those occupying higher social ranks may engage in more effortful behaviors to sustain their dominant status.

Currently, our fiber optic recordings encompass data from only six mice (three in Rank 1, and one each in Ranks 2, 3, and 4), which is insufficient for a comprehensive analysis. Utilizing the available data, we calculated the area under the curve (AUC) for the normalized ΔF/F values for each subject across 1-second time windows (from 0 to 1 second), with the onset of pushes designated as the zero point. The subsequent figure illustrates the relationship between SNr^Glu^ neuronal activity and the social hierarchies of the mice.

To investigate the relationship among the three aforementioned factors, a minimum of 32 mice is required, with four mice per cage distributed across eight cages. We did not conduct supplementary experiments to examine this relationship for several reasons. First, the primary objective of this study was to establish whether the SNr^Glu^ neurons is involved in regulating social hierarchies in mice. Following the incorporation of control experiments and additional data suggested by you and other reviewers, our current results already confirm the involvement of the SNr^Glu^ neurons in modulating mouse social hierarchies. Second, elucidating the interplay among SNr^Glu^ neuronal activity, mouse social hierarchies, and physical exertion represents a critically important question. This investigation would further elucidate the mechanisms by which SNr^Glu^ neurons regulate the formation and maintenance of social hierarchies, establishing a foundational focus for future research that builds upon the findings presented in this manuscript.

The following sentences have been added to Line 374,

“Nevertheless, the quantitative relationship among the activity of SNr^Glu^ neurons, the social hierarchies of mice, and their physical exertion requires further exploration. Investigating this interplay may provide deeper insights into the mechanisms by which SNr^Glu^ neurons regulate the formation and maintenance of social hierarchies in mice.”

**Question 3.** The potential lateralization of SNr-Glu signals during social competition remains unexplored, given that the calcium activity signal is related to physical effort, this would be a further important measure to dissociate physical effort from the display of social rank.

(**Question 3.** Given that the authors link the onset of the physical effort to SNr fiber photometry signal, do they observe any lateralization of the signal?)

**Answer:**

Indeed, investigating the potential lateralization of SNr^Glu^ signals during social competition is a compelling avenue of research. To ensure effective signal acquisition in each mouse and mitigate potential confounding influences related to lateralization, we implanted fiber optic cannulas bilaterally in the SNr. During calcium signal acquisition, half of the subjects selected randomly had signals recorded from the left SNr, while the other half had signals recorded from the right SNr. Pooling and analyzing these data produced a signal that approximates a bilateral average, thus avoiding unilateral biases. However, since we did not collect calcium signals simultaneously from both sides of the same mouse, we are unable to ascertain whether lateralization occurs in the SNr during social competition. This question, however, warrants further investigation in future studies.

To dissociate physical effort from the expression of social rank, we conducted two additional control experiments, specifically the push ball test and the social interaction test, as suggested by you, other reviewers, and the editor. The results indicate that calcium activity signals in SNr^Glu^ neurons are not associated with physical effort in the absence of social competition. For further details, please refer to our response to Question 1.

The following sentence has been added to Line 610,

“To ensure effective signal acquisition in each mouse and to mitigate possible confounding effects associated with potential lateralization of the SNr, calcium signals were recorded from the left SNr in half of the randomly selected subjects, while the remaining subjects had signals recorded from the right counterpart.”

**Question 4.** (1) The interpretation of optogenetic SNr-Glu activation results is equally affected by these limitations regarding effort specificity. While the authors show that stimulation increases tube test success, they do not adequately address whether this reflects a genuine modulation of social hierarchy versus a non-specific increase in physical effort capacity. (2) Moreover, their observation that elevated ranks persist for four days could alternatively be explained by the well-documented "winner effect" in mice, where initial competitive success increases probability of future wins through behavioral reinforcement rather than direct neural encoding of social status, which here could be given rise to by a modulation of physical effectiveness.

(**Question 4.** (1) Optogenetic activation of SNR-Glu neurons: This experimental manipulation is subject to the same criticism as articulated under aim 1. Namely, without an appropriate control, it is difficult to interpret results with respect to social rank as they might largely reflect changes in active motor effort. (2) Moreover, while the authors write that "Compared to controls, the significantly elevated social ranks were maintained for at least four days (Fig 2I-2K)". However, it is known that in mice, winning in the tube test increases the propensity of future competitive success ("winner effect"). Thus, in principle, experimental manipulations as conducted here could increase physical effort which induces the winner effect based on the initial success.)

**Answer:**

(1) In the revised version, we performed two supplementary control experiments, including the push ball test and the social interaction test, during which we recorded SNr^Glu^ activity and executed optogenetic activation of SNr^Glu^ neurons. The results demonstrate that calcium activity levels in SNr^Glu^ neurons are not correlated with physical effort in the active task devoid of social components (the push ball test) or with physical effort in a social but non-competitive context (the social interaction test). Furthermore, optogenetic activation of SNr^Glu^ neurons and the SNr^Glu^-DRN pathway did not influence behavioral parameters in either of these tasks lacking social competition. Collectively, these results, including those from the original version and the supplementary control experiments, indicate that SNr^Glu^ reflects modulation of social hierarchy rather than a non-specific enhancement of physical effort capacity. Please refer to the response to Question 1.

(2) In the present study, we observed that optogenetic activation exerts a prolonged influence on social rank. This finding is consistent with previous researches demonstrating the enduring impact of optogenetic manipulation on alterations in social hierarchy (Fan et al., 2025; Zhou et al., 2017; Zheng et al., 2025). The establishment of a dominance hierarchy can be attributed to a reinforcing mechanism known as the “winner effect”, wherein prior victories enhance the likelihood of future successes, thereby significantly shaping the dynamics of social hierarchy and competitive motivation. Specifically, repeated competitive successes reinforce dominance behaviors, creating positive feedback loops that stabilize hierarchical structures. In mice, this winner effect is supported by synaptic strengthening within the circuit connecting the mediodorsal thalamus (MDT) to the dorsomedial prefrontal cortex (dmPFC), as evidenced by experiments using the tube test (Zhou et al., 2017). Thus, synaptic plasticity within the MDT-dmPFC circuit is essential for the winner effect in mammals. Notably, female mice exhibit reduced long-term potentiation (LTP) at MDT-to-dmPFC synapses compared to their male counterparts, alongside heightened excitability of dmPFC parvalbumin interneurons (PV-INs) (Zheng et al., 2025). Modulation of dmPFC PV-IN activity regulates LTP and the winner effect in a sexually dimorphic manner, suggesting that dmPFC PV-INs may serve as a target for enhancing the winner effect. Please also refer to the answer to Question 8.

The following sentences have been added to Line 397,

“Consistent with previous findings[25, 67, 68], the present results demonstrate a lasting impact of optogenetic manipulation on alterations in social hierarchy, particularly when compared to chemogenetic activation. The establishment of a dominance hierarchy can be attributed to a reinforcing mechanism known as the “winner effect”, in which prior victories increase the likelihood of future successes, thereby significantly influencing the dynamics of social hierarchy and competitive motivation. In mice, this winner effect can be mediated by synaptic strengthening within the circuit connecting the mediodorsal thalamus (MDT) to dmPFC[25]. Due to its millisecond-scale precision in neuronal activation, optogenetics likely facilitates the winner effect, which is supported by synaptic strengthening within the relevant neural circuits[25, 68]. Consequently, the enduring impact of optogenetics on social rank arises not from a prolonged duration of neuronal activity, but rather from its precise timing during critical behavioral events, enhancing synaptic plasticity within the associated neural circuitry. In contrast, although chemogenetic activation may have a longer-lasting effect, it lacks the temporal specificity necessary for inducing synaptic plasticity. Further research is warranted to investigate whether the mechanisms of synaptic plasticity within the circuit associated with the SNr also contribute to the winner effect.”

***References for this answer (these reference are included in the rfevised version)***

Fan Y, Lu L, Ge S, Wu Y, Wang Z, Qing T, Shi S, Fang G. GABAergic neurons in the dorsal raphe nucleus regulate social hierarchy in mice. Communications Biology, 2025, 8(1): 1158

Zhou, T.T., Zhu, H., Fan, Z.X., Wang, F., Chen, Y., Liang, H.X., Yang, Z.F., Zhang, L., Lin, L.N., Zhan, Y., et al. (2017). History of winning remodels thalamo-PFC circuit to reinforce social dominance. Science 357, 162-168.

Zheng D, Xin Q, Jin S, Zhou A, Jia X, Tan Y, Hu H. Neural mechanism of the sexually dimorphic winner effect in mice. Neuron, 2025: doi: 10.1016/j.neuron.2025.1009.1029

**Question 5.** The authors' conclusion that SNr-Glu activation does not affect anxiety-like behavior, based on elevated plus maze (EPM) results, is potentially compromised by methodological limitations. Their experimental design lacks critical no-light control groups that would account for non-specific effects of light delivery itself, which is known to potentially influence rodent behavior in anxiety assays.

(**Question 5.** The authors write "Notably, the duration of time spent in the open arms prior to light activation in the elevated plus-maze (EPM) test was significantly greater than that observed during and after light-on periods; however, no differences in open arm time were found between ChR2-EYFP and EYFP mice (S2A Fig), indicating that optogenetic activation of SNrGlu neurons does not influence anxiety-like behaviors." Given the non-specific effect of light administration, it is not clear how this is to be interpreted. The authors should include no-light control groups into their experimental design.)

**Answer:**

We sincerely appreciate and understand your suggestion. It is standard practice not to subject the same group of mice to repeated EPM testing, as adaptation may lead to confounding results in subsequent trials (Tucker and McCabe, 2017). To ascertain whether light administration itself affects EPM outcomes, the inclusion of an additional no-light control group would indeed be necessary. However, one of our research objectives is to investigate the effects of SNr^Glu^ activation but not light administration itself on anxiety. Additionally, we have incorporated an EYFP control group to account for the potential influence of light exposure. Therefore, we contend that the inclusion of no-light control groups (for both ChR2-EYFP and EYFP mice) is not required in the context of the current study.

Our current experimental design comprises two groups: ChR2-EYFP and EYFP mice. Both groups undergo an identical photostimulation protocol, with light stimulation applied exclusively during the middle 3 minutes of the 9-minute testing period. First, we suppose that a significant difference will emerge between the two groups during light exposure. The absence of significant differences in the first 3 minutes of the EPM test (without light exposure, formerly designated as S2A Fig and now labeled as S7A Fig) suggests similar baseline anxiety levels. Even if light stimulation itself induces alterations in anxiety, we can infer that these changes would be comparable in both groups. Therefore, any significant difference observed during light exposure should be attributed to SNr^Glu^ activation. Second, should we find no significant differences between the groups during light exposure, it would indicate that SNr^Glu^ activation does not influence anxiety levels. However, the potential impact of light exposure on anxiety remains uncertain, as it may induce similar baseline anxiety changes in both groups. To isolate the effect of light exposure alone, the inclusion of no-light control groups would be necessary.

In summary, while our current experimental design effectively assesses whether SNr^Glu^ activation influences anxiety levels, it does not determine the effects of light exposure itself on anxiety. One of our objectives is to investigate the former.

The original sentence in Line 183,

“…indicating that optogenetic activation of SNr^Glu^ neurons does not influence anxiety-like behaviors.”

has been change to

“…indicating that optogenetic activation of SNr^Glu^ neurons, rather than light exposure alone, does not influence anxiety-like behaviors.”

***References for this answer***

Tucker LB, McCabe JT. Behavior of male and female C57BL/6J mice is more consistent with repeated trials in the elevated zero maze than in the elevated plus maze. Frontiers in Behavioral Neuroscience, 2017, 11: 13

**Question 6.** The choice to use chemogenetic rather than optogenetic inhibition represents a curious methodological decision that is not adequately justified. Given the distinct temporal dynamics of these techniques (with chemogenetic effects lasting hours compared to milliseconds-scale optogenetic control), the authors' findings of transient rank modulation would benefit from complementary optogenetic inhibition experiments.

(**Question 6.** Given the distinct that opto- and chemogenetic manipulations are associated with different timescales where the actuators are active (chemogenetic silencing lasting several hours), it is not clear why the authors have not applied optogenetic inhibition for silencing experiments.)

**Answer:**

It is well established that social recognition via olfactory cues is essential for mice to identify their own social status (Borak et al., 2016). Pheromone signals, such as darcin, are abundant in the urine of dominant individuals, indicating that hierarchical behavior may integrate information regarding both self and opponent rank (Borak et al., 2025). Notably, male mice use chemosensory signals to infer dominance status of the opponents during social interactions (Borak et al., 2025). In the present study, inhibition experiments targeted individuals with relatively high social rank within the same cage, while their opponents maintained relatively low social rank. Furthermore, the duration of optogenetic manipulation for each mouse typically spanned only a few minutes (1-2 minutes per trial). Given such a brief timeframe, it poses a challenge for individuals who experience changes in social rank to effectively convey their new social status through scent cues.

In a preliminary experiment employing optogenetic inhibition of SNr^Glu^-DRN pathway with four mice, we observed that exposure to yellow light stimulation resulted in significantly increased periods of immobility and reduced active competitive behavior in the test subject, despite the opponent ultimately retreating. We hypothesize that two primary factors contribute to this outcome: First, due to the winner effect, lower-ranking opponents are more likely to concede defeat. Second, the internal state changes (including competitive motivation) in the subject following optogenetic inhibition are not immediately reflected via corresponding external behaviors, such as active retreat, nor are they accompanied by scent cues. Consequently, opponents are unable to detect the internal state changes in the subjects and may perceive the subjects' social rank as still superior, thus lacking a stronger competitive motivation. In contrast, when optogenetic activation targeted a lower-ranking individual within the same cage, while the opponents held relatively higher ranks, the subjects exhibited an immediate increase in effortful competitive behaviors that were readily perceived by the opponents. This dynamic facilitated rank changes, ultimately leading to the emergence of a distinct winner effect after several rounds of testing.

The circumstances surrounding chemogenetic inhibition experiments differ significantly. We conducted the tube test following the administration of CNO at intervals of 1–1.5, 3–5, 6–8, 24, 48, and 72 hours. Throughout this extended timeframe, the test subject's scent cues (e.g., through urine) may undergo changes, which can be detected by the opponents in the housing cage. This facilitates a greater likelihood of rank reversal during the tube test. Consequently, many studies investigating the regulation of social hierarchy in mice adopt a combined approach utilizing both optogenetics and chemogenetics to bidirectionally modulate relevant brain regions and their associated circuits (Zhou et al., 2017; Xin et al., 2025; Zheng et al., 2025). In this study, we employed optogenetic modulation for activation and chemogenetic methods for inhibition.

The following sentences has been added to Line 589,

“Male mice utilize chemosensory signals to assess the dominance status of their opponents during social interactions[92]. A preliminary experiment revealed that optogenetic inhibition of SNr^Glu^ neurons led to increased periods of immobility and reduced active competitive behavior in the test subject; however, the opponent ultimately retreated, potentially due to the winner effect or a lack of chemosensory signals reflecting the internal state changes in the test subject. Consistent with numerous studies employing both optogenetics and chemogenetics to bidirectionally modulate relevant brain regions and their associated circuits[25, 27, 68], we also utilized chemogenetic methods for inhibition. This choice was informed by the possibility that, during the intervals between tests, the subject's chemosensory cues may change and disperse (e.g., via urine) within the housing cage.”

***References for this answer (these references are included in the revised version)***

Borak N, Wai P, Villamayor PR, Claxton PM, Liang S, Kohl J. Dominance rank inference in mice via chemosensation. Current Biology, 2025, 35(11): 2746–2751

Williamson CM, Lee W, Curley JP. Temporal dynamics of social hierarchy formation and maintenance in male mice. Animal Behaviour, 2016, 115: 259-272

Xin Q, Zheng D, Zhou T, Xu J, Ni Z, Hu H. Deconstructing the neural circuit underlying social hierarchy in mice. *Neuron*, 2025, 113: 1-16

Zhou, T.T., Zhu, H., Fan, Z.X., Wang, F., Chen, Y., Liang, H.X., Yang, Z.F., Zhang, L., Lin, L.N., Zhan, Y., et al. (2017). History of winning remodels thalamo-PFC circuit to reinforce social dominance. Science 357, 162-168.

Zheng D, Xin Q, Jin S, Zhou A, Jia X, Tan Y, Hu H. Neural mechanism of the sexually dimorphic winner effect in mice. Neuron, 2025: doi: 10.1016/j.neuron.2025.1009.1029

**Question 7.** The apparent discrepancy between cell body and terminal manipulation effects on anxiety-related behavior requires further explanation. While SNr-Glu neuron activation shows no effect on EPM performance, stimulation of their DRN terminals does reduce anxiety-like behavior.

(**Question 7.** The EPM shows no anxiety difference (Supplementary Fig. 2a), but the DRN terminal activation does reduce anxiety (Fig. 4i). This inconsistency suggests SNrGlu cell-body vs. terminal effects may differ.)

**Answer:**

The present findings suggest that optogenetic activation of the soma of SNr^Glu^ neurons and their terminals in the DRN elicits distinct effects on anxiety. This discrepancy may arise from several potential factors.

First, numerous studies have established the role of the DRN in modulating anxiety levels in mice. Serotonin (5-HT) is a critical modulator of anxiety circuits (Lowry et al., 2005), with the DRN serving as the primary source of 5-HT in the brain. Dysfunction within the central DRN^5-HT^-bed nucleus of the stria terminalis (BNST) neural circuit represents a significant neurobiological basis for anxiety and fear disorders (Marcinkiewcz et al., 2016; Linley et al., 2017; Zheng et al., 2024). Under conditions of stress, 5-HT neurons interact with BNST receptors to either mitigate or exacerbate anxiety and fear responses. In the BNST, corticotropin-releasing factor (CRF) neurons modulate emotional responses by inversely regulating excitatory and inhibitory 5-HT neurons. Furthermore, 5-HT∩vGluT3 neurons projecting to basal amygdala (BA) parvalbumin (PV) (DRN^5-HT∩vGluT3^-BA^PV^) and pyramidal (Pyr) (DRN^5-HT∩vGluT3^-BA^Pyr^) neurons exhibit distinct intrinsic properties and gene expression profiles, responding differentially to anxiogenic and social stimuli (Yu et al., 2022). Activation of DRN^5-HT∩vGluT3^→BA^PV^ inhibits 5-HT release via GABAB receptors on serotonergic terminals in the BA, leading to social avoidance and avoidance of bright spaces. Conversely, activation of DRN^5-HT∩vGluT3^→BA neurons inhibits two subsets of BA^Pyr^ neurons through 5-HT1A receptors (HTR1A) and 5-HT1B receptors (HTR1B). Pharmacological inhibition of HTR1A and HTR1B in the BA results in induces avoidance of bright spaces and social avoidance, respectively. These findings underscore the functional significance of heterogeneous inputs from the DRN to specific BA subpopulations in regulating distinct anxiety-related behaviors (Yu et al., 2022).

Second, it is widely accepted that stimulation of the axonal terminals of a projection selectively modulates the activity within that pathway and its downstream targets (Deisseroth, 2015; Emiliani et al., 2022). Specifically, optogenetic activation of presynaptic terminals can induce neurotransmitter release, thereby activating downstream circuits (Hsu et al., 2025). Accordingly, it is reasonable to speculate that activating the SNr^Glu^ neuronal terminals projecting to the DRN may engage downstream neural circuits responsible for regulating anxiety-related behaviors in the DRN through glutamate release. In line with this hypothesis, our current findings demonstrate that optogenetic activation of SNr glutamatergic neuronal terminals in the DRN significantly reduces anxiety levels in mice. However, the specific types of DRN neurons that form synaptic connections with SNr glutamatergic neurons warrant further investigation.

Finally, as the principal output center of the basal ganglia, the SNr projects to several brain regions. Consequently, optogenetic activation of the soma of SNr glutamatergic neurons constitutes a form of non-loop-specific stimulation, potentially influencing multiple downstream targets, including the caudate putamen (CPu), ventral posteromedial thalamic nucleus (VPM), zona incerta (ZID), and DRN. Thus, the modulation of anxiety levels or other behaviors in subjects under the activation of SNr glutamatergic neuron soma likely results from the interplay of multiple neural circuits, culminating in behavioral manifestations that reflect the integrated activity across various brain regions. Although our investigations indicate that the neural circuits linking the SNr to the aforementioned brain regions except for DRN does not correlate with social hierarchy regulation, we have not assessed its potential relationship with anxiety levels. The precise involvement of other neural circuits associated with SNr^Glu^ neurons in anxiety regulation remains ambiguous. Two possibilities arise: first, additional neural circuits may engage in anxiety regulation while potentially counteracting the SNr^Glu^-DRN circuit, thus inhibiting the emergence of anxiety-like behaviors upon optogenetic activation of SNr^Glu^ neurons; second, SNr^Glu^ neurons may not directly contribute to the regulation of anxiety behaviors, which would explain the absence of anxiety-like responses upon their activation. Certainly, these hypotheses warrant further investigation in future studies. Notably, prior research on the SNr has primarily concentrated on its roles in motor control and sleep-wake regulation, with limited literature addressing its potential involvement in anxiety modulation.

The following sentences have been added to Line 429,

“It is important to highlight that optogenetic activation of the SNr^Glu^ neurons and SNr^Glu^-DRN pathway produced distinct effects on anxiety, specifically effect-free responses versus alleviation. This discrepancy may stem from several factors. First, numerous studies have demonstrated the role of the DRN in modulating anxiety levels in mice[82-85]. Second, it is widely recognized that stimulation of the axonal terminals of a projection selectively influences the activity within that pathway and its downstream targets[86-88]. Accordingly, it is reasonable to speculate that activation of the SNr^Glu^-DRN pathway may engage downstream neural circuits responsible for regulating anxiety-related behaviors through glutamate release. However, the specific types of DRN neurons that form synaptic connections with SNr glutamatergic neurons require further investigation. Finally, the SNr^Glu^ neurons projects to several brain regions as identified in the current findings, including the CPu, VPM, ZID, and DRN. Optogenetic activation of SNr^Glu^ neurons may influence multiple downstream targets. Consequently, the absence of anxiety modulation following this activation may result from the ineffectiveness of these neurons or potential counteractions among various pathways. Future research should aim to elucidate the underlying mechanisms contributing to this phenomenon.”

***References for this answer (these references are included in the revised version)***

Deisseroth K. Optogenetics: 10 years of microbial opsins in neuroscience. *Nature Neuroscience*, 2015, 18(9): 1213-1225

Emiliani V, Entcheva E, Hedrich R, Hegemann P, Konrad KR, Lüscher C, Mahn M, Pan Z-H et al. Optogenetics for light control of biological systems. *Nature Reviews Methods Primers*, 2022, 2(1): 55

Hsu L-M, Cerri DH, Carelli RM, Shih Y-YI. Optogenetic stimulation of cell bodies versus axonal terminals generate comparable activity and functional connectivity patterns in the brain. *Brain stimulation*, 2025, 18(3): 822-828

Linley SB, Olucha‐Bordonau F, Vertes RP. Pattern of distribution of serotonergic fibers to the amygdala and extended amygdala in the rat. *Journal of Comparative Neurology*, 2017, 525(1): 116-139

Lowry CA, Johnson PL, Hay-Schmidt A, Mikkelsen J, Shekhar A. Modulation of anxiety circuits by serotonergic systems. *Stress*, 2005, 8(4): 233-246

Marcinkiewcz CA, Mazzone CM, D’Agostino G, Halladay LR, Hardaway JA, DiBerto JF, Navarro M, Burnham N et al. Serotonin engages an anxiety and fear-promoting circuit in the extended amygdala. Nature, 2016, 537(7618): 97-101

Yu X-D, Zhu Y, Sun Q-X, Deng F, Wan J, Zheng D, Gong W, Xie S-Z et al. Distinct serotonergic pathways to the amygdala underlie separate behavioral features of anxiety. Nature Neuroscience, 2022, 25(12): 1651-1663

Zheng X, Li DP, Yan X, Yao X, Wang Y. The role and mechanism of 5-HTDRN-BNST neural circuit in anxiety and fear lesions. *Frontiers in Neuroscience*, 2024, 18: 1362899

**Question 8.** The differing temporal profiles of optogenetic versus chemogenetic rank modulation raise important questions about the underlying mechanisms. The authors report that optogenetic effects persist for days while chemogenetic inhibition only transiently alters social status. How do the authors explain this discrepancy?

(**Question 8.** The authors write "The significantly reduced rankings compared to controls were sustained for approximately eight hours; however, by 24 hours post-CNO administration, most mice reverted to their original rank positions (Fig 3I-3K)." How do they explain the difference in modulating ranks across days by using opto- versus chemogenetic methods?)

**Answer:**

Yes, this is a compelling and significant question. In the present study, we observed that optogenetic activation has a more prolonged effect on social rank compared to chemogenetic activation. This finding aligns with previous studies that have demonstrated the enduring influence of optogenetic manipulation on social hierarchy alterations, which is more persistent than that induced by chemogenetic methods (Zhou et al., 2017, see Figures 2 and 3 in that reference, as further illustrated below; Fan et al., 2025). The establishment of a dominance hierarchy can be attributed to a reinforcing mechanism known as the “winner effect”, wherein animals exhibit an increased likelihood of winning following prior victories. This winner effect is underpinned by synaptic strengthening within the circuit connecting the mediodorsal thalamus (MDT) to the dorsomedial prefrontal cortex (dmPFC) as evidenced in the tube test (Zhou et al., 2017). In other words, synaptic plasticity within the MDT-dmPFC circuit is crucial to the winner effect in mammals. Further research is necessary to elucidate whether the synaptic plasticity mechanisms within the circuit associated with the SNr also contribute to the winner effect.

This raises an important question: Why does chemogenetic manipulation fail to induce significant synaptic plasticity in relevant circuits compared to optogenetic manipulation? The observed differences in the duration of effects can be attributed to at least two key factors: (1) Optogenetics offers millisecond-scale precision in neuronal activation, enabling researchers to target specific neural circuits at behaviorally relevant moments (e.g., during social competition). This precise timing can result in stronger and more enduring behavioral changes, as it directly influences neural activity at critical decision points, thereby facilitating the formation of synaptic plasticity. In contrast, while chemogenetics can modulate neuronal activity for extended periods, it lacks the temporal precision necessary to align with specific behavioral events. This prolonged but less specific activation may fail to engage synaptic plasticity within neural circuits in a behaviorally meaningful manner, resulting in weaker or shorter-lasting effects on social rank. (2) Furthermore, optogenetics typically achieves higher spatial specificity, activating only the intended neurons, whereas chemogenetic approaches may affect broader populations of neurons, potentially diminishing the intended behavioral effects over time. Consequently, optogenetics exerts a more lasting effect on social rank not due to a longer duration of neuronal activity, but rather because its precise timing during behaviorally relevant events fosters stronger synaptic plasticity within the associated neural circuitry. In contrast, despite its longer duration, chemogenetic activation lacks the temporal specificity essential for inducing robust and persistent changes in social rank.

The following sentences have been added to Line 397,

“Consistent with previous findings[25, 67, 68], the present results demonstrate a lasting impact of optogenetic manipulation on alterations in social hierarchy, particularly when compared to chemogenetic activation. The establishment of a dominance hierarchy can be attributed to a reinforcing mechanism known as the “winner effect”, in which prior victories increase the likelihood of future successes, thereby significantly influencing the dynamics of social hierarchy and competitive motivation. In mice, this winner effect can be mediated by synaptic strengthening within the circuit connecting the mediodorsal thalamus (MDT) to dmPFC[25]. Due to its millisecond-scale precision in neuronal activation, optogenetics likely facilitates the winner effect, which is supported by synaptic strengthening within the relevant neural circuits[25, 68]. Consequently, the enduring impact of optogenetics on social rank arises not from a prolonged duration of neuronal activity, but rather from its precise timing during critical behavioral events, enhancing synaptic plasticity within the associated neural circuitry. In contrast, although chemogenetic activation may have a longer-lasting effect, it lacks the temporal specificity necessary for inducing synaptic plasticity. Further research is warranted to investigate whether the mechanisms of synaptic plasticity within the circuit associated with the SNr also contribute to the winner effect.”

***References for this answer (this reference is included in the original version)***

Zhou, T.T., Zhu, H., Fan, Z.X., Wang, F., Chen, Y., Liang, H.X., Yang, Z.F., Zhang, L., Lin, L.N., Zhan, Y., et al. (2017). History of winning remodels thalamo-PFC circuit to reinforce social dominance. Science 357, 162-168.

Minor:

**Question 9.** Given that the SNr contains predominantly GABAergic neurons and the CaMKIIα promoter is known for leaky expression, it would be good if the authors could provide histological validation that viral expression was largely restricted to glutamatergic neurons (e.g., through co-labeling with VGLUT2 or the like).

(**Question 9.** Given that some of the cell population in the SNr is also GABAergic, and the CaMKIIa-promoter is known for its leaky expression, have the authors validated that the expression of the GCaMP and other viri is largely in SNr-Glu cells (eg post hoc staining/co-labelling with relevant markers).)

**Answer:**

We appreciate your valuable suggestions. In the revised version, we performed co-labeling with VGLUT2 antibody. The data indicate that viral expression in each group (EYFP, GCaMP6f, ChR2-EYFP, and hM4Di-EGFP) is predominantly confined to glutamatergic neurons within the SNr. These data can be found in S3 Fig in the Supporting Information.

The following sentence has been added to Line 118,

“Histological analyses demonstrated that GCaMP6f or EYFP localization was predominantly restricted to the SNr (S1A and S1B Fig). Additionally, co-labeling with CaMKIIα (Fig 1B; S2A and S2B Fig) or VGLUT2 antibodies (S3A and S3B Fig) confirmed that the majority of these neurons were glutamatergic.”

The following sentence has been added to Line 158,

“Histological analyses revealed that the localization of ChR2-EYFP was primarily confined to the SNr (S1C Fig). Furthermore, colocalization with CaMKIIα (Fig. 2B; S2C Fig) or VGLUT2 antibodies (S3C Fig) confirmed that the majority of these neurons were glutamatergic.”

The following sentence has been added to Line 223,

“Histological analyses indicated that hM4Di-EGFP localization was predominantly restricted to the SNr (S1D Fig). Moreover, co-labeling with CaMKIIα (Fig. 3B; S2D Fig) and VGLUT2 antibodies (S3D Fig) confirmed that the majority of these neurons were glutamatergic.”

We add the information of antibody “rabbit anti-VGLUT2 (1:500; Abcam, ab216463)” to Line 628.

The following sentences have been added to Line 633,

“Brain regions were identified according to the Mouse Brain Atlas[93], and the areas designated for quantification were delineated by outlining the boundaries of the SNr. Cell counting was conducted using Fiji software[94].”

**Question 10.** The exclusive focus on male mice should be explicitly acknowledged in the main text, particularly since the tube test has been successfully implemented in female C57 mice in other studies.

(**Question 10.** The tube test has been successfully implemented with female C57 mice. The focus of the authors on male mice only should be stated in the main text of the manuscript.)

**Answer:**

The original sentence in Line 29 “…social hierarchy in mice.” has been changed to “…in male mice.”

The original sentence in Line 36 “…social hierarchy.” has been changed to “…social hierarchy among male mice.”

The original sentence in Line 103 “…mice in the competitive tube test.” has been changed to “…male mice in the competitive tube test.”

The original sentence in Line 110 “…social hierarchy.” has been changed to “…social hierarchy in male mice.”

The original sentence in Line 395 “…in the regulating hierarchy dominance.” has been changed to “…in regulating hierarchy dominance in male mice.”

The original sentence in Line 423 “…and social dominance,” has been changed to “…and social dominance in male mice,”

**Question 11.** Several passages would benefit from clearer language, including: (a) replacing ambiguous terms like "behavioral displays" with more specific descriptions of behaviors, (b) revising "processes" to "neural mechanisms" when referring to hierarchy establishment, (c) changing "expression of dominant behaviors" to "dominance behaviors" for conceptual accuracy, and (d) correcting "aggressive levels" to "aggression levels" throughout for proper grammatical construction. These are just some examples where expression could be improved for clarity.

(**Question 11.** The language is not always clear.

(1) For instance, the authors write in the introduction that "This organizational framework delineates individual rankings within a group, established through recurrent social interactions such as aggressive encounters or behavioral displays". Here, it is not clear what type of behaviors "behavioral displays" refers to.

(2) The authors write "Despite this significance, the processes through which dominance hierarchies are established and maintained remain inadequately understood.", processes is ambigue here - the authors are here not assessing behavioral processes which give rise to dominance hierarchies, but study the underlying neural substrates.

(3) The authors write "and modulating the expression of dominant behaviors in downstream areas" - it should be "dominance behaviors". These are a few examples, but language requires a major revision.

(4) aggressive levels to aggression levels)

**Answer:**

We appreciate your suggestions and have thoroughly revised the entire text, addressing inappropriate word choices and correcting grammatical inaccuracies.

(1) We have changed “…behavioral displays” to “…behavioral displays that convey information regarding fighting ability or dominance status.”

(2) The original words “processes through which” have been changed to “neural mechanisms by which”;

(3) The original words “expression of dominant behaviors” have been changed to “dominance behaviors”.

The original words “expression of social hierarchies” (Lines 88, 379) have been changed to “representation of social status”.

(4) We have changed “aggressive levels” to “aggression levels”.

**Reviewer #2:**

**Question 1.** This manuscript entitled "The glutamatergic projection from the substantia nigra pars reticulate to the dorsal raphe nucleus facilitates social hierarchy in mice" investigates the potential involvement of glutamatergic neurons in the substantia nigra pars reticulate (SNr) projecting to the dorsal raphe nucleus (DRN) in regulating social dominance behavior, as assessed by the dominance tube test. Using fiber photometry, the authors demonstrate that SNr glutamatergic (Glu+) neurons are activated during push behavior, but not during mere traversal of the tube without an opponent. Optogenetic activation of SNr Glu+ neurons increased the frequency of push behavior and reduced retreat behavior, as well as enhanced locomotion in the open field test. Conversely, chemogenetic inhibition of SNr Glu+ neurons reduced push behavior, increased retreat, and reduced time spent in the warm spot in a competitive warm spot test. Lastly, the authors show that optogenetic activation of SNr-DRN Glu+ projections is sufficient to increase push behavior in the tube test. This study suggests a novel function of SNr-DRN pathway in effortful push behavior during social competition. However, additional experiments are necessary to support the conclusion that this pathway specifically facilitates social hierarchy.

**Answer:**

We sincerely appreciate your insightful comments and constructive suggestions. In the revised manuscript, we have implemented additional behavioral controls, updated the histological data, and addressed the statistical concerns raised, consistent with your suggestions and those of other reviewers and the editor.

**Question 2.** The authors showed a significant reduction in locomotor activity following DREADD-mediated inhibition of SNr Glu+ neurons. Given that the SNr is a well-known brain area involved in motor control (i.e. Partanen & Achim 2022, Front Neurosci), it is possible that the observed reduction in push behavior in the tube test or warm spot test is attributable to motor incoordination rather than a specific deficit in social dominance. To address this concern, please include additional behavioral assays assessing motor coordination (e.g. grip test, rotarod test) to rule out general motor deficits.

**Answer:**

(1) We used two groups of mice: the hM4Di-EGFP group and the EYFP group. Each mouse received two injections: one with saline and another with CNO, administered 24 hours apart. Relevant assessments were conducted two hours post-injection.

To assess motor coordination, we conducted both the pole test and the rotarod test in the revised manuscript. The pole test evaluates the grasping capabilities of the forelimbs and the overall motor coordination of mice. Before the experiment, the mice were transferred to the testing room for a period of 15 min to allow for acclimation. Each mouse underwent a two-day training regimen comprised of five trials per day, during which the animal was positioned head-up atop an upright wooden pole (50 cm in height and 1 cm in diameter) affixed to a square base, allowing for movement along the pole. On the third day, the mice were administered either CNO or saline and subsequently positioned head-up atop an upright wooden pole, two hours post-injection. In this test, the mice were subjected to three test trials, with the pole meticulously cleaned with alcohol after each individual test. We recorded the durations required for the mice to rotate from head-up to head-down and subsequently descend from the top to the bottom of the pole. Averages were calculated for subsequent statistical analysis.

In the pole test, the analysis of the duration taken by the mice to transition from head-up to head-down demonstrated no significant main effects for "injection (CNO vs. saline)" (n=9 for both the hM4Di-EGFP and EYFP groups; two-way repeated measures ANOVA with Bonferroni correction; F₁,₁_6_=0.067, *p*=0.799) or "group" (F₁,₁_6_=0.013, *p*=0.910). Similarly, the assessment of the total duration from head-up to descent to the bottom of the pole revealed no significant main effects for "injection" (F₁,₁_6_=0.007, *p*=0.936) or "group" (F₁,₁_6_=0.377, *p*=0.548). Collectively, these results indicate that the grasping capabilities of the forelimbs and overall motor coordination of mice were not influenced by DREADD-mediated inhibition of SNr^Glu^ neurons.

(2) The rotarod test was performed to evaluate locomotor activity and coordination. In the rotarod test, the mice are required to move on a rotating cylindrical rod. Before the experiment, the mice were transferred to the testing room for a period of 15 min to allow for acclimation. Subsequently, a two-day training regimen was implemented: the mice underwent training on the rotarod apparatus (XR-6C, Xinruan, Shanghai, China) at a low, constant speed of 5 rpm until they could maintain stability on the rotarod for 5 min without falling. During the test on the third day, the constant acceleration mode (from 4 to 40 rpm; acceleration time 5 min) was employed. The mice had to coordinate their movements and maintain their balance to ensure that they remained on the rod without falling off. If the mice fell, the latency, speed, and total distance traveled in the current trial were automatically recorded. Each mouse underwent three trials, with a minimum interval of 15 min between trials. The rod was meticulously cleaned with alcohol after each individual test. Averages were calculated for subsequent statistical analysis.

In the rotarod test, the analysis of latency revealed no significant main effects for "injection (CNO vs. saline)" (n=8 for both the hM4Di-EGFP and EYFP groups; two-way repeated measures ANOVA with Bonferroni correction; F₁,₁_4_=0.163, *p*=0.692) or for "group" (F₁,₁_4_=0.211, *p*=0.653). Similarly, the assessment of speed indicated no significant main effects for "injection" (F₁,₁_4_=0.705, *p*=0.415) or for "group" (F₁,₁_4_=0.990, *p*=0.337). Furthermore, the evaluation of distance showed no significant main effects for "injection" (F₁,₁_4_=0.137, *p*=0.717) or for "group" (F₁,₁_4_=0.163, *p*=0.693). Collectively, these findings suggest that locomotor activity and coordination in mice were unaffected by DREADD-mediated inhibition of SNr^Glu^ neurons.

The following paragraphs have been added to Line 572,

“**Pole Test.** The pole test assesses the grasping abilities of the forelimbs and overall motor coordination in mice. In this assay, mice were positioned head-up atop an upright wooden pole, measuring 50 cm in height and 1 cm in diameter, which was securely attached to a square base. Prior to the experiment, the mice were acclimated in the testing room for 15 minutes. We recorded the duration required for each mouse to rotate from a head-up to a head-down position and subsequently descend from the top to the bottom of the pole. Each mouse underwent a two-day training regimen, consisting of five trials per day. On the third day, the mice were subjected to three test trials, with the pole being meticulously cleaned with alcohol after each individual test. Averages were calculated for subsequent statistical analyses.

**Rotarod Test.** The rotarod test was conducted to evaluate locomotor activity and coordination in mice. In this assay, mice were required to traverse a rotating cylindrical rod. Prior to the experiment, the mice were acclimated in the testing room for 15 minutes. Subsequently, a two-day training regimen was implemented, during which the mice underwent training on the rotarod apparatus (XR-6C, Xinruan, Shanghai, China) at a low, constant speed of 5 rpm until they could maintain stability on the rod for 5 minutes without falling. On the third day, a constant acceleration mode (ranging from 4 to 40 rpm with an acceleration time of 5 minutes) was employed during the test. Mice were required to coordinate their movements and maintain balance to avoid falling from the rod. In the event of a fall, latency, speed, and total distance traveled during the trial were automatically recorded. Each mouse completed three trials, with a minimum interval of 15 minutes between trials. The rod was thoroughly cleaned with alcohol after each individual test, and averages were subsequently calculated for statistical analysis.”

The following sentences have been added to Line 256,

“To minimize the potential influence of motor incoordination induced by DREADD manipulation on mouse performance in the tube test, we conducted additional assessments using the pole test and rotarod test two hours post-injection of either CNO or saline. The pole test indicated no significant differences in the time taken for mice to transition from the head-up to head-down position, nor in the total duration from head-up to descent at the bottom of the pole, between CNO and saline injections or between hM4Di-EGFP and EYFP mice (S10A and S10B Fig). Similarly, the rotarod test revealed no significant differences in latency, speed, or total distance traveled between the CNO and saline injections, as well as between the hM4Di-EGFP and EYFP mice (S10C–E Fig). Collectively, these results suggest that motor coordination in mice were not affected by DREADD-mediated inhibition of SNr^Glu^ neurons.”

We have included a new S10 Fig in the Supporting Information, which presents the statistical results for both the pole test and the rotarod test.

**Question 3.** The relationship between increased push behavior following SNr activation and social dominance requires further clarification. It is possible that SNr Glu+ neurons are generally engaged during any form of effortful motor behavior, not necessarily behaviors associated with social competition. To address this, I recommend recording neural activity during a non-social, effortful task (e.g. pushing a non-animate object) using fiber photometry to determine whether activation of this area is specific to social dominance contexts.

**Answer:**

Thank you very much for your suggestion. In the revised version, we recorded SNr^Glu^ activity during both an active physical effort task that lacked social components (termed the push ball test, which involved pushing a diamond-shaped rubber bouncy ball within a tube) and a social, non-competitive effort task (designated as the social interaction test, involving interaction with a novel mouse introduced into a small elevated restraint cage). The results indicate that SNr^Glu^ neurons are not involved in effortful motor behaviors during the two types of tests conducted. Please refer to the answer to Question 1 from Reviewer #1.

**Question 4.** The finding that optogenetic activation of the SNr-DRN Glu+ projection enhances push behavior in the dominant tube test without affecting locomotor activity or forelimb muscle strength is promising. However, it remains unclear whether this projection is naturally involved in social dominance behavior. To support this, the authors should perform either optogenetic inhibition of the SNr-DRN projection during the dominance test or fiber photometry recording of this projection during dominance tube test. These experiments would clarify whether endogeneous activity in this pathway is related to dominance behavior.

**Answer:**

We sincerely appreciate your insightful suggestions. In a preliminary experiment utilizing optogenetic inhibition of SNr^Glu^-DRN pathway with four mice, we noted that exposure to yellow light stimulation significantly increased periods of immobility and reduced active competitive behavior in the test subjects, despite the eventual retreat of the opponent. It is plausible to speculate that opponents may be unable to detect the internal state changes in the subjects, potentially leading them to perceive the subjects' social rank as still superior, thereby diminishing their competitive motivation. Consequently, we employed a combined approach integrating both optogenetics and chemogenetics to bidirectionally modulate relevant brain regions and their associated circuits, in alignment with previous studies examining the regulation of social hierarchy in mice (Zhou et al., 2017; Xin et al., 2025; Zheng et al., 2025). Please refer to the response to Question 6 from Reviewer #1.

We conducted fiber photometry recordings of the SNr^Glu^ projection to DRN during the tube test; however, we observed no significant changes in Ca²⁺ signals during effortful behaviors in this context. Several factors may contribute to this finding. First, GCaMP exhibits weak expression in nerve terminals. As previously noted, “Genetically encoded calcium indicators (GECIs), such as the GCaMP family, have enabled recordings from the somata and dendrites of genetically defined neuronal populations in behaving animals...Untargeted GCaMPs (u-GCaMPs) preferentially label somatodendritic compartments and diffuse poorly to distal axons. Low expression levels of GECIs in presynaptic structures make even visualizing axons with expression difficult, and functional imaging of dimly labeled axons produces poor signal-to-noise ratios (SNRs), particularly deep in tissue where light scattering requires a high photon budget. Consequently, axonal GECI imaging is typically performed only under near-ideal conditions, such as shallow terminal fields of relatively short projections. Even so, low SNR and contamination from intermingled dendritic arbors closer to the expression source degrade imaging quality...” (Broussard et al., 2018). Second, while this neural circuit appears unrelated to the regulation of social hierarchy, this observation contradicts previous findings indicating that optogenetic activation of this circuit induces significant alterations in social hierarchy. Overall, the current evidence is insufficient to definitively ascertain whether this projection plays a natural role in social dominance behavior. As a result, we have downplayed the role of the SNr-DRN pathway in regulating social hierarchy in mice in our discussion and have documented the limitations of our findings in the revised version. Please refer to the answer to Question 3 from Reviewer #3.

The following sentences have been added to Line 437,

“However, it is crucial to acknowledge the limitations of this study. While the current findings suggest that optogenetic activation of the SNr^Glu^-DRN pathway is implicated in the regulation of social hierarchy, we have not acquired additional direct evidence to substantiate this conclusion. Further research is required to comprehensively elucidate the roles of this pathway, utilizing more advanced methodologies such as axon-specific calcium imaging with presynaptically targeted GCaMPs[89].”

***References for this answer (this reference is included in the original version)***

Broussard GJ, Liang Y, Fridman M, Unger EK, Meng G, Xiao X, Ji N, Petreanu L et al. In vivo measurement of afferent activity with axon-specific calcium imaging. Nature Neuroscience, 2018, 21(9): 1272-1280

**Question 5.** Figure 2 and 4. The authors state "n=24 for light off and n=48 for light on" (Fig. 2D-H) and "n=21 for light off and n=42 for light on" (Fig. 4C-E), but it is unclear how many biological replicates were used in these experiments. Please clarify the number of animals included in each condition. Additionally, it is unclear what exactly is the meant by "light off" and "light on", and why the sample sizes differ between these conditions. If these data represent a within subject comparison, the Wilcoxon signed-rank test should be used instead of Mann Whitney U test. Also, use the average value per animal if multiple data points were obtained from a single animal.

**Answer:**

In the original Figures 2 and 4, the variable *n* does not denote the number of animals but rather represents the total number of trials conducted under each condition. The terms "light off" and "light on" correspond to the respective conditions of light-off and light-on. The revised version no longer includes these two phrases. Indeed, these data reflect a within-subject comparison; thus, we calculate the average value per animal and apply the Wilcoxon signed-rank test instead of the Mann-Whitney U test in the revised version.

In comparison to the original results, although both the statistical values and *p*-values have been altered, the majority of differences remain unchanged. Notably, only the duration of stillness in the tube test, as illustrated in Figure 2, shifted from a non-significant to a significant status, whereas the time spent in the center during OFT transitioned from a significant to a non-significant status. Please refer to the updated Figures 2 and 4, along with their respective legends.

The original sentence in Lines 636-638,

“For the tube test, the Mann-Whitney U test was employed to compare differences in behavioral parameters across various light conditions and among different groups.”

has been changed to

“In the tube test, the Wilcoxon signed rank test was utilized to assess differences in behavioral parameters across various light conditions in optogenetic experiments, while the Mann-Whitney U test was employed to compare these parameters among different groups in chemogenetic experiments.”

**Reviewer #3:**

**Question 1.** Fan and colleagues present a well-written short report investigating the possibility that the relatively understudied glutaminergic neurons of the relatively understudied SNr (as opposed to SNc) may play a specific role in social hierarchy. They support this claim with fiber photometry of the SNr and manipulation of SNr neuronal activity -both via AAVs with the CamKIIa minimal promoter (CK2)- during the tube test, a test of social dominance. There is a lot to like about this study, not least that it is well-written. I do agree that the glutamatergic neurons of the SNr, and indeed the SNr itself, is understudied, and that finding a specific role for them in social hierarchy would be a novel and impactful result, as my understanding is the little that is known specifically about SNr has nothing to do with social interactions. It is an interesting question that to my knowledge nobody has looked at, and they have investigated it with both (admittedly low resolution) physiology and behavior levels. They definitely see behavioral effects of their manipulations, which they are able to modulate up and down, which is always nice. There is something there for sure, and it deserves to be investigated and reported properly.

**Answer:**

We sincerely appreciate your comments and suggestions.

**Question 2.** However, extraordinary claims need more data. My enthusiasm for the paper is limited precisely by the question of specificity, and it pertains to multiple levels. (1) Starting at the behavioral level, the authors are making the claim that their manipulations affect social hierarchy specifically, but almost the only behavioral task that they perform is the tube test, where you put 2 animals at different ends of a tube, and the one that doesn't back out is dominant. The behavioral data in this task seems to be clear, my issue is if all you look at is tasks that measure social hierarchy, then that is the only result that you will get. (2) I do not believe that their discussion of ancillary features of the tube test (e.g. stillness), precludes alternative interpretations of their behavioral results. They should candidly discuss other potential interpretations (anxiety, aggression) of their behavioral results and perform experiments that investigate them. For instance, given that they speak of anxiety effects (which are NOT the same as social effects, though they certainly interact), they need to see if for instance their manipulations are anxiogenic/lytic on other tasks, at the very least the elevated plus maze, which has no social aspects. To be able to say what something is, one needs to show what it is not.

**Answer:**

We sincerely appreciate your insightful comments and suggestions.

(1) Initially, we employed the tube test to evaluate alterations in the social hierarchy of mice, complemented by the warm spot test to further validate the findings from the tube test. This approach ensures the accuracy and generalizability of our results across various social contexts in mice. However, our initial experimental design could not completely exclude the possibility that activity in SNr^Glu^ neurons may correlate with all forms of effortful behavior, rather than being exclusively associated with social hierarchy. In other words, it is possible that SNr^Glu^ neurons are activated during any type of effortful motor behavior, not solely those linked to social competition. Consequently, in response to the editor's and reviewers' suggestions, we have incorporated two additional control experiments in the revised manuscript. We conducted an active physical effort task devoid of social components, termed the push ball test, which required subjects to push a diamond-shaped rubber bouncy ball within a tube, as well as a social, non-competitive effort task designated as the social interaction test, wherein interactions with a novel mouse introduced into a small elevated restraint cage were observed. During these tasks, we recorded SNr^Glu^ activity and performed optogenetic manipulations of the SNr and SNr^Glu^-DRN pathway. The results from these experiments further substantiate that SNr^Glu^ activity observed in this study is indeed related to social hierarchy. Please refer to the response to Question 1 from Reviewer #1 for additional details.

(2) Secondly, we elucidated through supplementary behavioral paradigms that the alterations observed in the tube test following optogenetic and chemogenetic manipulations were independent of other behavioral measures, including aggression (assessed via the Resident-intruder test), forelimb grip strength (measured using the grip-strength test), locomotor ability (evaluated through the open field and rotarod tests), and coordination (assessed with the pole test and rotarod test). Furthermore, we employed the elevated plus maze to evaluate anxiety levels in mice during optogenetic manipulation of the SNr^Glu^ neurons and its circuitry with DRN. Although our findings indicated that activation of SNr neurons did not alter anxiety levels, the stimulation of SNr glutamatergic terminals in the DRN was associated with a reduction in anxiety levels in the subjects. Nonetheless, these results do not entirely exclude the possibility that traversing the tube itself, due to factors such as the confined tube space, may influence anxiety levels. Specifically, it remains uncertain whether changes in anxiety stemming from tube traversal could partially account for observed behavioral patterns, such as immobility and withdrawal during the tube test. To mitigate this potential confound, we acclimated the mice to a 15 cm short tube placed within their housing cages one week prior to testing. Additionally, mice were pre-acclimated to the testing chamber ahead of the formal assessment. Furthermore, prior to the initiation of behavioral modulation or fiber optic recording, a daily tubing test lasting 4 to 7 days must be conducted for rank assessment. During this period, mice gradually acclimate to the restricted space within the tube. However, we acknowledge that we cannot completely dismiss the possibility that the tube test itself may evoke anxiety-related behavioral changes in mice, nor can we fully determine how such changes might influence their performance in the test. This necessitates further investigation through additional experiments. Accordingly, we have incorporated a dedicated section in the revised manuscript that specifically addresses these limitations of our study.

The following sentences have been added to Line 437,

“However, it is crucial to acknowledge the limitations of this study. While the current findings suggest that optogenetic activation of the SNr^Glu^-DRN pathway is implicated in the regulation of social hierarchy, we have not acquired additional direct evidence to substantiate this conclusion. Further research is required to comprehensively elucidate the roles of this pathway, utilizing more advanced methodologies such as axon-specific calcium imaging with presynaptically targeted GCaMPs[89]. Secondly, while we ensured that the subjects were thoroughly acclimated to the experimental environment prior to testing, we cannot entirely rule out the possibility that the tube test itself may elicit anxiety-related behavioral changes (e.g., immobility) in mice. Furthermore, we are unable to fully ascertain how such alterations may impact their performance during the test. This underscores the need for further investigation through additional experiments.”

***References for this answer (this reference is included in the original version)***

Broussard GJ, Liang Y, Fridman M, Unger EK, Meng G, Xiao X, Ji N, Petreanu L et al. In vivo measurement of afferent activity with axon-specific calcium imaging. Nature Neuroscience, 2018, 21(9): 1272-1280

**Question 3.** (1) I also find the anatomy kind of sloppy (i.e. not specific enough). The premise of the paper is that SNr glutaminergic neurons exert their effect via dorsal raphe, and while they are indeed connected, it is also true the DRN receives many other inputs (in the atlases they cite SNr is one of many inputs) and that the SNr projects many other places. They use DRN and SNr almost interchangeably throughout the paper, but nearly all measurements and manipulations are in the SNr. The connection to DRN should be downplayed, the current set of results does not sufficiently demonstrate this: the optogenetic stimulation of those fibers locally to the DRN are the only experiments which allow this claim, the chemogenetic ones could be acting elsewhere. (2) So far as I can tell, the basis for the claim of specific manipulations of glutamatergic SNr neurons are the viral promoter, the minimal CK2 promoter. As can even be seen from the panel they show, the overlap between reporter expression with this promoter in AAVs and CK+ neurons is not 100%, and depends upon many things like titre and the precise location of the injections, each of which being arguably unique. The authors need to count the overlap between the expression of their constructs and the SNr CK+ neurons for each animal, and also to know how much expression they got in CK2+ neurons in adjoining regions… that is a crowded neighborhood. Uploading images of each animal would be helpful in this regard, that is what supplementary data is for. I am not expecting overlap to be 100%, and their results would remain interesting even if mixed, but what was manipulated needs to be a known quantity of neurons.

**Answer:**

(1) This manuscript presents evidence for the involvement of SNr^Glu^ neurons in the regulation of social hierarchy in mice; however, evidence supporting the role of the SNr^Glu^-DRN pathway in this regulation remains insufficient. While experiments involving optogenetic activation of SNr glutamatergic terminals in the DRN offer preliminary support for the pathway's role in social hierarchy regulation, further studies are required to comprehensively validate its functional significance. Notably, we also performed optogenetic inhibition of both the SNr and the SNr-DRN pathway. These experiments demonstrated that exposure to yellow light stimulation resulted in significantly increased periods of immobility and reduced active competitive behavior in the test subjects, however the opponent ultimately retreated, potentially due to the winner effect or a lack of chemosensory signals reflecting the internal state changes in the test subject. Please refer to the answer to Question 6 from Reviewer #1.

We conducted fiber photometry recordings of the SNr^Glu^ projection to DRN during the tube test; however, we observed no significant changes in Ca²⁺ signals during effortful behaviors in this context. Please refer to the answer to Question 4 from Reviewer #2.

As a result, we have downplayed the role of the SNr-DRN pathway in regulating social hierarchy in mice within our discussion and have documented the limitations of our findings in the revised version.

The original sentence in Lines 34-35,

“Collectively, these results underscore the critical role of the glutamatergic SNr-DRN pathway in regulating social hierarchy.”

has been changed to

“Collectively, these results underscore the critical role of SNr glutamatergic neurons in regulating social hierarchy among male mice.”

The original sentence in Lines 108-110,

“Collectively, these findings highlight the pivotal role of the glutamatergic pathway from the SNr to the DRN in the regulation of social hierarchy.”

has been changed to

“Collectively, these findings highlight the potential role of the glutamatergic pathway from the SNr to the DRN in the regulation of social hierarchy in male mice.”

The original sentence in Lines 393-395,

“In this context, our study demonstrates that the SNrGlu-DRN pathway is involved in the regulating

hierarchy dominance.”

has been changed to

“In this context, our study demonstrates that the SNr^Glu^-DRN pathway may play a role in regulating hierarchy dominance in male mice.”

The original sentence in Lines 422-424,

“Collectively, these findings highlight the SNr^Glu^-DRN pathway as a pivotal neural circuit for modulating anxiety and social dominance, potentially…”

has been changed to

“Collectively, these findings highlight the SNr^Glu^-DRN pathway as a potential neural circuit for modulating anxiety and social dominance in male mice, thereby…”

(2) Yes. The overlap between reporter expression driven by this promoter in AAVs and CK+ neurons is not 100% in each subject, influenced by several factors including viral titer and the precise location of injection. Technical limitations, such as suboptimal sectioning or staining quality, resulted in not all brain sections meeting the criteria for neuronal co-labeling quantification. Consequently, we present data from four individual subjects per group regarding the percentage of SNr neurons expressing the virus relative to the total number of virus-expressing neurons in the entire field of view. Additionally, we provide data from two subjects per group on the percentage of viral-expressing neurons co-labeled with CaMKIIα, relative to the total number of viral-expressing neurons within the SNr. These data are detailed in S1 Fig and S2 Fig in the Supporting Information. To further validate the effective expression of the CaMKIIα promoter in glutamatergic neurons, we conducted immunofluorescence co-labeling with VGLUT2 in each group. In light of these findings, we have incorporated corresponding descriptions in the revised manuscript. Please refer to our response to Question 9 from Reviewer #1 for further details.

**Question 4.** The authors in sum present intriguing preliminary results that with further experimental investigation should become a quite impactful study. The authors seem to have found an interesting behavioral phenotype, though how specifically social it is remains to be determined. It would be a shame if they did not explore it more fully.

**Answer:**

We sincerely appreciate your constructive comments and suggestions. In the revised manuscript, we have implemented new behavioral controls, updated the histological data, and addressed the statistical concerns raised. These results further underscore the critical role of the SNr glutamatergic neurons in regulating social hierarchy in mice.
