# Supplementary material for: Glutamatergic projections from the substantia nigra pars reticulata to the dorsal raphe nucleus regulate male social hierarchies
Source: PLoS Biol. 2026 Mar 3;24(3):e3003687. doi: 10.1371/journal.pbio.3003687 (PMC12974815; doi:10.1371/journal.pbio.3003687)
Supplement: Attachment — Submitted filename: Response_to_Reviewers_auresp_3.docx [file pbio.3003687.s017.docx]

Dear Editor and Reviewer,

We appreciate the opportunity to revise and enhance our manuscript once more. We have carefully considered the provided comments and have made the necessary modifications to the manuscript, as detailed below. Textual changes are highlighted in blue.

Sincerely yours,

Yanzhu Fan

Guangzhan Fang

**Reviewer #3:**

**Question 1.** (1) The authors present a much improved document in the resubmission, with the addition of several necessary controls and data. The result is a provocative piece of work that I think nicely shows both a novel and interesting set of results and the limitations of behavioral analysis to parse the ramifications of the ever more specific manipulations made possible by modern molecular genetic techniques. In the end, the conclusion of this paper is basically that the glutaminergic neurons of the pars reticulata, unlike its more numerous GABAergic neurons (let alone compacta) which are primarily involved in motor control, play a role specifically in social hierarchy via their innervation of the DRN. That is a remarkable result, really…I would have expected a much more general change (and indeed, there is definitely some crosstalk with neural systems underlying anxiety, see below), but all in all, the authors have done the appropriate controls and the data supports at least a privileged role for this particular projection in social hierarchy.

(2) I personally think they have done sufficient numbers of controls to uphold their main conclusion. All I would ask the authors to do would be to acknowledge the issues and limitations that are indeed inherent to interpreting the behavioral ramifications of network-level manipulations, lest we fall prey to a new kind of phrenology (i.e. this synapse does this, that synapse does that). We all agree to interpret certain behavioral tasks in certain reasonable ways, but need to keep in mind there is some distance between what we measure and the psychological terms we associate with those measurements. In this case, what is clear is their manipulations definitely change the state of the animal in some way that shows up best in those behavioral tests we agree measure social hierarchy and anxiety, but that is not the same as saying this is a pathway that is specific to those ethological and psychological constructs. I know this seems like splitting hairs, but I think it is important to make it explicit.

How to do this? Well, I would say in two ways. First, although they have climbed back a bit from the prior submission, there is still some pretty strong language throughout the document which might lead the reader to think that social hierarchy is all these neurons do, which I don't believe is the authors' intention.

Below is one, but probably not the only example:

"Collectively, these results underscore the critical role of SNr the glutamatergic neurons SNr-DRN pathway in regulating social hierarchy among male mice." (abstract)

(3) And here's one that says the same thing but doesn't trigger that reaction:

"Collectively, these findings highlight the potential role of the glutamatergic pathway from the SNr to the DRN in the regulation of social hierarchy in male mice." (Intro)

**Answer:**
(1) We sincerely appreciate your comments and suggestions.

(2) The original sentence in the abstract,

“Collectively, these results underscore the critical role of SNr glutamatergic neurons in regulating social hierarchy among male mice.”

has been changed to

“Collectively, these results broaden our understanding of the functions associated with SNr glutamatergic neurons and underscore their critical role in regulating social hierarchy among male mice.”

(3) The original sentence in the section Introduction,

“Collectively, these findings highlight the potential role of the glutamatergic pathway from the SNr to the DRN in the regulation of social hierarchy in male mice.”

has been changed to

“Collectively, these findings expand our understanding of the functions associated with the glutamatergic pathway from the SNr to the DRN, highlighting its potential role in the regulation of social hierarchy among male mice.”

**Question 2.** Second, while there are clear examples of them trying (e.g. Line 437), there needs to be a paragraph that reflects the difficulties in interpretation that arise from jumping from the level of stimulating a particular set of neurons to an ethological phenotype. A frank discussion of the fact that, as in their data, anxiety and social position are intertwined, and there are limitations to the interpretation of these tasks from behavioral correlates alone. This leads into their stated plans to investigate these phenomena at a more neurophysiological level, so it shouldn't be hard to incorporate.

Below is the closest discussion part:

"Consistent with these observations, the current findings indicate that the activation of glutamatergic projections from the SNr to the DRN not only enhances social status in mice but also alleviates anxiety levels. This suggests a mutually reinforcing relationship between low-anxious status and the acquisition of dominance. Collectively, these findings highlight the SNr^Glu^-DRN pathway as a potential neural circuit for modulating anxiety and social dominance in male mice, thereby providing insight into the intricate relationship between social hierarchy and anxiety levels[13]. This relationship may be mediated through distinct neural circuits or receptor mechanisms, emphasizing the need for further research to elucidate the circuitry and synaptic mechanisms underpinning the intrinsic connections between social hierarchy and anxiety levels in mice."

To me, this paragraph still keeps the social aspect of the manipulation paramount… it is entirely possible that it changes in anxiety (or more correctly whatever changes in the brain we measure in those tests) that is driving the social aspect of these changes, that needs to be explicit, or they need to explicitly examine this and make a convincing counter-argument otherwise. They have definitely found something interesting, though, and I congratulate them on that. The differences they obtained regarding local optogenetic manipulation at DRN versus at the SNr are perhaps the most interesting part, in my opinion, as they hint at the complexity of the roles played by these relatively few neurons.

**Answer:**

We sincerely appreciate your valuable comments and suggestions.

One of the major goals of neuroscience research is to reveal the brain’s functional architecture to build a theoretical framework that bridges the brain and behavior (Schall 2004). While alternative perspectives, such as ethologically-based theories, have been proposed (Cisek and Kalaska, 2010), discussions regarding the neural underpinnings of behavior are frequently framed within an information processing model. In this framework, the brain is seen as an information processing system that first transforms sensory information into perceptual representations, then uses these to construct knowledge about the world and make decisions, and finally implements decisions through action. When we ask ‘‘How does the brain generate behavior?’’, we are primarily asking about how putative processing modules are organized so that they combine to generate behavior in a particular task environment (Krakauer et al., 2017). Thus, animal behavior serves as the macroscopic manifestation of neural activity, and the challenge lies in employing meticulously designed experiments and theoretical frameworks to translate quantitative, contextual, and high-dimensional measurements into high-level, universal explanations of animal behavior—effectively a journey from data to meaning (Gomez-Marin et al., 2014).

However, it is evident that inferring the relationship between a system's behavior and its lower-level properties—such as the genome, cell types, and connectome—presents significant challenges (Krakauer et al., 2017). Specifically, difficulties arise when attempting to transition from activating a particular set of neurons to understanding an ethological phenotype due to several factors. First, although emerging methods, such as optogenetics, enable the determination of certain causal relationships between brain activity and behavior, it is essential to recognize that causal-mechanistic explanations are qualitatively different from understanding how component modules perform the computations that then combine to produce behavior (Krakauer et al., 2017). A detailed examination of brain parts or their selective perturbation is not sufficient to understand how the brain generates behavior. One reason is that we have no prior knowledge of what the relevant level of brain organization is for any given behavior (Krakauer et al., 2017). The same behavior may result from alternative circuit configurations (Marder and Goaillard, 2006), from different circuits altogether or the same circuit may generate different behaviors (Katz, 2016). Consistent with this perspective, the interplay between anxiety and social hierarchy is presented in the present study, indicating limitations in interpreting these tasks based solely on behavioral correlates. Second, a granularity mismatch exists between neural and behavioral data, hindering substantive alignment across different levels of description (Poeppel and Embick, 2005). Finally, neural studies have often reduced behavioral complexity by favoring highly controlled experimental setups, allowing researchers to concentrate on the intricacies of the brain itself (Gomez-Marin et al., 2014). However, behavior is as complex as the nervous system; it results not only from the brain but also from its interactions with an ever-changing and complex environment, despite efforts to control for these factors in the experiments (Gomez-Marin et al., 2014).

The last sentence in the paragraph mentioned by the reviewer (I apologize that we forgot to add line numbers in the previous version of the manuscript),

“This relationship may be mediated through distinct neural circuits or receptor mechanisms, emphasizing the need for further research to elucidate the circuitry and synaptic mechanisms underpinning the intrinsic connections between social hierarchy and anxiety levels in mice.”

has been changed to

“However, we are unable to fully disentangle the causal relationships among neural pathways, social hierarchy, and anxiety levels. This challenge stems from the difficulties involved in transitioning from the activation of a specific set of neurons to the understanding of an ethological phenotype[82]. Contributing factors include the distinction between causal-mechanistic explanations and the neural mechanisms that underpin behavior[82], the tendency of experimental designs to oversimplify behavioral complexity[83], and a granularity mismatch between neural and behavioral data[84]. Furthermore, the interwoven relationship between anxiety and social hierarchy observed in our results further complicates interpretation. Considering that anxiety may regulate individuals' competitiveness[76], we cannot discount the possibility that alterations in anxiety levels or other physiological states induced by experimental manipulation could result in changes in social hierarchy. This relationship may be mediated through distinct neural microcircuits or receptor mechanisms, emphasizing the need for further research to elucidate the circuitry and synaptic mechanisms underpinning the intrinsic connections between social hierarchy and anxiety levels in mice.”

***References for this answer***

Cisek P, Kalaska JF. Neural mechanisms for interacting with a world full of action choices. Annu Rev Neurosci. 2010; 33:269-98.

Gomez-Marin A, Paton JJ, Kampff AR, Costa RM, Mainen ZF. Big behavioral data: psychology, ethology and the foundations of neuroscience. Nat Neurosci. 2014;17(11):1455-62.

Goette L, Bendahan S, Thoresen J, Hollis F, Sandi C. Stress pulls us apart: Anxiety leads to differences in competitive confidence under stress. Psychoneuroendocrinology. 2015; 54:115-23.

Katz PS. Evolution of central pattern generators and rhythmic behaviours. Philos Trans R Soc B‌. 2016;371(1685) :20150057.

Krakauer JW, Ghazanfar AA, Gomez-Marin A, MacIver MA, Poeppel D. Neuroscience needs behavior: correcting a reductionist bias. Neuron. 2017;93(3):480-90.

Marder E, Goaillard J-M. Variability, compensation and homeostasis in neuron and network function. Nat Rev Neurosci. 2006;7(7):563-74.

Poeppel D, Embick D. Defining the relation between linguistics and neuroscience. In: Cutler AE, editor. Defining the Relation between Linguistics and Neuroscience Twenty-First Century Psycholinguistics: Four Cornerstones: Lawrence Erlbaum; 2005. p. 103–18.

Schall JD. On building a bridge between brain and behavior. Annu Rev Psychol. 2004; 55: 23-50.

**Question 3.** Finally, while this may seem counterintuitive given the above, I would suggest that they have perhaps toned down the language a bit too much in the end of the discussion… yes, its true, more experiments are needed, but these are interesting results!

**Answer:**

The sentences in the end of the discussion

“However, it is crucial to acknowledge the limitations of this study. While the current findings suggest that optogenetic activation of the SNr^Glu^-DRN pathway is implicated in the regulation of social hierarchy, we have not acquired additional direct evidence to substantiate this conclusion. Further research is required to comprehensively elucidate the roles of this pathway, utilizing more advanced methodologies such as axon-specific calcium imaging with presynaptically targeted GCaMPs. Secondly, while we ensured that the subjects were thoroughly acclimated to the experimental environment prior to testing, we cannot entirely rule out the possibility that the tube test itself may elicit anxiety-related behavioral changes (e.g., immobility) in mice. Furthermore, we are unable to fully ascertain how such alterations may impact their performance during the test. This underscores the need for further investigation through additional experiments.”

has been changed to

“For instance, the current findings suggest that optogenetic activation of the SNr^Glu^-DRN pathway is implicated in the regulation of social hierarchy, however, further research is required to comprehensively elucidate the roles of this pathway, utilizing more advanced methodologies such as axon-specific calcium imaging with presynaptically targeted GCaMPs[89]. Additionally, although we ensured that the subjects were thoroughly acclimated to the experimental environment prior to testing, we cannot entirely rule out the possibility that the tube test itself may elicit anxiety-related behavioral changes (e.g., immobility) in mice. Consequently, we are unable to fully ascertain how such alterations may impact their performance during the test. Therefore, further investigation is imperative to elucidate the relationship between the tube test, anxiety levels, and animal performance, underscoring the need for additional experimental studies.”
